# Deoxygenative C2-heteroarylation of quinoline *N*-oxides: facile access to α-triazolylquinolines

**DOI:** 10.3762/bjoc.17.42

**Published:** 2021-02-17

**Authors:** Geetanjali S Sontakke, Rahul K Shukla, Chandra M R Volla

**Affiliations:** 1 Department of Chemistry, Indian Institute of Technology Bombay, Powai-400076, Mumbai, India

**Keywords:** amination, heteroarylation, quinoline *N*-oxides, regioselective, triazoles

## Abstract

A metal- and additive-free, highly efficient, step-economical deoxygenative C2-heteroarylation of quinolines and isoquinolines was achieved from readily available *N*-oxides and *N*-sulfonyl-1,2,3-triazoles. A variety of α-triazolylquinoline derivatives were synthesized with good regioselectivity and in excellent yields under mild reaction conditions. Further, a gram-scale and one-pot synthesis illustrated the efficacy and simplicity of the developed protocol. The current transformation was also found to be compatible for the late-stage modification of natural products.

## Introduction

**Figure 1:**
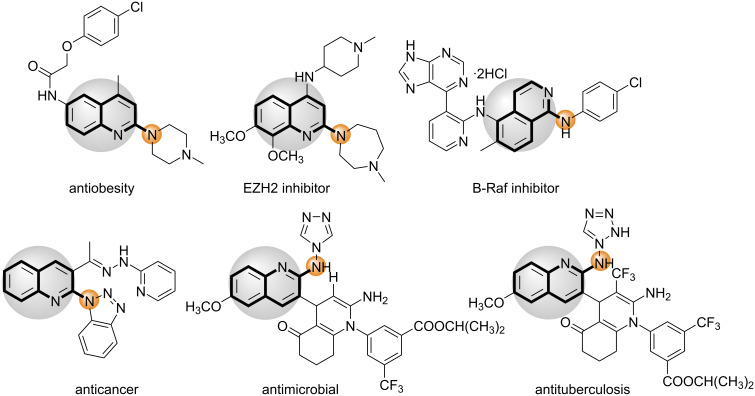
Bioactive molecules containing the 2-aminoquinoline motif.

Quinoline is a key heterocyclic moiety found in many natural products [1–4], agrochemicals and pharmaceuticals having potent biological activities, such as antimalarial, antibacterial and anticancer activities [5–11]. Due to their wide range of applications, selective functionalization of quinolines at various positions has gained significant interest in the last few years [12–13]. In particular, the C2-functionalization of quinolines has been well studied, and various methodologies were established for C–C [14–23], C–O [24–25] and C–S [26–27] bond formation. Recently C2-selective C–N bond formation has also attracted considerable attention due to the importance of the 2-aminoquinoline motif in medicinal chemistry and pharmaceuticals [28–45]. Some of the representative examples of biologically relevant derivatives containing a 2-aminoquinoline motif are shown in Figure 1 [46–47].

**Scheme 1:**
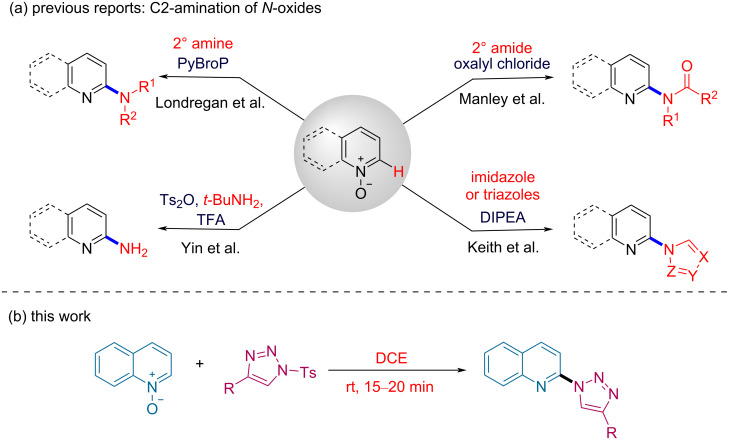
C2-selective C–N bond formation of *N*-oxides.

As a result, a variety of transition metal-catalyzed C2-selective aminations of azine *N*-oxides were explored extensively [28–34]. Additionally, various transition metal-free C2-aminations of these scaffolds were also investigated [35–45]. For example, Yin and co-workers developed a protocol for the deoxygenative C2-amination of pyridine/quinoline *N*-oxides using *t*-BuNH_2_ and Ts_2_O/TFA in 2007 (Scheme 1a) [48].

Later, Londregan and co-workers were successful in achieving C2-amination employing secondary amines as the nucleophiles [49]. In their protocol, the regioselectivity is controlled by PyBroP catalyst, which acts as the *N*-oxide activator. Manley and Bilodeau reported the amidation of heterocyclic *N*-oxides at the C2-position using oxalyl chloride for activating the *N*-oxides [50]. In addition to amination and amidation, there are few reports on metal-free C2-heteroarylation of pyridine *N*-oxides. In 1984, Rogers demonstrated the synthesis of 2-triazolylpyridines from 2-azidopyridines and phenylacetylene [51]. Along the same lines, Keith reported methodologies for the C2-imidazolylation and triazolylation of various heterocyclic *N*-oxides using sulfuryldiimidazole and 1,2,4-triazole, respectively. However, their protocol affords a mixture of C2- and C4-heteroarylated products [52–53]. More recently, Muthusubramanian and co-workers further expanded the scope of Keith’s protocol to a variety of pyridine *N*-oxides [54]. Despite the versatility of these methods, the above reports involve the use of external additives for activating the *N*-oxides and suffer from other disadvantages, including prolonged reaction time, high temperature and limited substrate scope. At the same time, with the advent of Cu-catalyzed “Click” chemistry, *N*-sulfonyl-1,2,3-triazoles have become useful precursors for accessing a variety of heterocyclic moieties [55–56].

In spite of the above methods for the C2-amination, the establishment of a simple, efficient and atom-economical method for the synthesis of 2-triazolylquinoline derivatives is highly desired. The continuous interest and efforts of our group for the derivatization of quinoline moieties [57] and use of *N*-sulfonyl-1,2,3-triazoles as heterocyclic precursors encouraged us to develop a new strategy for the regioselective C2-triazolylation of quinoline *N*-oxide under mild reaction conditions (Scheme 1b) [58–62].

## Results and Discussion

We initiated our trials employing easily accessible quinoline *N*-oxide (**1a**) and 1-tosyl-4-phenyl-1,2,3-triazole (**2a**) as model substrates. Subjecting the reaction mixture to 100 °C in the presence of DIPEA in 1,2-dichloroetane (DCE), we were pleased to observe the formation of C2-triazolylquinoline **3a** selectively in 15% NMR yield (Table 1, entry 1). Replacing DIPEA with DABCO or K_2_CO_3_ did not further increase the yield of **3a** (Table 1, entries 2 and 3). Interestingly, while doing the control studies to understand the role of the base, we found that the reaction indeed proceeds in the absence of the base to afford **3a** in a better yield (40%, Table 1, entry 4). We further optimized the reaction conditions by lowering the temperature to 80 °C and then to 60 °C, which afforded **3a** with a moderate yield increment up to 52% (Table 1, entries 5 and 6). However, when the reaction was performed at room temperature, surprisingly, the yield of **3a** increased to 95% within 15 minutes (Table 1, entry 7). To examine the influence of solvent on the reaction, we screened other solvents, such as DCM, CHCl_3_ and toluene, which provided **3a** only in inferior yields (Table 1, entries 8–10).

**Table 1:** Optimization of the reaction conditions.^a^

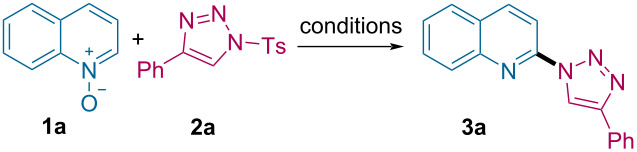				

entry	solvent	base	*T* (°C)	yield (%)^b^

1	DCE	DIPEA	100	15
2	DCE	DABCO	100	8
3	DCE	K_2_CO_3_	100	<5
4	DCE	–	100	40
5	DCE	–	80	45
6	DCE	–	60	52
7	DCE	–	rt	95 (92)^c^
8	DCM	–	rt	78
9	chloroform	–	rt	65
10	toluene	–	rt	58

^a^Reaction conditions: 0.1 mmol of **1a** and 0.12 mmol of **2a** in 1 mL solvent. ^b^NMR yield was taken by using 0.1 mmol of 1,3,5-trimethoxybenzene as an internal standard. ^c^Yield of the isolated product in parentheses for 0.2 mmol **1a**.

**Scheme 2:**
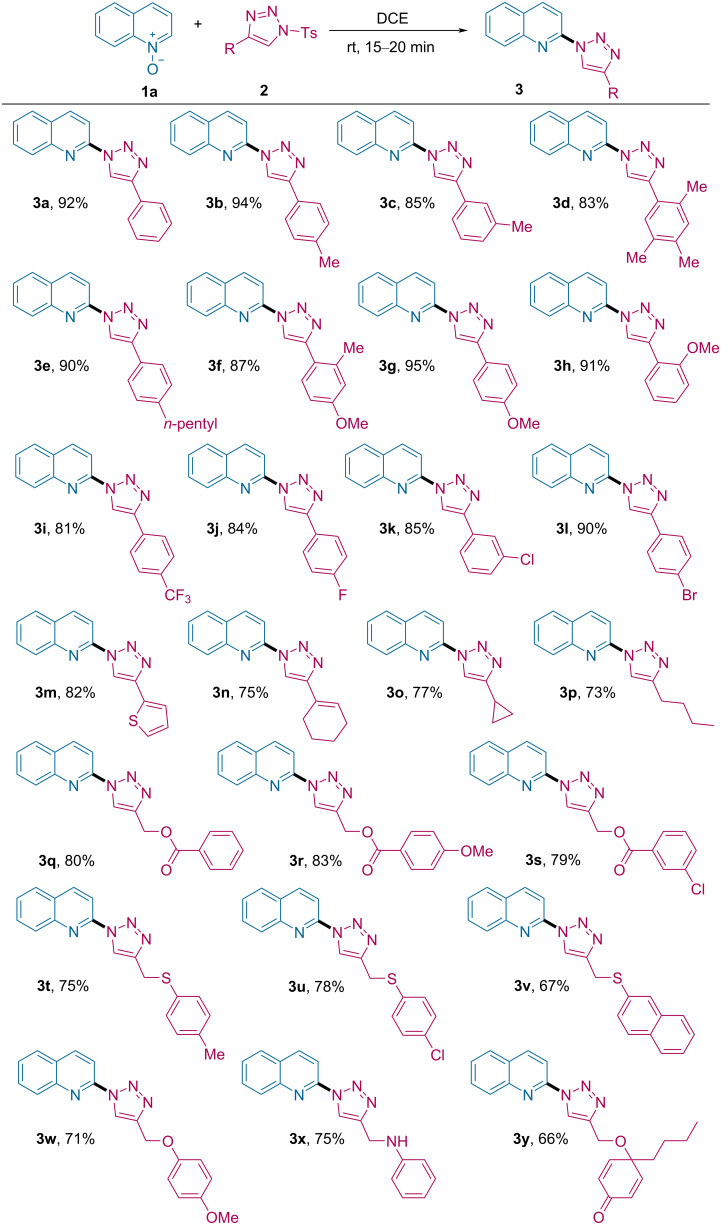
Substrate scope of *N*-sulfonyl-1,2,3-triazoles. Reaction conditions: **1a** (0.2 mmol), **2** (0.24 mmol) and DCE (2 mL) at rt for 15–20 min.

Using the optimized reaction conditions, we explored the substrate scope of our developed strategy using quinoline *N*-oxide (**1a**) with triazoles **2** having variable functional groups (Scheme 2). We examined the impact of electronic and steric effects of the substituents and observed that triazoles bearing electron-donating groups on the aromatic ring, such as alkyl or methoxy moieties, were well tolerated, furnishing the corresponding products **3b**–**h** in 83–95% yield. For electron-poor substituents (CF_3_ and F) on the aryl ring of the triazole, the corresponding products **3i** and **3j** were isolated in slightly lower yield (81% and 84%, respectively). Triazoles bearing halide substituents (Cl and Br) at the *meta*- or *para*-positions were also found to be viable substrates to afford the products **3k** and **3l** with 85–90% yield. Additionally, the reaction protocol is compatible with the heteroaromatic substrates and afforded **3m** in 82% yield.

Gratifyingly, alkenylated and aliphatic triazoles also showed similar reactivity and furnished desired products **3n**–**p** in moderate yield (73–77%). Motivated by the above results, we further extended the scope of triazoles by using different heteroatom-tethered triazoles. The -O-, -S- and -N-linked triazoles smoothly reacted under standard reaction conditions to provide the corresponding functionalized C2-triazolylquinolines **3q**–**x** in 67–83% yield. Cyclohexadienone-tethered triazole afforded the corresponding product **3y** in 66% yield. It is worth mentioning that when pyridine *N*-oxide was employed instead of **1a**, only traces of the corresponding product were observed even after prolonged reaction time.

**Scheme 3:**
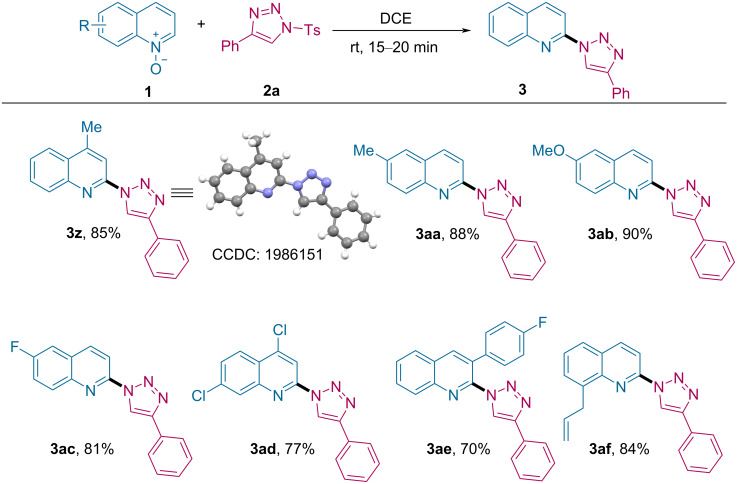
Substrate scope of quinoline *N*-oxides. Reaction conditions: **1** (0.2 mmol), **2a** (0.24 mmol) and DCE (2 mL) at rt for 15–20 min.

Subsequently, the substrate scope was evaluated by analyzing electronic and steric effects of substituents present on the quinoline *N*-oxides **1** (Scheme 3). Electronically rich Me and OMe groups at the 4- or 6-positions of *N*-oxides were tolerated and delivered the corresponding C2-triazolylquinolines **3z**, **3aa** and **3ab** in 85–90% yield. Further, the structure was unambiguously confirmed by the single-crystal X-ray diffraction analysis of **3z** [63]. Halogenated substrates also furnished the 2-heteroarylated products **3ac** and **3ad** in 81% and 77% yield, respectively. For 3-substituted quinoline *N*-oxides, a slight decrease in the yield to 70% was observed for **3ae**. The 8-allylated quinoline *N*-oxide cleanly provided **3af** in 84% yield.

**Scheme 4:**
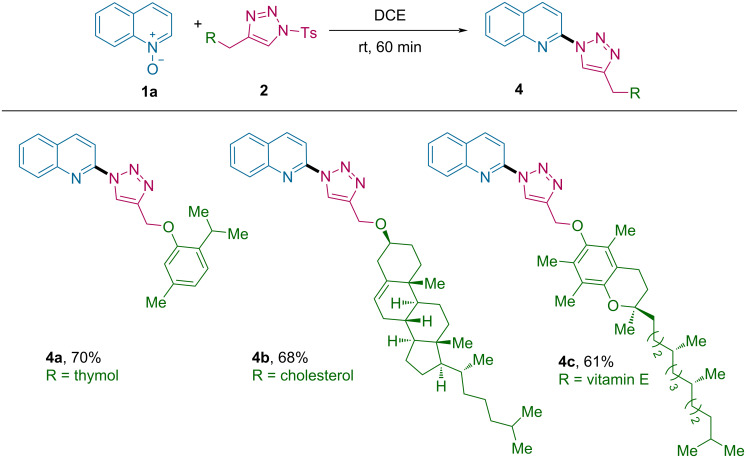
Late-stage modification of natural products.

In addition, the compatibility of this transformation for late-stage modification of complex natural products was explored, and as shown in Scheme 4, the protocol was tested with various natural-product-derived triazoles. Novel C2-triazolyl products **4a**–**c** were synthesized in good yield of 61–70% from triazoles derived from thymol, cholesterol and vitamin E, respectively.

**Scheme 5:**
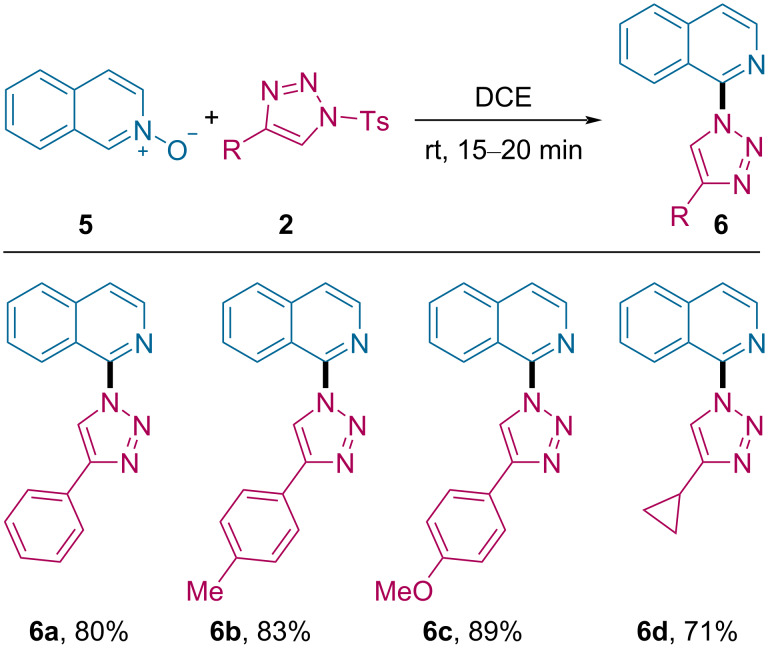
Substrate scope for the reaction of substituted triazoles with isoquinoline *N*-oxide.

To our delight, this protocol could also be extended for selective C1-functionalization of isoquinoline *N*-oxide (**5**, Scheme 5). Treating **5** with triazole **2a** under standard reaction conditions, the C1-heteroarylated isoquinoline product **6a** was obtained in 85% yield. Both the aromatic and aliphatic triazoles were suitable substrates for the reaction and afforded **6b**–**d** in good to excellent yield of 71–89%.

**Scheme 6:**
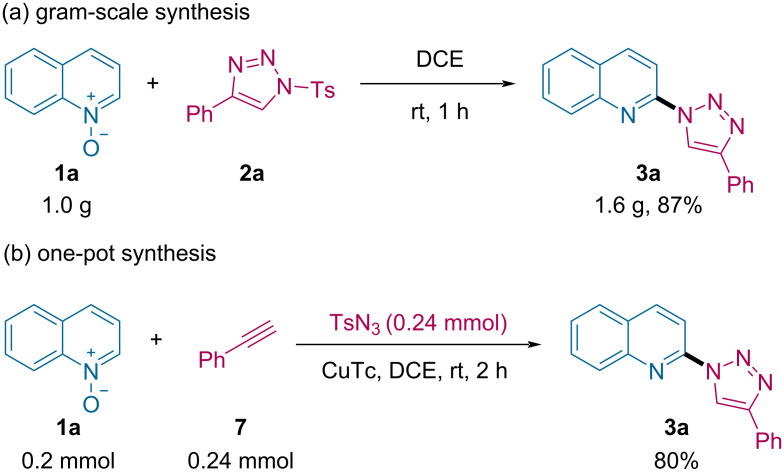
Gram-scale and one-pot synthesis.

In order to investigate the synthetic utility of the current methodology, a gram-scale reaction was carried out (Scheme 6a). Upon treating quinoline *N*-oxide (**1a**, 6.9 mmol, 1.0 g) with 4-phenyl-1-tosyl-1*H*-1,2,3-triazole (**2a**, 8.3 mmol, 1.2 equiv) at room temperature for 1 h, the reaction worked equally well and produced the desired product **3a** in 87% yield (1.6 g). In addition, to enlarge the simplicity of the developed protocol for selective C2-triazolylation of quinoline derivatives, we performed a sequential one-pot synthesis by combining a Cu(I)-catalyzed “Click” reaction of phenylacetylene (**7**) with TsN_3_ and a metal-free C2-heteroarylation of quinoline *N*-oxide (**1a**, Scheme 6b). Remarkably, the yield of the desired product **3a** in the one-pot synthesis (80%) was comparable to the stepwise pathway.

**Scheme 7:**
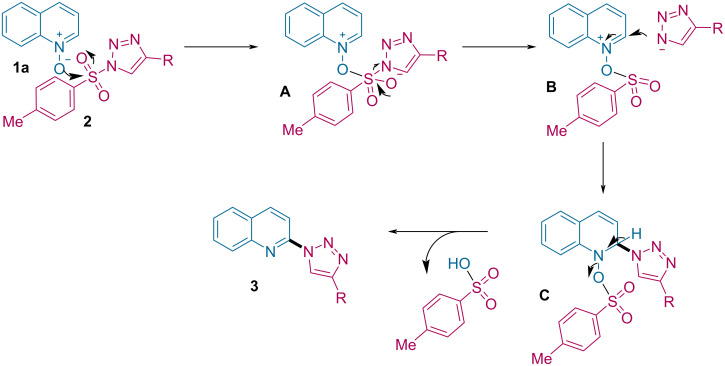
Proposed mechanism.

The plausible mechanism for the C2-triazolylation of quinoline *N*-oxides is presented in Scheme 7 [64]. The reaction initiates by the nucleophilic attack of quinoline *N*-oxide, e.g., **1a**, on the sulfonyl group of *N*-sulfonyl-1,2,3-triazole **2**, leading to the formation of intermediate **A**. This on elimination of a free triazolyl anion generates intermediate **B**. In next step, the triazolyl anion attacks the electrophilic C2-position of the quinoline *N*-oxide, providing the intermediate **C**, which, upon rearomatization, affords the desired 2-triazolylquinoline product **3**, along with the transfer of the oxygen atom from quinoline to the sulfonyl group of the triazole to form *p*-toluenesulfonic acid as the byproduct.

## Conclusion

In summary, we have developed an operationally simple and metal-free protocol for the synthesis of α-triazolylquinolines utilizing *N*-sulfonyl-1,2,3-triazoles as the aminating source. The reaction proceeds at room temperature in a short reaction time with excellent regioselectivity. The methodology was also found to be compatible with isoquinoline *N*-oxides and diversely functionalized *N*-sulfonyl-1,2,3-triazoles to establish a broad substrate scope for a variety of scaffolds. The late-stage modification of natural products is the salient feature of this current transformation.

## Experimental

### General procedure for the C2-selective synthesis of α-triazolylquinolines **3**

To an oven-dried reaction tube equipped with a magnetic stirring bar were added quinoline *N*-oxide (**1a**, 29 mg, 0.2 mmol, 1.0 equiv) and 4-phenyl-1-tosyl-1*H*-1,2,3-triazole (**2a**, 72 mg, 0.24 mmol, 1.2 equiv), followed by DCE (2 mL) via syringe. The reaction mixture was allowed to stir at rt for 15–20 min. After completion of the reaction, the solvent was evaporated under reduced pressure, and the residue was purified by column chromatography (ethyl acetate/petroleum ether 1:9) to get the desired product **3a** in 92% yield (50 mg, 0.18 mmol).

## Supporting Information

File 1 Experimental details.
