# Supplementary material for: Deoxygenative C2-heteroarylation of quinoline N-oxides: facile access to α-triazolylquinolines
Source: Beilstein J Org Chem. 2021 Feb 17;17:485–93. doi: 10.3762/bjoc.17.42 (PMC7934756; doi:10.3762/bjoc.17.42)

BEILSTEIN JOURNAL OF ORGANIC CHEMISTRY

Supporting Information

for

Deoxygenative C2-heteroarylation of quinoline *N*-oxides: facile access to α-triazolylquinolines

Geetanjali S. Sontakke, Rahul K. Shukla and Chandra M. R. Volla

Beilstein J. Org. Chem. **2021,** *17,* 485–493. doi:10.3762/bjoc.17.42

Experimental details

License and Terms: This is a supporting information file under the terms of the Creative Commons Attribution License (https://creativecommons.org/licenses/by/4.0). Please note that the reuse, redistribution and reproduction in particular requires that the author(s) and source are credited and that individual graphics may be subject to special legal provisions.
The license is subject to the *Beilstein Journal of Organic Chemistry* terms and conditions: (https://www.beilstein-journals.org/bjoc/terms)

Table of contents

Experimental section	S2
General information and methods	S2
Experimental procedures	S2
General procedure for the synthesis of quinoline *N*-oxides	S2
General procedure for the synthesis of *N*-sulfonyl-1,2,3-triazoles	S2
General procedure of α-triazolylquinoline	S3
Spectroscopic data of the compounds	S3
References	S24
^{1}H and ^{13}C spectra of compounds	S25

Experimental section

General information and methods

All reactions were carried out under nitrogen atmosphere in screw-cap reaction tubes. All the solvents used for the reactions were dried by following the reported procedures [1]. Unless otherwise noted, all materials were purchased from commercial suppliers and used as received. All sulfonyl azides were prepared in-house using conventional procedures [2]. Reactions were monitored using thin-layer chromatography (SiO_2). A gradient elution using petroleum ether and ethyl acetate was performed based on Merck aluminium TLC sheets (silica gel 60 F_{254}). TLC plates were visualized with UV light (254 nm). For column chromatography, silica gel (100–200 mesh) from SRL Co. was used. NMR studies were performed on a Bruker Advance DPX at 400 MHz (^{1}H) or 500 MHz (^{1}H) and at 100 MHz (^{13}C) or 125 MHz (^{13}C), respectively. Chemical shifts (δ) are reported in ppm, using the residual solvent peak in $CDCl_3$ (δH = 7.26 and δC = 77.16) ppm as internal standards, and coupling constants (*J*) are given in Hz. HRMS were recorded with a Bruker MaXis impact mass spectrometer using ESI-TOF techniques. Melting points were measured with a Buchi Melting Point B-545 apparatus.

Experimental procedures

(a) General procedure for the synthesis of quinoline *N*-oxides [3]

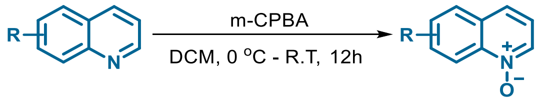

The quinoline *N*-oxides were prepared by following a reported procedure. To a 100 mL round-bottom flask equipped with a magnetic stir bar, substituted quinoline (10 mmol, 1.0 equiv) in CH_2Cl_2 was added *m*-CPBA (15 mmol, 1.5 equiv) portion wise over 1 h at 0 °C. The reaction mixture was allowed to stir for 12 h at rt. After completion, the reaction mixture was quenched with aqueous 1 M NaOH solution, extracted with DCM and sat. $NaHCO_3$. The combined organic layer was washed with brine and dried over Na_2SO_4, concentrated under reduced pressure, and purified by column chromatography (EA/petroleum ether 1:9) to get the desired product in up to 90% yield.

(b) General procedure for the synthesis of *N*-sulfonyl-1,2,3-triazoles [4]

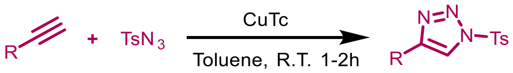

In a 50 mL round-bottom flask equipped with a magnetic stir bar, substituted terminal alkyne (0.5 mmol, 1.0 equiv), sulfonyl azide (0.5 mmol, 1.0 equiv) and copper(I) thiophene-2-carboxylate (5 mol %) in toluene (10 mL) were placed. The resulting mixture was stirred for 1–2 h at rt. After the completion of the reaction, this was quenched with saturated aq NH_4Cl solution and extracted with EtOAc. The combined organic phases were washed with brine, dried over Na_2SO_4, and concentrated under reduced pressure. The resulting residue was purified by recrystallization (DCM/hexane 1:9) to afford the desired product in up to 95% yield.

(c) General procedure of α-triazolyl quinoline

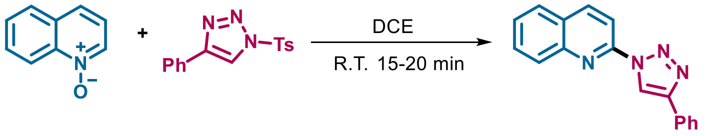

To an oven dried reaction tube equipped with a magnetic stirring bar was added Quinoline *N*-oxide **5** (0.2 mmol, 1.0 equiv), 4-phenyl-1-tosyl -1*H*-1,2,3-triazole **6a** (0.24 mmol, 1.2 equiv). The nitrogen was passed through the reaction mixture three times followed by addition of 1,2-dichloroethane (2 mL) *via* syringe. The reaction mixture was allowed to stir at R.T for 15-20 min. After completion of the reaction, solvent was evaporated under reduced pressure and the residue was purified by column chromatography (EA:Pet ether 1:9) to get the desired product **3a**in 92% yield. All the compounds are synthesized by following the same procedure.

Spectroscopic data of the compounds

3a

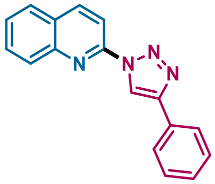

2-(4-Phenyl-1*H*-1,2,3-triazol-1-yl)quinoline

50 mg, 92 % yield, white solid

Melting Point: 150-152°C

^{1}H NMR (500 MHz, $CDCl_3$):δ 9.03 (s, 1H), 8.44 – 8.36 (m, 2H), 8.09 (d, J = 8.4 Hz, 1H), 8.01 (d, J = 7.5 Hz, 2H), 7.91 (d, J = 8.1 Hz, 1H), 7.80 (t, J = 7.6 Hz, 1H), 7.61 (t, J = 7.5 Hz, 1H), 7.49 (t, J = 7.5 Hz, 2H), 7.39 (t, J = 7.3 Hz, 1H).

^{13}C NMR (100 MHz, CDCl$_3$): δ 148.3, 148.0, 146.6, 139.8, 131.0, 130.4, 129.1, 129.0, 128.7, 128.1, 128.0, 127.2, 126.1, 117.0, 112.8.

HRMS (ESI): Calcd. for $C_{17}H_{13}N_4$ $[M+H]^+$273.1135, found 273.1133.

The spectroscopic data is consistent with a literature report [5].

3b

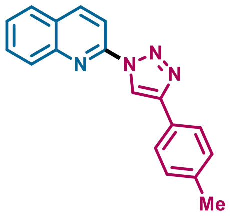

2-(4-(*p*-Tolyl)-1*H*-1,2,3-triazol-1-yl)quinoline

54 mg, 94 % yield, white solid

Melting Point: 154-156°C

^{1}H NMR (400 MHz, CDCl$_3$):δ 8.88 (s, 1H), 8.33 – 8.23 (m, 2H), 7.99 (d, J = 8.5 Hz, 1H), 7.83 – 7.78 (m, 3H), 7.70 (ddt, J = 8.4, 7.0, 1.4 Hz, 1H), 7.50 (ddt, J = 8.1, 7.0, 1.1 Hz, 1H), 7.22 (d, J = 7.8 Hz, 2H), 2.34 (s, 3H).

^{13}C NMR (100 MHz, CDCl$_3$): δ 148.0, 146.5, 139.6, 138.5, 130.8, 129.7, 128.9, 127.9, 127.5, 127.1, 126.0, 116.6, 112.7, 21.4.

HRMS (ESI): Calcd. for $C_{18}H_{15}N_4$ $[M+H]^+$ 287.1291, found 287.1297.

The spectroscopic data is consistent with a literature report [5].

3c

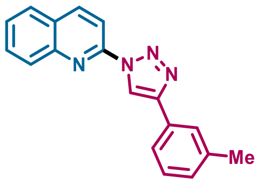

2-(4-(*m*-Tolyl)-1*H*-1,2,3-triazol-1-yl)quinoline

49 mg, 85 % yield, white solid.

Melting Point: 151-153°C

¹H NMR (400 MHz, CDCl₃):δ 8.97 (d, J = 0.7 Hz, 1H), 8.39 – 8.30 (m, 2H), 8.05 (d, J = 8.4 Hz, 1H), 7.89 – 7.82 (m, 2H), 7.80 – 7.73 (m, 2H), 7.57 (t, J = 7.5 Hz, 1H), 7.36 (t, J = 7.6 Hz, 1H), 7.19 (d, J = 7.6 Hz, 1H), 2.44 (s, 3H).

¹³C NMR (100 MHz, CDCl₃): δ 148.3, 147.9, 146.5, 139.6, 138.7, 130.8, 130.2, 129.4, 128.9, 128.9, 127.9, 127.1, 126.7, 123.2, 116.9, 112.7, 21.6.

HRMS (ESI): Calcd. for $C_{18}H_{15}N_4$ $[M+H]^+$ 287.1291, found 287.1291.

3d

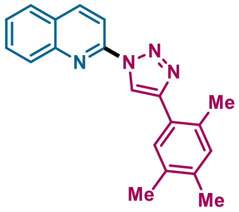

2-(4-(2,4,5-Trimethylphenyl)-1*H*-1,2,3-triazol-1-yl)quinoline

52 mg, 83 % yield, white solid

Melting Point: 145-147°C

¹H NMR (500 MHz, CDCl₃):δ 8.88 (s, 1H), 8.45 – 8.37 (m, 2H), 8.09 (d, J = 8.4 Hz, 1H), 7.90 (d, J = 8.0 Hz, 1H), 7.82 – 7.76 (m, 1H), 7.73 (s, 1H), 7.63 – 7.58 (m, 1H), 7.10 (s, 1H), 2.55 (s, 3H), 2.32 (s, 3H), 2.30 (s, 3H).

¹³C NMR (125 MHz, CDCl₃): δ 148.2, 146.7, 139.7, 137.1, 134.4, 133.1, 132.5, 130.9, 130.2, 129.0, 128.00, 127.98, 127.1, 127.0, 118.8, 112.9, 21.1, 19.6, 19.4.

HRMS (ESI): Calcd. for $C_{20}H_{19}N_4$ $[M+H]^+$ 315.1604, found 315.1607.

3e

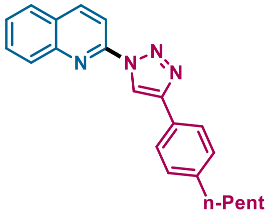

2-(4-(4-Pentylphenyl)-1*H*-1,2,3-triazol-1-yl)quinoline

62 mg, 90 % yield, white solid

Melting Point: 143-145°C

^{1}H NMR (400 MHz, CDCl$_3$): δ 8.97 (s, 1H), 8.43 – 8.30 (m, 2H), 8.06 (d, J = 7.9 Hz, 1H), 7.95 – 7.84 (m, 3H), 7.77 (t, J = 7.2 Hz, 1H), 7.57 (t, J = 7.4 Hz, 1H), 7.30 (d, J = 7.5 Hz, 2H), 2.66 (t, J = 7.6 Hz, 2H), 1.71 – 1.62 (m, 2H), 1.39 – 1.32 (m, 4H), 0.91 (t, J = 5.9 Hz, 3H).

^{13}C NMR (100 MHz, CDCl$_3$): δ 148.0, 146.6, 143.6, 139.6, 130.8, 129.1, 129.1, 128.9, 127.9, 127.7, 127.1, 126.1, 126.0, 116.6, 112.7, 35.9, 31.6, 31.2, 22.7, 14.2.

HRMS (ESI): Calcd. for $C_{22}H_{22}N_4Na$ $[M+Na]^+$ 365.1737, found 365.1736.

3f

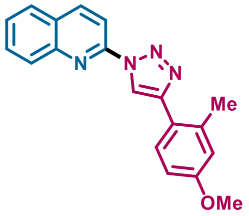

2-(4-(4-Methoxy-2-methylphenyl)-1*H*-1,2,3-triazol-1-yl)quinoline

55mg, 87 % yield, white solid

Melting Point: 152-154°C

^{1}H NMR (400 MHz, CDCl$_3$): δ 8.83 (s, 1H), 8.44 – 8.34 (m, 2H), 8.08 (d, J = 8.4 Hz, 1H), 7.89 (d, J = 8.1 Hz, 1H), 7.85 (d, J = 9.2 Hz, 1H), 7.78 (t, J = 7.3 Hz, 1H), 7.59 (t, J = 7.4 Hz, 1H), 6.91 – 6.82 (m, 2H), 3.86 (s, 3H), 2.59 (s, 3H).

^{13}C NMR (100 MHz, CDCl$_3$): δ 159.8, 148.1, 147.6, 146.6, 139.7, 137.7, 130.9, 130.5, 129.0, 128.0, 127.1, 122.4, 118.5, 116.5, 112.8, 111.6, 55.4, 21.9.

HRMS (ESI): Calcd. for $C_{19}H_{17}N_4O$ $[M+H]^+$ 317.1397, found 317.1399.

3g

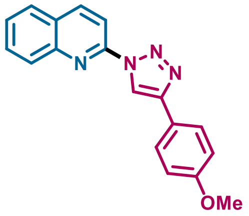

2-(4-(4-Methoxyphenyl)-1*H*-1,2,3-triazol-1-yl)quinoline

57 mg, 95 % yield, white solid

Melting Point: 149-151°C

^{1}H NMR (500 MHz, $CDCl_3$): δ 8.95 (s, 1H), 8.45 – 8.37 (m, 2H), 8.08 (d, J = 8.4 Hz, 1H), 7.96 – 7.88 (m, 3H), 7.80 (t, J = 7.7 Hz, 1H), 7.61 (t, J = 7.5 Hz, 1H), 7.02 (d, J = 8.5 Hz, 2H), 3.88 (s, 3H).

^{13}C NMR (125 MHz, $CDCl_3$): δ 160.1, 148.2, 148.1, 146.7, 139.7, 130.9, 129.0, 128.03, 128.01, 127.5, 127.2, 123.1, 116.2, 114.5, 112.9, 55.5.

HRMS (ESI): Calcd. for $C_{18}H_{15}N_4O$ $[M+H]^+$ 303.1240, found 303.1237.

3h

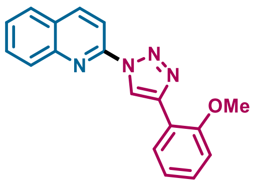

2-(4-(2-Methoxyphenyl)-1*H*-1,2,3-triazol-1-yl)quinoline

55mg, 91 % yield, white solid

Melting Point: 144-146°C

^{1}H NMR (400 MHz, $CDCl_3$): δ 9.18 (s, 1H), 8.46 (d, J = 7.3 Hz, 1H), 8.37 (dd, J = 27.2, 8.8 Hz, 2H), 8.11 (d, J = 8.4 Hz, 1H), 7.87 (d, J = 8.0 Hz, 1H), 7.77 (t, J = 7.5 Hz, 1H), 7.57 (t, J = 7.4 Hz, 1H), 7.36 (t, J = 7.4 Hz, 1H), 7.13 (t, J = 7.4 Hz, 1H), 7.03 (d, J = 8.2 Hz, 1H), 4.03 (s, 3H).

^{13}C NMR (100 MHz, $CDCl_3$): δ 156.2, 148.3, 146.6, 139.5, 130.7, 129.4, 129.0, 128.1, 127.9, 127.0, 121.1, 120.2, 119.2, 113.0, 111.0, 55.6.

HRMS (ESI): Calcd. for $C_{18}H_{15}N_4O$ $[M+H]^+$ 303.1240, found 303.1242.

3i

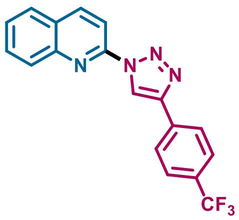

2-(4-(4-(Trifluoromethyl)phenyl)-1*H*-1,2,3-triazol-1-yl)quinoline

55 mg, 81 % yield, white solid

Melting Point: 195-197°C

^{1}H NMR (400 MHz, CDCl$_3$): δ 9.10 (s, 1H), 8.39 (s, 2H), 8.14 – 8.05 (m, 3H), 7.91 (d, J = 8.1 Hz, 1H), 7.81 (t, J = 7.6 Hz, 1H), 7.73 (d, J = 8.2 Hz, 2H), 7.62 (t, J = 7.5 Hz, 1H).

^{13}C NMR (100 MHz, CDCl$_3$): δ 147.8, 146.9, 146.6, 139.9, 133.8, 131.1, 129.0, 128.1, 128.0, 127.4, 126.9 (q = 271.8 Hz),126.2, 126.1 (q, J = 3.7 Hz), 117.9, 112.7.

HRMS (ESI): Calcd. for $C_{18}H_{11}F_3N_4Na$ $[M+Na]^+$ 363.0828, found 363.0822.

3j

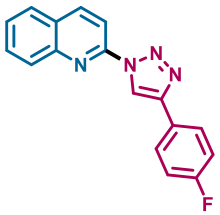

2-(4-(4-Fluorophenyl)-1*H*-1,2,3-triazol-1-yl)quinoline

49 mg, 84 % yield, white solid

Melting Point: 157-159 °C

^{1}H NMR (400 MHz, CDCl$_3$): δ 8.96 (s, 1H), 8.37 (s, 2H), 8.06 (d, J = 8.4 Hz, 1H), 7.99 – 7.92 (m, 2H), 7.88 (d, J = 8.0 Hz, 1H), 7.78 (t, J = 7.5 Hz, 1H), 7.59 (t, J = 7.4 Hz, 1H), 7.16 (t, J = 8.5 Hz, 2H).

^{13}C NMR (100 MHz, CDCl$_3$):δ 163.0 (d, J = 247.8 Hz), 147.9, 147.4, 146.6, 139.8, 131.0, 128.9, 128.0 (d, J = 4.3 Hz), 127.98, 127.9, 127.8, 127.2, 126.6 (d, J = 3.2 Hz),116.8, 116.1 (d, J = 21.8 Hz),112.7.

HRMS (ESI): Calcd. for $C_{17}H_{12}FN_4$ $[M+H]^+$ 291.1041, found 291.1043.

3k

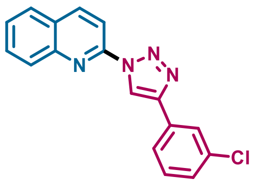

2-(4-(3-Chlorophenyl)-1*H*-1,2,3-triazol-1-yl)quinoline

52 mg, 85 % yield, white solid

Melting Point: 152-154 °C

^{1}H NMR (400 MHz, $CDCl_3$): δ 9.01 (s, 1H), 8.34 (s, 2H), 8.06 (d, J = 8.3 Hz, 1H), 7.98 (s, 1H), 7.87 (t, J = 8.4 Hz, 2H), 7.78 (t, J = 7.4 Hz, 1H), 7.59 (t, J = 7.5 Hz, 1H), 7.40 (t, J = 7.8 Hz, 1H), 7.34 (d, J = 7.9 Hz, 1H).

^{13}C NMR (125 MHz, $CDCl_3$): δ 147.8, 147.0, 146.5, 139.8, 135.0, 132.1, 131.0, 130.3, 128.9, 128.6, 128.03, 127.97, 127.3, 126.1, 124.1, 117.4, 112.6.

HRMS (ESI): Calcd. for $C_{17}H_{12}ClN_4[M+H]^+$ 307.0745, found 307.0743.

3l

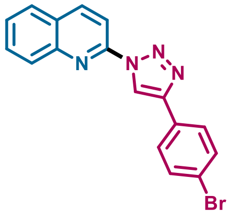

2-(4-(4-Bromophenyl)-1*H*-1,2,3-triazol-1-yl)quinoline

63 mg, 90 % yield, white solid

Melting Point: 142-144 °C

^{1}H NMR (400 MHz, $CDCl_3$): δ 9.03 (s, 1H), 8.40 (s, 2H), 8.08 (d, J = 8.4 Hz, 1H), 7.91 (d, J = 8.1 Hz, 1H), 7.87 (d, J = 8.5 Hz, 2H), 7.80 (ddt, J = 8.4, 7.0, 1.4 Hz, 1H), 7.64 – 7.59 (m, 3H).

^{13}C NMR (100 MHz, $CDCl_3$): δ 147.9, 147.3, 146.6, 139.9, 132.3, 131.0, 129.3, 129.0, 128.1, 128.0, 127.6, 127.3, 122.6, 117.2, 112.7.

HRMS (ESI): Calcd. for $C_{17}H_{12}BrN_4$ $[M+H]^+$ 351.0240, found 351.0244.

3m

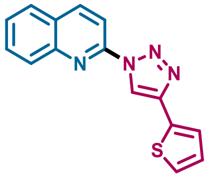

2-(4-(Thiophen-2-yl)-1*H*-1,2,3-triazol-1-yl)quinoline

46 mg, 82 % yield, white solid

Melting Point: 158-160 °C

^{1}H NMR (500 MHz, CDCl$_3$): δ 8.90 (s, 1H), 8.41 – 8.33 (m, 2H), 8.06 (d, J = 8.4 Hz, 1H), 7.89 (d, J = 8.1 Hz, 1H), 7.85 (d, J = 1.9 Hz, 1H), 7.78 (t, J = 7.7 Hz, 1H), 7.62 – 7.56 (m, 2H), 7.44 (dd, J = 4.8, 3.0 Hz, 1H).

^{13}C NMR (125 MHz, CDCl$_3$): δ 148.0, 146.6, 144.5, 139.7, 131.6, 130.9, 128.9, 128.01, 127.98, 127.2, 126.6, 126.1, 122.0, 116.7, 112.8.

HRMS (ESI): Calcd. for $C_{15}H_{11}N_4S$ $[M+H]^+$279.0699 , found 279.0671.

3n

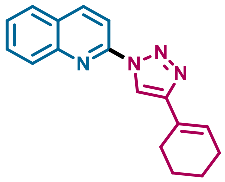

2-(4-(Cyclohex-1-en-1-yl)-1*H*-1,2,3-triazol-1-yl)quinoline

42mg, 75 % yield, white semi-solid

Melting Point: 128-130 °C

^{1}H NMR (400 MHz, CDCl$_3$): δ 8.61 (s, 1H), 8.35 – 8.33 (m, 2H), 8.03 (d, J = 8.5 Hz, 1H), 7.87 (d, J = 8.1 Hz, 1H), 7.78 – 7.74 (m, 1H), 7.57 (t, J = 7.0 Hz, 1H), 6.75 – 6.72 (m, 1H), 2.51 – 2.47 (m, 2H), 2.28 – 2.24 (m, 2H), 1.84 – 1.80 (m, 2H), 1.73 – 1.69 (m, 2H).

^{13}C NMR (100 MHz, CDCl$_3$): δ 149.9, 148.2, 146.6, 139.6, 130.8, 128.9, 127.94, 127.89, 127.02, 127.00, 126.3, 115.6, 112.8, 26.5, 25.5, 22.6, 22.3.

HRMS (ESI): Calcd. for $C_{17}H_{17}N_4$ $[M+H]^+$277.1448, found 277.1455.

The spectroscopic data is consistent with a literature report [5].

3o

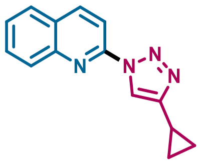

2-(4-Cyclopropyl-1*H*-1,2,3-triazol-1-yl)quinoline

36 mg, 77 % yield, white solid

Melting Point: 83-85°C

^{1}H NMR (500 MHz, CDCl$_3$): δ 8.46 (s, 1H), 8.33 (s, 2H), 8.02 (d, J = 8.5 Hz, 1H), 7.87 (d, J = 8.1 Hz, 1H), 7.76 (t, J = 7.7 Hz, 1H), 7.57 (t, J = 7.5 Hz, 1H), 2.13 – 2.04 (m, 1H), 1.07 – 1.01 (m, 2H), 0.98 – 0.95 (m, 2H).

^{13}C NMR (125 MHz, CDCl$_3$): δ 151.2, 148.1, 146.6, 139.6, 130.8, 128.9, 127.93, 127.87, 127.0, 117.1, 112.8, 8.1, 6.9.

HRMS (ESI): Calcd. for $C_{14}H_{13}N_4$ $[M+H]^+$ 237.1135, found 237.1136.

3p

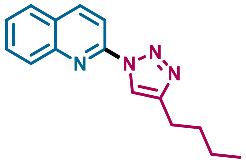

2-(4-Butyl-1*H*-1,2,3-triazol-1-yl)quinoline

37 mg, 73 % yield, colorless oil

^{1}H NMR (400 MHz, CDCl$_3$): δ 8.53 (s, 1H), 8.33 (s, 2H), 8.03 (d, J = 8.5 Hz, 1H), 7.86 (d, J = 8.1 Hz, 1H), 7.78 – 7.73 (m, 1H), 7.57 (t, J = 7.5 Hz, 1H), 2.84 (t, J = 7.7 Hz, 2H), 1.81 – 1.72 (m, 2H), 1.50 – 1.41 (m, 2H), 0.97 (t, J = 7.4 Hz, 3H).

^{13}C NMR (100 MHz, CDCl$_3$): δ 149.2, 148.2, 146.6, 139.6, 130.8, 128.9, 127.92, 127.88, 127.0, 118.3, 112.8, 31.5, 25.5, 22.4, 14.0.

HRMS (ESI): Calcd. for $C_{15}H_{17}N_4[M+H]^+$ 253.1448, found 253.1446.

3q

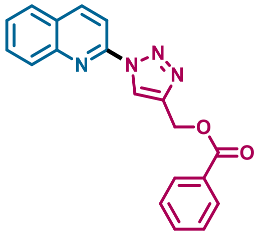

(1-(Quinolin-2-yl)-1*H*-1,2,3-triazol-4-yl)methyl benzoate

53 mg, 80 % yield, white solid

Melting Point: 149-151°C

^{1}H NMR (400 MHz, CDCl$_3$): δ 8.93 (s, 1H), 8.39 – 8.34 (m, 2H), 8.10 (d, J = 7.7 Hz, 2H), 8.06 (d, J = 8.5 Hz, 1H), 7.89 (d, J = 8.1 Hz, 1H), 7.79 (t, J = 7.7 Hz, 1H), 7.62 – 7.54 (m, 2H), 7.44 (t, J = 7.7 Hz, 2H), 5.61 (s, 2H).

^{13}C NMR (100 MHz, CDCl$_3$): δ 166.6, 147.8, 146.6, 143.7, 139.9, 133.4, 131.0, 130.0, 129.9, 129.1, 128.6, 128.1, 128.0, 127.3, 121.7, 112.7, 58.2.

HRMS (ESI): Calcd. for $C_{19}H_{14}N_4NaO_2$[M+Na]$^+$ 353.1009, found 353.1006

3r

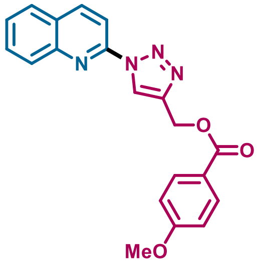

(1-(Quinolin-2-yl)-1*H*-1,2,3-triazol-4-yl)methyl 4-methoxybenzoate

60 mg, 83 % yield, white solid

Melting Point: 140-142°C

^{1}H NMR (500 MHz, CD-Cl$_3$): δ 8.92 (s, 1H), 8.39 – 8.34 (m, 2H), 8.07 – 8.02 (m, 3H), 7.89 (d, J = 8.0 Hz, 1H), 7.81 – 7.76 (m, 1H), 7.60 (t, J = 7.5 Hz, 1H), 6.91 (d, J = 8.9 Hz, 2H), 5.58 (s, 2H), 3.85 (s, 3H).

^{13}C NMR (125 MHz, CDCl$_3$): δ 166.3, 163.7, 147.8, 146.5, 143.9, 139.8, 132.0, 131.0, 129.0, 128.1, 128.0, 127.3, 122.2, 121.6, 113.8, 112.7, 57.9, 55.6.

HRMS (ESI): Calcd. for $C_{20}H_{17}N_4O_3$ [M+H]$^+$ 361.1295, found 361.1299

3s

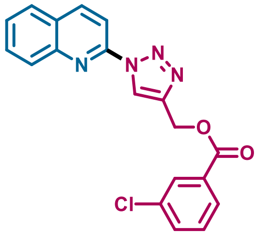

(1-(Quinolin-2-yl)-1*H*-1,2,3-triazol-4-yl)methyl 3-chlorobenzoate

58 mg, 79 % yield, white solid

Melting Point: 139-141°C

1H NMR (500 MHz, $CDCl_3$): δ 8.93 (s, 1H), 8.39 – 8.34 (m, 2H), 8.07 – 8.03 (m, 2H), 7.97 (d, J = 7.8 Hz, 1H), 7.89 (d, J = 8.1 Hz, 1H), 7.80 – 7.76 (m, 1H), 7.62 – 7.58 (m, 1H), 7.53 (dd, J = 8.0, 1.0 Hz, 1H), 7.38 (t, J = 7.9 Hz, 1H), 5.61 (s, 2H).

^{13}C NMR (125 MHz, $CDCl_3$): δ 165.4, 147.8, 146.5, 143.7, 139.9, 134.7, 133.4, 131.5, 131.0, 130.0, 129.9, 129.0, 128.1, 128.0, 127.4, 121.8, 112.7, 58.5.

HRMS (ESI): Calcd. for $C_{19}H_{13}ClKN_4O_2$[M+K]$^+$403.0568, found 403.0568

3t

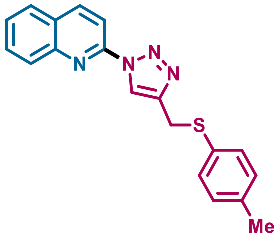

2-(4-((*p*-Tolylthio)methyl)-1*H*-1,2,3-triazol-1-yl)quinoline

50 mg, 75 % yield, white solid

Melting Point: 127-129°C

^{1}H NMR (400 MHz, $CDCl_3$): δ 8.61 (s, 1H), 8.36 – 8.29 (m, 2H), 8.02 (d, J = 8.5 Hz, 1H), 7.87 (d, J = 8.1 Hz, 1H), 7.79 – 7.74 (m, 1H), 7.61 – 7.55 (m, 1H), 7.32 (d, J = 8.1 Hz, 2H), 7.10 (d, J = 8.0 Hz, 2H), 4.31 (s, 2H), 2.30 (s, 3H).

^{13}C NMR (100 MHz, $CDCl_3$): δ 147.9, 146.5, 146.0, 139.7, 137.0, 131.8, 130.9, 130.5, 130.0, 129.0, 128.0, 127.9, 127.2, 119.8, 112.7, 29.8, 21.2.

HRMS (ESI): Calcd. for $C_{19}H_{16}N_4NaS$ [M+Na]$^+$ 355.0988, found 355.0985

3u

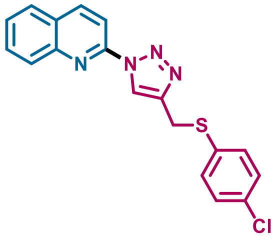

2-(4-(((4-Chlorophenyl)thio)methyl)-1*H*-1,2,3-triazol-1-yl)quinoline

55 mg, 78 % yield, white solid

Melting Point: 135-137°C

^{1}H NMR (400 MHz, CDCl$_3$): δ 8.63 (s, 1H), 8.37 – 8.29 (m, 2H), 8.02 (d, J = 8.5 Hz, 1H), 7.87 (d, J = 8.1 Hz, 1H), 7.77 (t, J = 7.6 Hz, 1H), 7.58 (t, J = 7.5 Hz, 1H), 7.33 (d, J = 8.5 Hz, 2H), 7.25 (d, J = 8.4 Hz, 2H), 4.32 (s, 2H).

^{13}C NMR (100 MHz, CDCl$_3$): δ 147.8, 146.5, 145.5, 139.8, 134.0, 132.8, 131.2, 130.9, 129.3, 129.0, 128.0, 127.9, 127.2, 119.8, 112.6, 29.3.

HRMS (ESI): Calcd. for $C_{18}H_{14}ClN_4S$ $[M+H]^+$ 353.0622, found 353.0627

3v

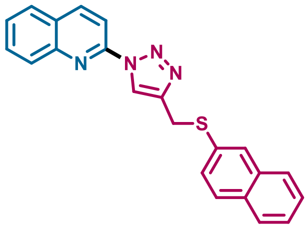

2-(4-((Naphthalen-2-ylthio)methyl)-1*H*-1,2,3-triazol-1-yl)quinoline

49 mg, 67 % yield, white solid

Melting Point: 151-153°C

^{1}H NMR (500 MHz, CDCl$_3$): δ 8.67 (s, 1H), 8.35 – 8.29 (m, 2H), 7.98 (d, J = 8.5 Hz, 1H), 7.88 – 7.84 (m, 2H), 7.79 – 7.73 (m, 4H), 7.57 (t, J = 7.5 Hz, 1H), 7.50 (d, J = 8.6 Hz, 1H), 7.47 – 7.41 (m, 2H), 4.46 (s, 2H).

^{13}C NMR (125 MHz, CDCl$_3$): δ 147.9, 146.5, 145.7, 139.7, 133.9, 133.1, 132.1, 130.9, 129.0, 128.8, 128.0, 127.91, 127.8, 127.6, 127.41, 127.39, 127.2, 126.7, 126.0, 119.9, 112.7, 29.0.

HRMS (ESI): Calcd. for $C_{22}H_{17}N_4S$ $[M+H]^+$ 369.1168, found 369.1164

3w

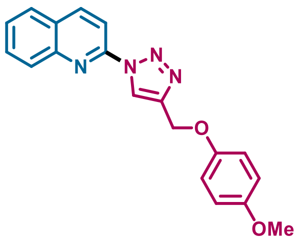

2-(4-((4-Methoxyphenoxy)methyl)-1*H*-1,2,3-triazol-1-yl)quinoline

47 mg, 71 % yield, white solid

Melting Point: 137-139°C

^{1}H NMR (500 MHz, $CDCl_3$): δ 8.85 (s, 1H), 8.36 (s, 2H), 8.04 (d, J = 8.5 Hz, 1H), 7.88 (d, J = 8.0 Hz, 1H), 7.77 (t, J = 7.6 Hz, 1H), 7.59 (t, J = 7.4 Hz, 1H), 6.99 (d, J = 8.8 Hz, 2H), 6.86 (d, J = 8.7 Hz, 2H), 5.29 (s, 2H), 3.77 (s, 3H).

^{13}C NMR (125 MHz, $CDCl_3$): δ 154.4, 152.5, 147.9, 146.5, 145.2, 139.8, 130.9, 129.0, 128.0, 127.9, 127.3, 120.5, 116.0, 114.8, 112.7, 62.8, 55.8.

HRMS (ESI): Calcd. for $C_{19}H_{16}N_4NaO_2$[M+Na]$^+$355.1165, found 355.1164.

3x

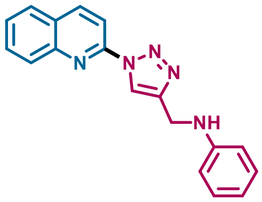

***N*-((1-(Quinolin-2-yl)-1*H*-1,2,3-triazol-4-yl)methyl)aniline**

45 mg, 75 % yield, white solid

Melting Point: 147-149°C

^{1}H NMR (400 MHz, $CDCl_3$): δ 8.70 (s, 1H), 8.33 (s, 2H), 8.01 (d, J = 8.5 Hz, 1H), 7.87 (d, J = 8.1 Hz, 1H), 7.79 – 7.73 (m, 1H), 7.58 (t, J = 7.4 Hz, 1H), 7.22 (t, J = 7.9 Hz, 2H), 6.81 – 6.70 (m, 3H), 4.59 (s, 2H), 4.39 (d, J = 23.9 Hz, 1H).

^{13}C NMR (100 MHz, $CDCl_3$): δ 147.9, 147.6, 147.0, 146.5, 139.7, 130.9, 129.5, 129.0, 128.0, 127.9, 127.2, 119.3, 118.2, 113.3, 112.7, 40.1.

HRMS (ESI): Calcd. for $C_{18}H_{16}N_5$ [M+H]$^+$ 302.1400, found 302.1395

3y

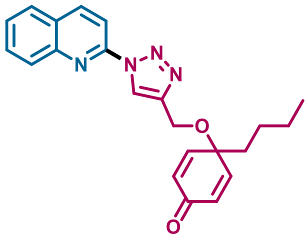

4-Butyl-4-((1-(quinolin-2-yl)-1*H*-1,2,3-triazol-4-yl)methoxy)cyclohexa-2,5-dien-1-one

49 mg, 66 % yield, white solid

Melting Point: 119-121°C

^{1}H NMR (400 MHz, $CDCl_3$): δ 8.76 (s, 1H), 8.39 – 8.34 (m, 2H), 8.05 (d, J = 8.4 Hz, 1H), 7.90 (d, J = 8.0 Hz, 1H), 7.81 – 7.76 (m, 1H), 7.60 (t, J = 7.5 Hz, 1H), 6.90 (d, J = 10.2 Hz, 2H), 6.43 (d, J = 10.2 Hz, 2H), 4.63 (s, 2H), 1.85 – 1.80 (m, 2H), 1.31 – 1.24 (m, 4H), 0.86 (d, J = 7.1 Hz, 3H).

^{13}C NMR (125 MHz, $CDCl_3$): δ 185.6, 150.9, 147.9, 146.6, 145.9, 139.8, 131.7, 131.0, 129.0, 128.04, 127.99, 127.3, 120.3, 112.7, 76.5, 59.4, 39.3, 25.7, 23.0, 14.0.

HRMS (ESI): Calcd. for $C_{22}H_{22}KN_4O_2$[M+K]$^{+}$413.1374, found 413.1367.

3z

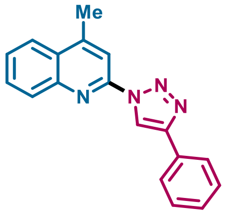

4-Methyl-2-(4-phenyl-1*H*-1,2,3-triazol-1-yl)quinoline

49 mg, 85 % yield, white solid

Melting Point: 156-158°C

^{1}H NMR (400 MHz, $CDCl_3$): δ 9.01 (s, 1H), 8.26 (s, 1H), 8.06 (dd, J = 12.4, 8.4 Hz, 2H), 8.00 (d, J = 7.3 Hz, 2H), 7.77 (t, J = 7.2 Hz, 1H), 7.61 (t, J = 7.5 Hz, 1H), 7.48 (t, J = 7.6 Hz, 2H), 7.39 (t, J = 7.4 Hz, 1H), 2.83 (s, 3H).

^{13}C NMR (100 MHz, $CDCl_3$): δ 148.7, 147.8, 146.5, 130.6, 130.4, 129.5, 129.1, 128.6, 128.1, 126.9, 126.1, 124.2, 117.1, 113.0, 19.3.

HRMS (ESI): Calcd. for $C_{18}H_{15}N_4$ [M+H]$^{+}$ 287.1291, found 287.1295.

The spectroscopic data is consistent with a literature report [5].

3aa

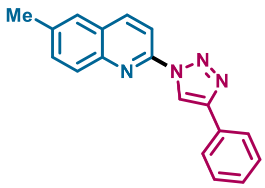

6-Methyl-2-(4-phenyl-1*H*-1,2,3-triazol-1-yl)quinoline

50 mg, 88 % yield, white solid

Melting Point: 151-153°C

^{1}H NMR (500 MHz, $CDCl_3$): δ 9.00 (s, 1H), 8.36 (d, J = 8.8 Hz, 1H), 8.28 (d, J = 8.8 Hz, 1H), 8.01 – 7.96 (m, 3H), 7.66 (s, 1H), 7.62 (dd, J = 8.6, 1.8 Hz, 1H), 7.49 (t, J = 7.6 Hz, 2H), 7.41 – 7.37 (m, 1H), 2.57 (s, 3H).

^{13}C NMR (125 MHz, $CDCl_3$): δ 148.3, 147.5, 145.2, 139.1, 137.4, 133.3, 130.5, 129.2, 128.7, 128.2, 127.0, 126.2, 117.1, 112.9, 21.8.

HRMS (ESI): Calcd. for $C_{18}H_{15}N_4$ $[M+H]^+$ 287.1291, found 287.1290.

3ab

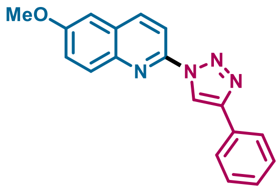

6-Methoxy-2-(4-phenyl-1*H*-1,2,3-triazol-1-yl)quinoline

54 mg, 90 % yield, white solid

Melting Point: 146-148°C

^{1}H NMR (500 MHz, $CDCl_3$): δ 8.94 (s, 1H), 8.32 (d, J = 8.8 Hz, 1H), 8.23 (d, J = 8.8 Hz, 1H), 7.98 (d, J = 7.2 Hz, 2H), 7.95 (d, J = 9.2 Hz, 1H), 7.47 (t, J = 7.6 Hz, 2H), 7.42 (dd, J = 9.2, 2.7 Hz, 1H), 7.38 (t, J = 7.4 Hz, 1H), 7.12 (d, J = 2.7 Hz, 1H), 3.93 (s, 3H).

^{13}C NMR (125 MHz, $CDCl_3$): δ 158.4, 148.1, 146.2, 142.2, 138.3, 130.4, 130.2, 129.1, 129.0, 128.6, 126.1, 123.6, 116.8, 112.9, 105.6, 55.8.

HRMS (ESI): Calcd. for $C_{18}H_{15}N_4O$ $[M+H]^+$ 303.1240, found 303.1243.

3ac

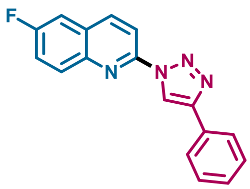

6-Fluoro-2-(4-phenyl-1*H*-1,2,3-triazol-1-yl)quinoline

47 mg, 81 % yield, white solid

Melting Point: 142-144°C

^{1}H NMR (400 MHz, CDCl$_3$): δ 8.92 (s, 1H), 8.38 (d, J = 8.8 Hz, 1H), 8.27 (d, J = 8.8 Hz, 1H), 8.06 – 8.00 (m, 1H), 7.96 (d, J = 7.4 Hz, 2H), 7.55 – 7.44 (m, 4H), 7.37 (t, J = 7.4 Hz, 1H).

^{13}C NMR (100 MHz, CDCl$_3$):δ 160.8 (d, J = 249.4 Hz), 148.3, 147.5, 143.4, 138.9 (d, J = 5.3 Hz), 131.3 (d, J = 9.2 Hz), 130.2, 129.0, 128.6, 128.5, 126.0, 121.0 (d, J = 25.7 Hz), 116.8, 113.5, 111.2 (d, J = 22.1 Hz).

HRMS (ESI): Calcd. for $C_{17}H_{12}FN_4$ $[M+H]^+$ 291.1041, found 291.1041.

3ad

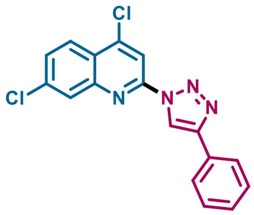

4,7-Dichloro-2-(4-phenyl-1*H*-1,2,3-triazol-1-yl)quinoline

53 mg, 77 % yield, white solid

Melting Point: 129-131°C

^{1}H NMR (400 MHz, CDCl$_3$): δ 8.44 (s, 1H), 8.27 (d, J = 1.9 Hz, 1H), 8.24 (s, 1H), 8.18 (d, J = 8.9 Hz, 1H), 7.97 (d, J = 7.0 Hz, 2H), 7.60 (dd, J = 8.9, 2.0 Hz, 1H), 7.50 (t, J = 7.3 Hz, 2H), 7.44 (t, J = 7.3 Hz, 1H).

^{13}C NMR (100 MHz, CDCl$_3$): δ 150.9, 149.7, 147.9, 145.2, 137.9, 135.1, 130.3, 129.8, 129.2, 128.9, 128.8, 126.7, 125.7, 124.3, 113.4.

HRMS (ESI): Calcd. for $C_{17}H_{10}Cl_2N_4Na$ $[M+Na]^+$363.0175, found 363.0182.

3ae

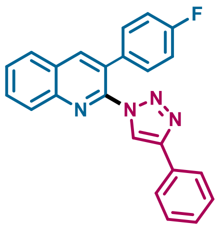

3-(4-Fluorophenyl)-2-(4-phenyl-1*H*-1,2,3-triazol-1-yl)quinoline

51 mg, 70 % yield, white solid

Melting Point: 153-155ºC

^{1}H NMR (400 MHz, $CDCl_3$): δ 8.35 (s, 2H), 8.17 (d, J = 8.5 Hz, 1H), 7.96 (d, J = 8.1 Hz, 1H), 7.88 (d, J = 7.3 Hz, 2H), 7.83 (d, J = 7.2 Hz, 1H), 7.70 (t, J = 8.1 Hz, 1H), 7.44 (t, J = 7.6 Hz, 2H), 7.35 (t, J = 7.4 Hz, 1H), 7.26 – 7.20 (m, 2H), 7.05 (t, J = 8.6 Hz, 2H).

^{13}C NMR (100 MHz, CDCl3): δ 162.8 (d, J = 248.2 Hz), 147.4, 146.2, 146.1, 140.8, 131.2, 130.5 (d, J = 8.3 Hz), 130.2, 129.5, 129.3, 129.0, 128.5 (d, J = 9.2 Hz), 128.3, 127.8, 126.4, 126.0, 120.3, 115.6 (d, J = 21.8 Hz).

HRMS (ESI): Calcd. for $C_{23}H_{15}FN_4Na$ $[M+Na]^+$ 389.1173, found 389.1175

3af

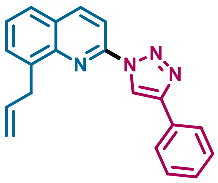

8-Allyl-2-(4-phenyl-1*H*-1,2,3-triazol-1-yl)quinoline

53 mg, 84 % yield, colorless oil

^{1}H NMR (500 MHz, $CDCl_3$):δ 8.97 (s, 1H), 8.43 – 8.36 (m, 2H), 8.01 (d, J = 7.2 Hz, 2H), 7.78 (d, J = 8.1 Hz, 1H), 7.66 (d, J = 6.9 Hz, 1H), 7.56 – 7.52 (m, 1H), 7.50 (t, J = 7.7 Hz, 2H), 7.40 (t, J = 7.4 Hz, 1H), 6.25 – 6.15 (m, 1H), 5.23 (dd, J = 17.1, 1.6 Hz, 1H), 5.16 (dd, J = 10.0, 1.5 Hz, 1H), 4.09 (d, J = 6.7 Hz, 2H).

^{13}C NMR (125 MHz, $CDCl_3$): δ 148.3, 147.1, 144.8, 140.2, 138.9, 137.4, 130.5, 130.4, 129.1, 128.7, 128.2, 127.1, 126.4, 126.2, 116.9, 116.3, 112.5, 35.7.

HRMS (ESI): Calcd. for $C_{20}H_{16}N_4Na$ $[M+Na]^+$ 335.1267, found 335.1272.

4a

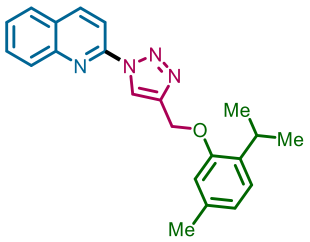

2-(4-((2-Isopropyl-5-methylphenoxy)methyl)-1*H*-1,2,3-triazol-1-yl)quinoline

50 mg, 70 % yield, colorless oil

^{1}H NMR (500 MHz, CDCl$_3$): δ 8.84 (s, 1H), 8.38 (s, 2H), 8.08 (d, J = 8.4 Hz, 1H), 7.90 (d, J = 8.1 Hz, 1H), 7.79 (t, J = 7.2 Hz, 1H), 7.61 (t, J = 7.4 Hz, 1H), 7.14 (d, J = 7.6 Hz, 1H), 6.87 (s, 1H), 6.80 (d, J = 7.9 Hz, 1H), 5.34 (s, 2H), 3.40 – 3.33 (m, 1H), 2.35 (s, 3H), 1.24 (d, J = 6.9 Hz, 6H).

^{13}C NMR (125 MHz, CDCl$_3$): δ 155.5, 148.0, 146.6, 139.8, 136.6, 134.6, 131.0, 129.1, 128.1, 128.0, 127.3, 126.2, 122.3, 122.0, 112.9, 112.8, 62.5, 26.7, 23.0, 21.5.

HRMS (ESI): Calcd. for $C_{22}H_{22}N_4NaO$ $[M+Na]^+$381.1686, found 381.1690.

4b

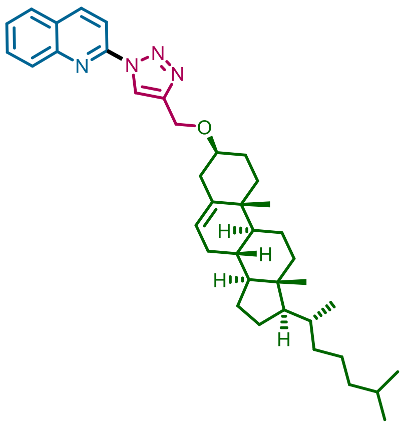

2-(4-((((3*S*,8*S*,9*S*,10*R*,13*R*,14*S*,17*R*)-10,13-Dimethyl-17-((*R*)-6-methylheptan-2-yl)-2,3,4,7,8,9,10,11,12,13,14,15,16,17-tetradecahydro-1*H*-cyclopenta[*a*]phenanthren-3-yl)oxy)methyl)-1*H*-1,2,3-triazol-1-yl)quinoline

81 mg, 68 % yield, colorless oil

^{1}H NMR (400 MHz, CDCl$_3$): δ 8.79 (s, 1H), 8.36 (s, 2H), 8.06 (d, J = 8.4 Hz, 1H), 7.89 (d, J = 7.3 Hz, 1H), 7.78 (ddd, J = 8.4, 7.0, 1.4 Hz, 1H), 7.62 – 7.57 (m, 1H), 5.40 – 5.36 (m, 1H), 4.82 (s, 2H), 3.44 – 3.35 (m, 1H), 2.50 – 2.43 (m, 1H), 2.34 – 2.27 (m, 1H), 2.04 – 1.95 (m, 3H), 1.92 – 1.79 (m, 2H), 1.58 – 1.42 (m, 7H), 1.41 – 1.20 (m, 5H), 1.19 – 1.05 (m, 7H), 1.02 (s, 3H), 1.00

– 0.94 (m, 2H), 0.91 (d, J = 6.5 Hz, 3H), 0.87 (d, J = 1.7 Hz, 3H), 0.85 (d, J = 1.7 Hz, 3H), 0.68 (s, 3H).

^{13}C NMR (125 MHz, CDCl$_3$): δ 148.1, 146.7, 146.6, 140.9, 139.7, 130.9, 129.1, 128.0, 128.0, 127.2, 122.0, 120.2, 112.8, 79.1, 61.6, 56.9, 56.3, 50.3, 42.5, 39.9, 39.7, 39.2, 37.4, 37.0, 36.3, 35.9, 32.10, 32.05, 28.5, 28.4, 28.2, 24.4, 24.0, 23.0, 22.7, 21.2, 19.5, 18.9, 12.0.

HRMS (ESI): Calcd. for $C_{39}H_{55}N_4O$ $[M+H]^+$595.4370, found 595.4363.

4c

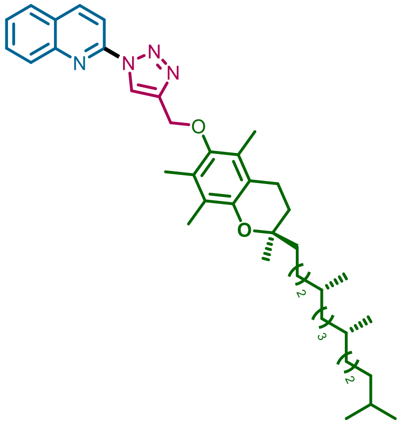

2-(4-((((*R*)-2,5,7,8-Tetramethyl-2-((4*R*,8*R*)-4,8,12-trimethyltridecyl)chroman-6-yl)oxy)methyl)-1*H*-1,2,3-triazol-1-yl)quinoline

78 mg, 61 % yield, colorless oil

^{1}H NMR (400 MHz, CDCl$_3$):δ 8.93 (s, 1H), 8.40 (s, 2H), 8.08 (d, J = 8.4 Hz, 1H), 7.91 (d, J = 8.2 Hz, 1H), 7.82 – 7.76 (m, 1H), 7.61 (dd, J = 11.1, 4.0 Hz, 1H), 4.97 (s, 2H), 2.61 (t, J = 6.7 Hz, 2H), 2.28 (s, 3H), 2.23 (s, 3H), 2.12 (s, 3H), 1.86 – 1.76 (m, 2H), 1.56 – 1.48 (m, 3H), 1.42 – 1.36 (m, 3H), 1.29 – 1.20 (m, 11H), 1.16 – 1.04 (m, 7H), 0.88 – 0.85 (m, J = 6.9 Hz, 12H).

^{13}C NMR (100 MHz, CDCl$_3$): δ 148.2, 147.9, 146.5, 145.6, 139.7, 130.8, 128.9, 127.94, 127.93, 127.85, 127.1, 126.0, 123.1, 120.1, 117.7, 112.7, 74.9, 66.4, 40.1, 39.4, 37.4, 32.8, 32.8, 32.73, 32.71, 28.0, 24.83, 24.82, 24.5, 23.9, 22.7, 22.6, 21.1, 20.7, 19.8, 19.7, 14.1, 13.0, 12.1, 11.9.

HRMS (ESI): Calcd. for $C_{41}H_{59}N_4O_2$ $[M+H]^+$639.4633, found 639.4637.

6a

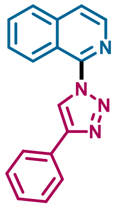

1-(4-Phenyl-1*H*-1,2,3-triazol-1-yl)isoquinoline

44 mg, 80 % yield, white solid

^{1}H NMR (400 MHz, CDCl$_3$): δ 8.88 (d, J = 8.7 Hz, 1H), 8.69 (s, 1H), 8.41 (d, J = 5.6 Hz, 1H), 7.96 (d, J = 7.4 Hz, 2H), 7.91 (d, J = 8.7 Hz, 1H), 7.78 – 7.74 (m, 2H), 7.69 (t, J = 7.7 Hz, 1H), 7.45 (t, J = 7.6 Hz, 2H), 7.35 (t, J = 7.0 Hz, 1H).

^{13}C NMR (100 MHz, CDCl$_3$): δ 152.1, 147.3, 140.5, 139.0, 131.4, 130.3, 129.2, 129.1, 128.6, 127.1, 126.6, 126.1, 122.7, 122.0, 120.7.

HRMS (ESI): Calcd. for $C_{17}H_{13}N_4$ $[M+H]^+$273.1135, found 273.1137.

6b

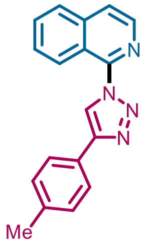

1-(4-(*p*-Tolyl)-1*H*-1,2,3-triazol-1-yl)isoquinoline

48 mg, 83 % yield, white solid

^{1}H NMR (500 MHz, CDCl$_3$): δ 8.93 (d, J = 8.6 Hz, 1H), 8.69 (s, 1H), 8.45 (d, J = 4.0 Hz, 1H), 7.94 (d, J = 8.2 Hz, 1H), 7.88 (d, J = 8.0 Hz, 2H), 7.82 – 7.78 (m, 2H), 7.73 (t, J = 7.5 Hz, 1H), 7.30 (d, J = 7.8 Hz, 2H), 2.41 (s, 3H).

^{13}C NMR (125 MHz, CDCl$_3$): δ 147.3, 140.5, 139.0, 138.5, 131.4, 129.8, 129.7, 129.1, 127.5, 127.0, 126.7, 126.0, 125.9, 122.6, 120.3, 21.4.

HRMS (ESI): Calcd. for $C_{18}H_{15}N_4$ $[M+H]^+$ 287.1291, found 287.1293.

6c

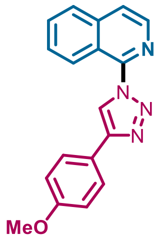

1-(4-(4-Methoxyphenyl)-1*H*-1,2,3-triazol-1-yl)isoquinoline

54 mg, 89 % yield, white solid

^{1}H NMR (400 MHz, CDCl$_3$): δ 8.92 (d, J = 8.6 Hz, 1H), 8.63 (s, 1H), 8.44 (d, J = 5.6 Hz, 1H), 7.94 – 7.88 (m, 3H), 7.81 – 7.76 (m, 2H), 7.72 (t, J = 7.7 Hz, 1H), 7.01 (d, J = 8.7 Hz, 2H), 3.86 (s, 3H).

^{13}C NMR (100 MHz, CDCl$_3$): δ 159.9, 152.0, 147.2, 140.5, 139.0, 131.3, 129.1, 127.4, 127.0, 126.7, 122.9, 122.5, 122.0, 119.8, 114.5, 55.5.

HRMS (ESI): Calcd. for $C_{18}H_{15}N_4O$ $[M+H]^+$ 303.1240, found 303.1241.

6d

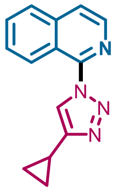

1-(4-Cyclopropyl-1*H*-1,2,3-triazol-1-yl)isoquinoline

34 mg, 71 % yield, colorless liquid

^{1}H NMR (400 MHz, CDCl$_3$): δ 8.82 (d, J = 8.6 Hz, 1H), 8.40 (d, J = 5.6 Hz, 1H), 8.16 (s, 1H), 7.92 (d, J = 8.2 Hz, 1H), 7.80 – 7.73 (m, 2H), 7.71 – 7.66 (m, 1H), 2.15 – 2.08 (m, 1H), 1.07 – 1.02 (m, 2H), 1.01 – 0.96 (m, 2H).

^{13}C NMR (100 MHz, CDCl$_3$): δ 150.1, 147.4, 140.4, 138.9, 131.3, 129.0, 127.0, 126.7, 122.4, 122.0, 120.8, 8.0, 6.8.

HRMS (ESI): Calcd. for $C_{14}H_{13}N_4$ $[M+H]^+$ 237.1135, found 237.1137.

References

1. Bradley, D.; Williams, G.; Lawton, M. *J. Org. Chem.* **2010**, *75*, 8351–8354.
2. Serwinski, P. R.; Esat, B.; Lahti, P. M.; Liao,Y.; Walton, R.; Lan, J. *J. Org. Chem.* **2004**, *69*, 5247-5260.
3. Neelakantan, H.; Wang, H.; Vance, V.; Hommel, J. D.; McHardy, S. F.; Watowich, S. J. *J. Med. Chem.* **2017**, *60*, 5015−5028.
4. Raushel, J.; Fokin, V. V. *Org. Lett.*, **2010**, *12*, 4952–4955.
5. Chattopadhyay, B.; Vera, C. I. R.; Chuprakov, S.; Gevorgyan, V. *Org. Lett.* **2010**, *12*, 2166–2169.

^{1}H and ^{13}C NMR Spectra of 3a

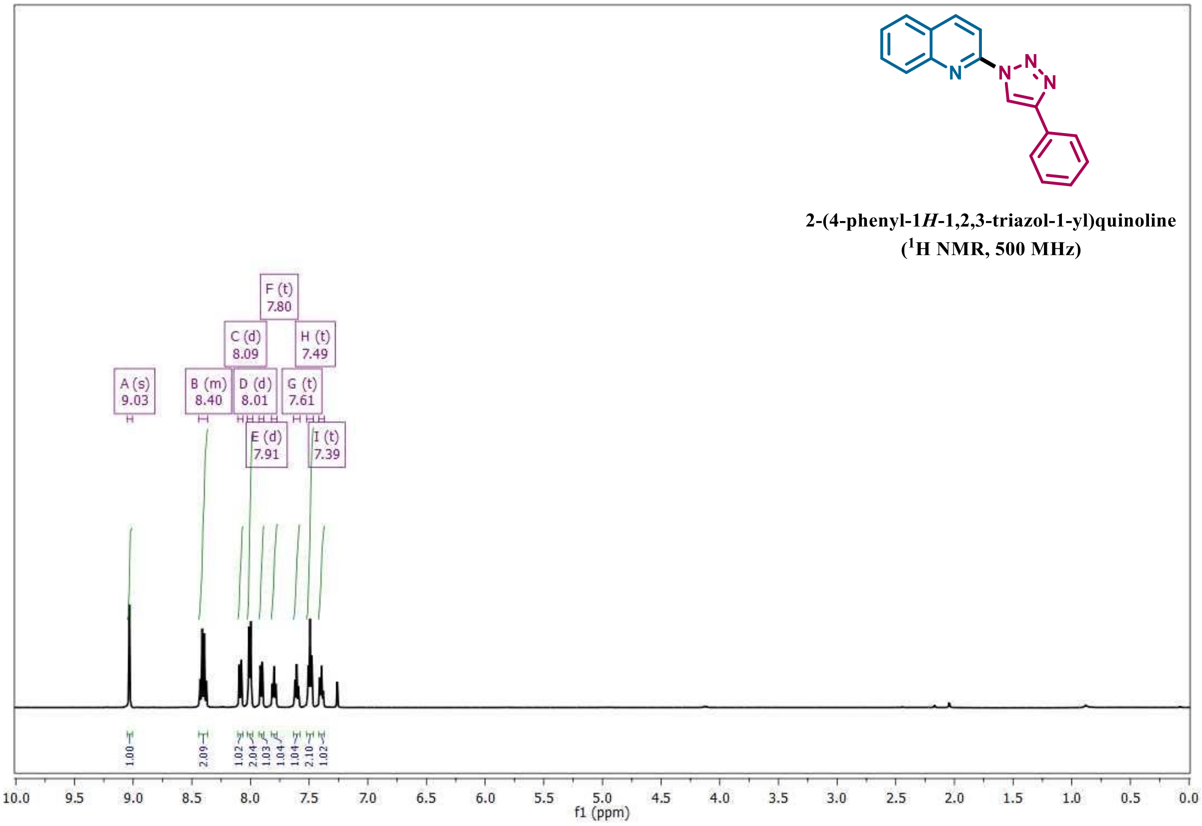

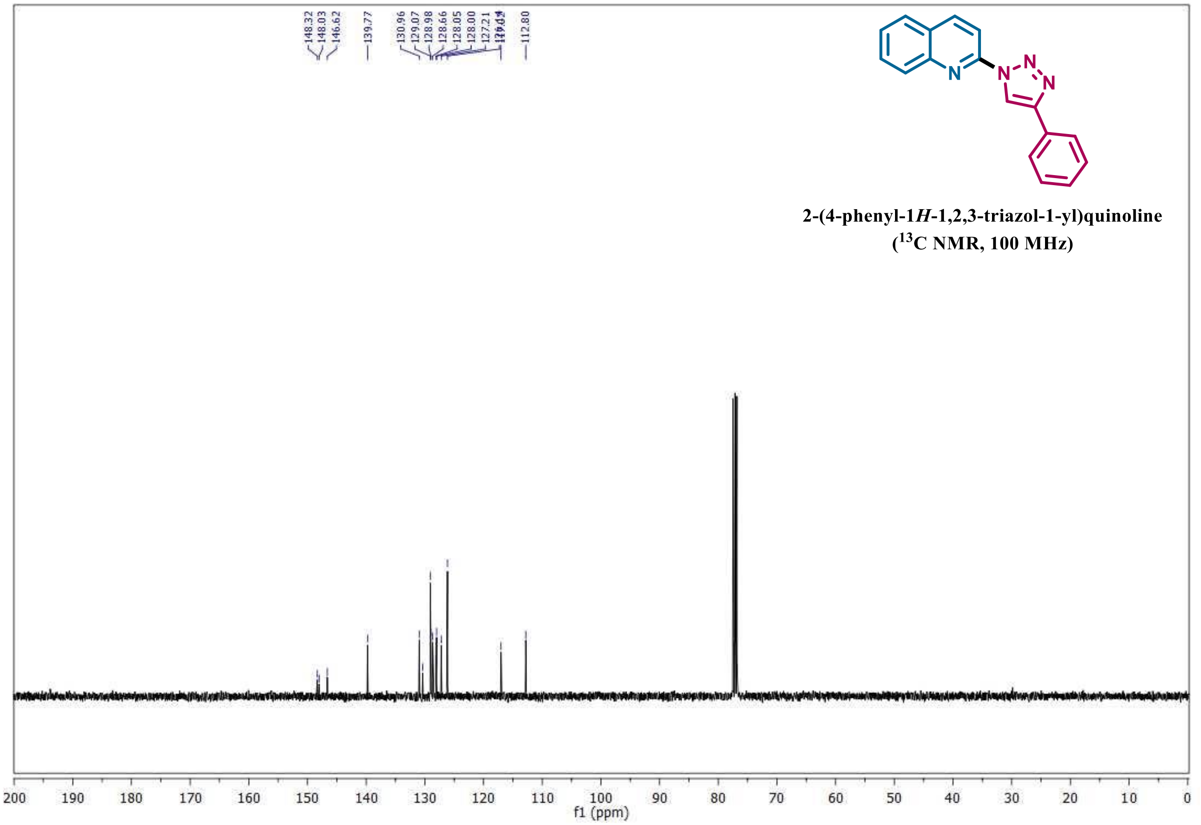

^{1}H and ^{13}C NMR Spectra of 3b

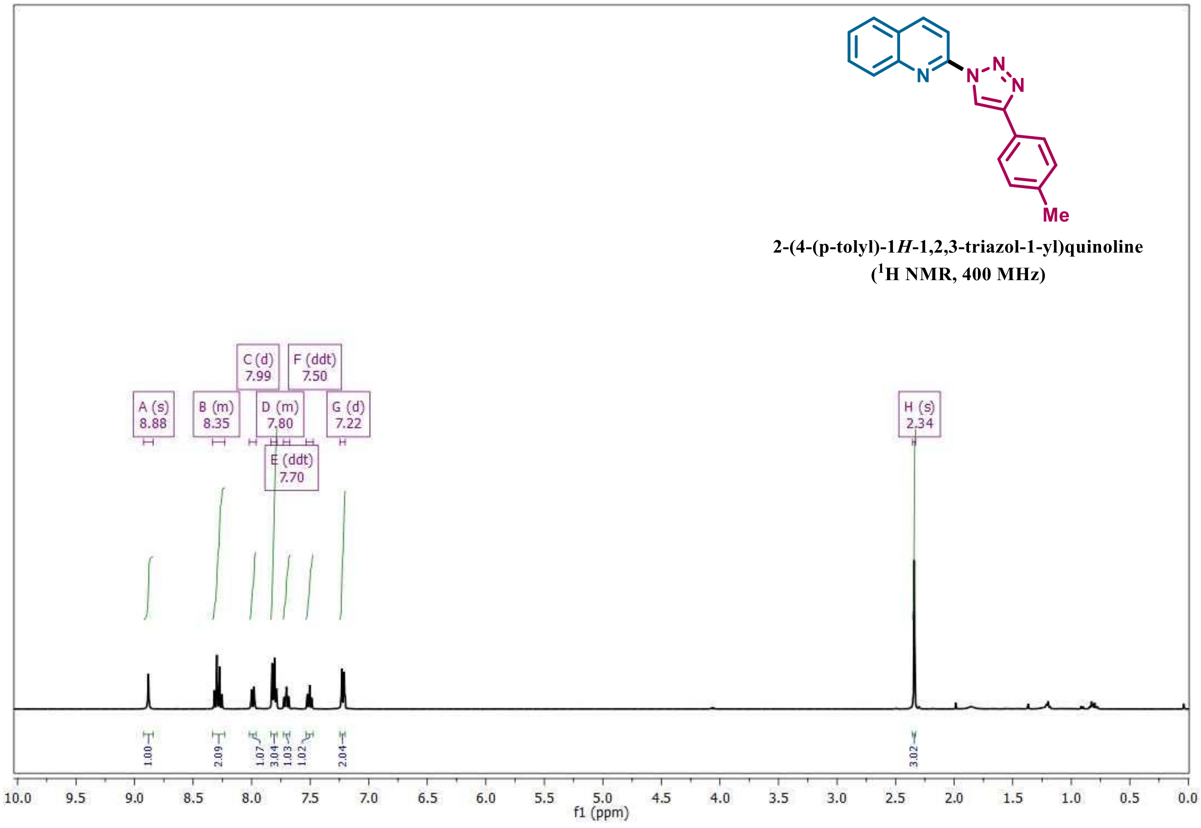

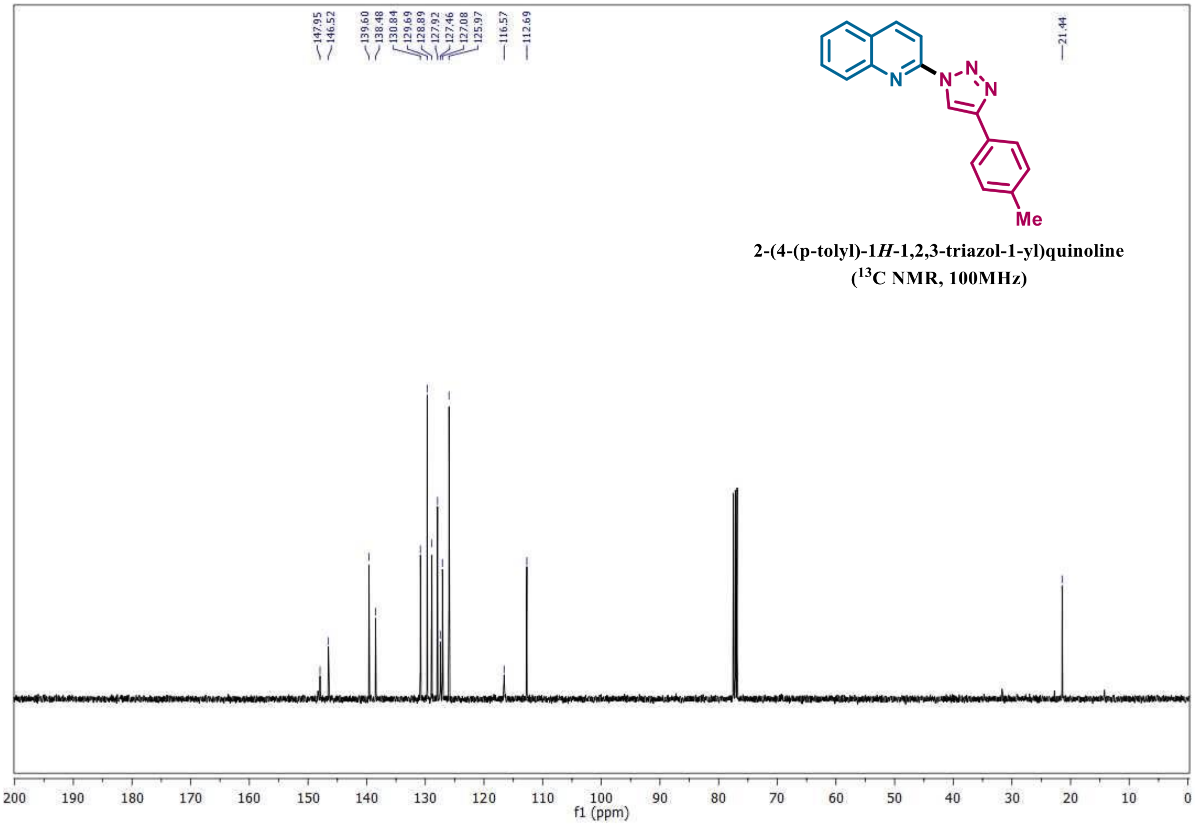

^{1}H and ^{13}C NMR Spectra of 3c

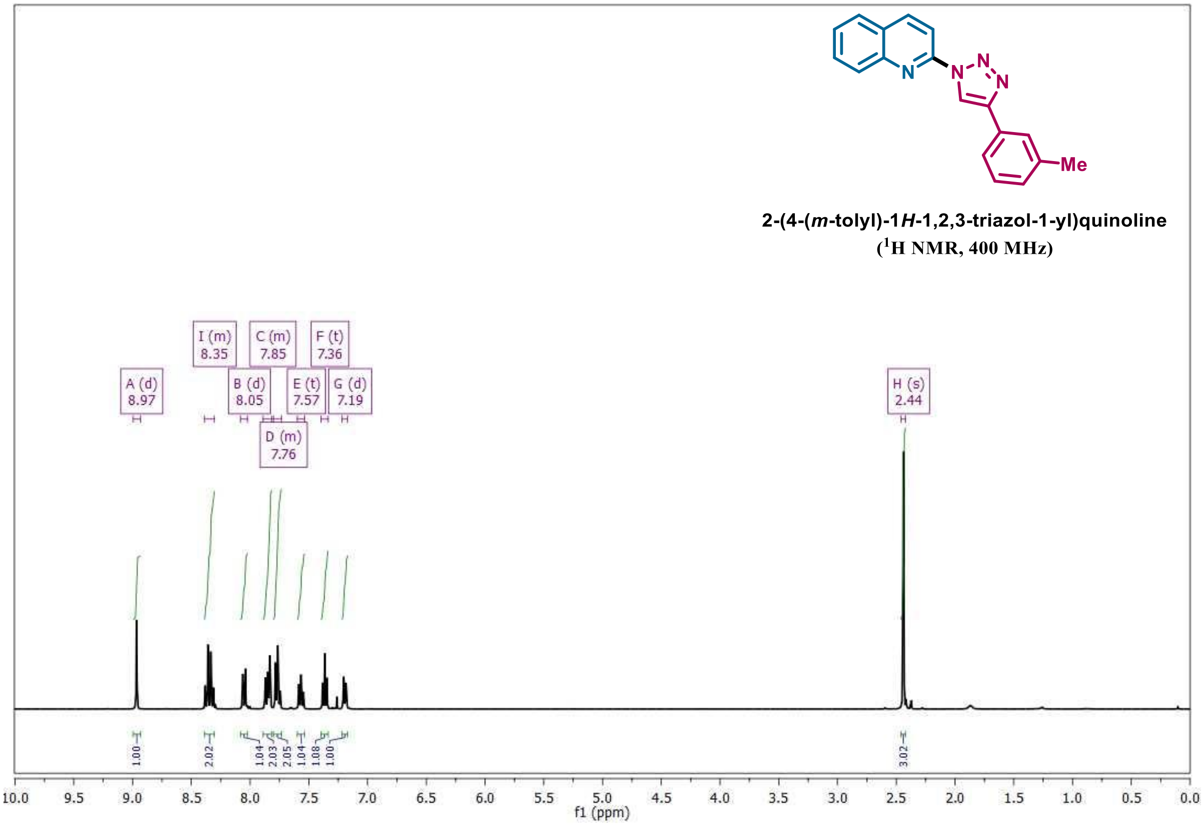

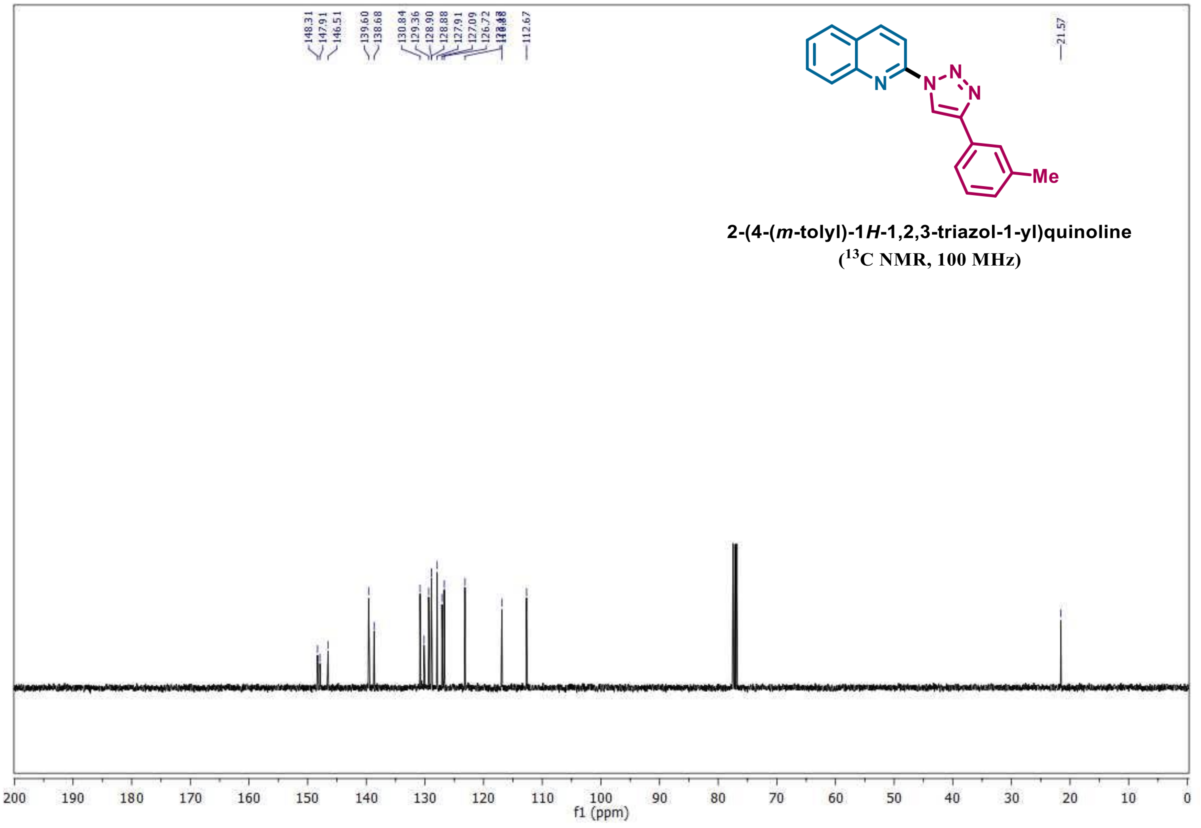

^{1}H and ^{13}C NMR Spectra of 3d

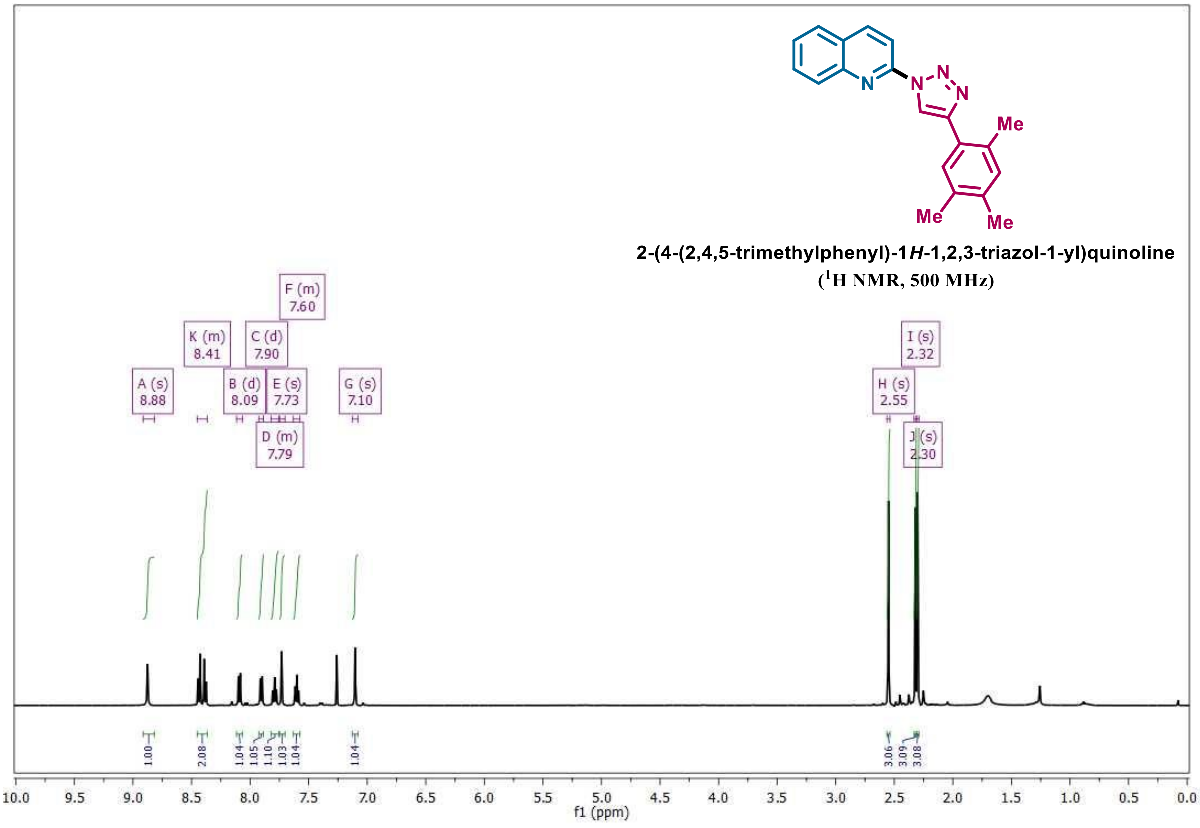

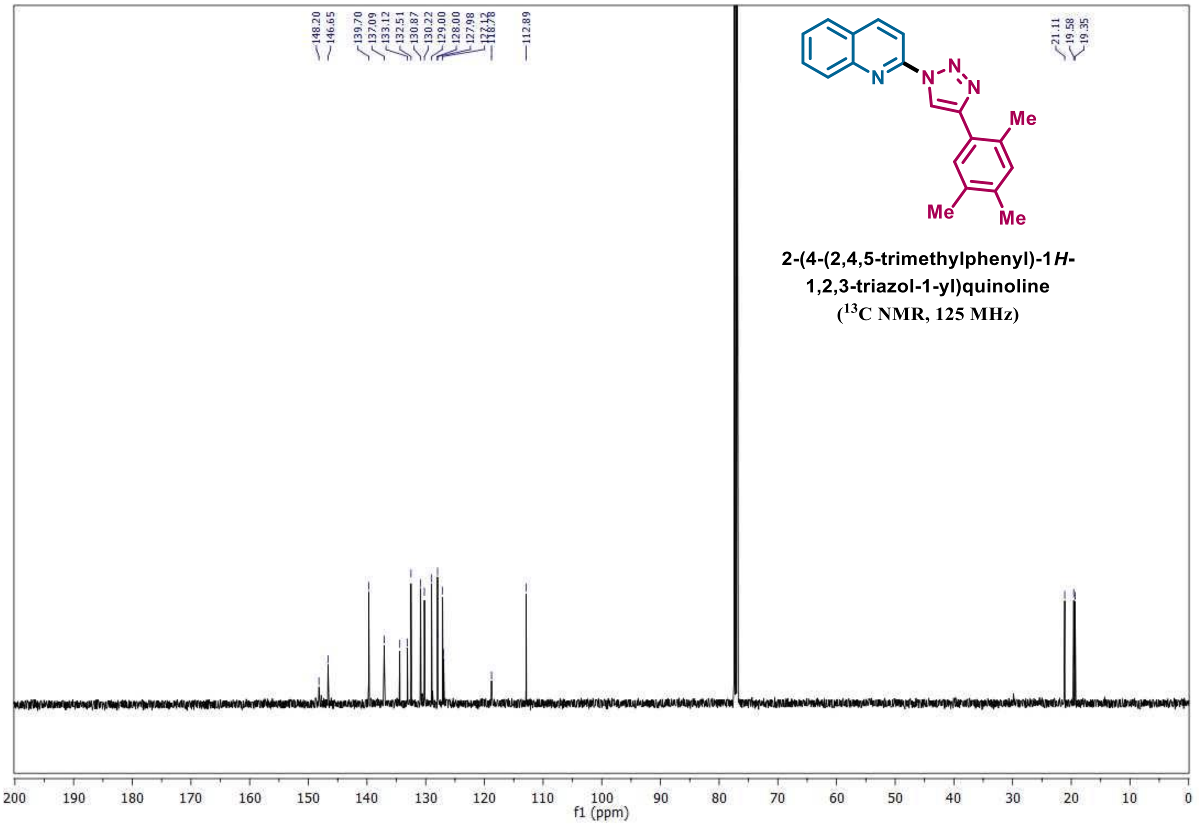

^{1}H and ^{13}C NMR Spectra of 3e

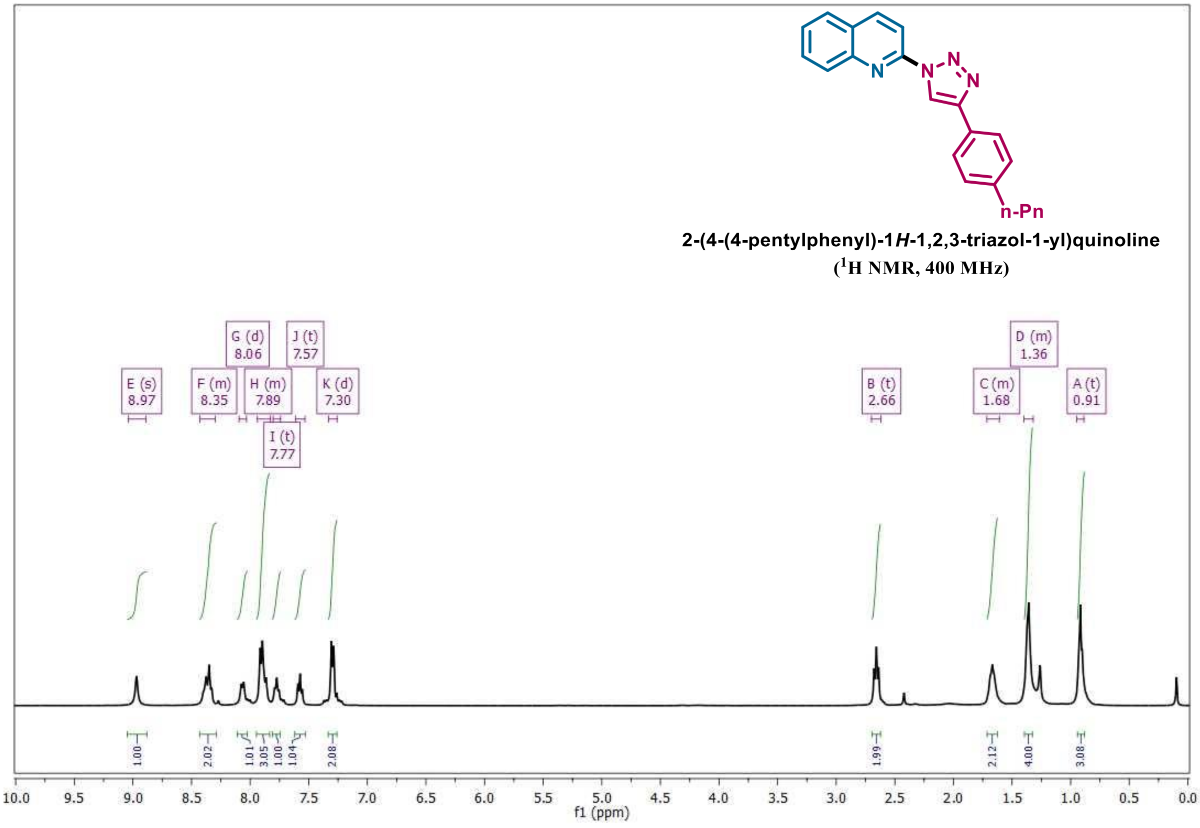

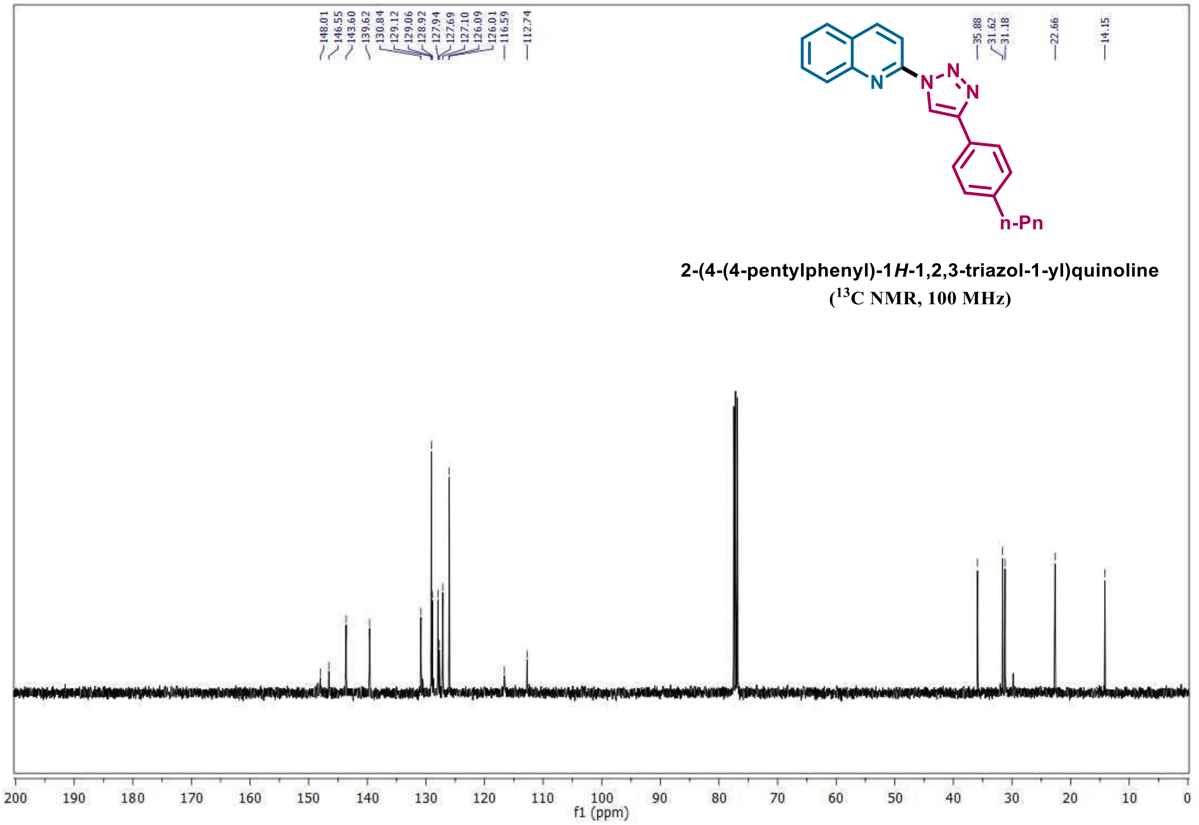

^{1}H and ^{13}C NMR Spectra of 3f

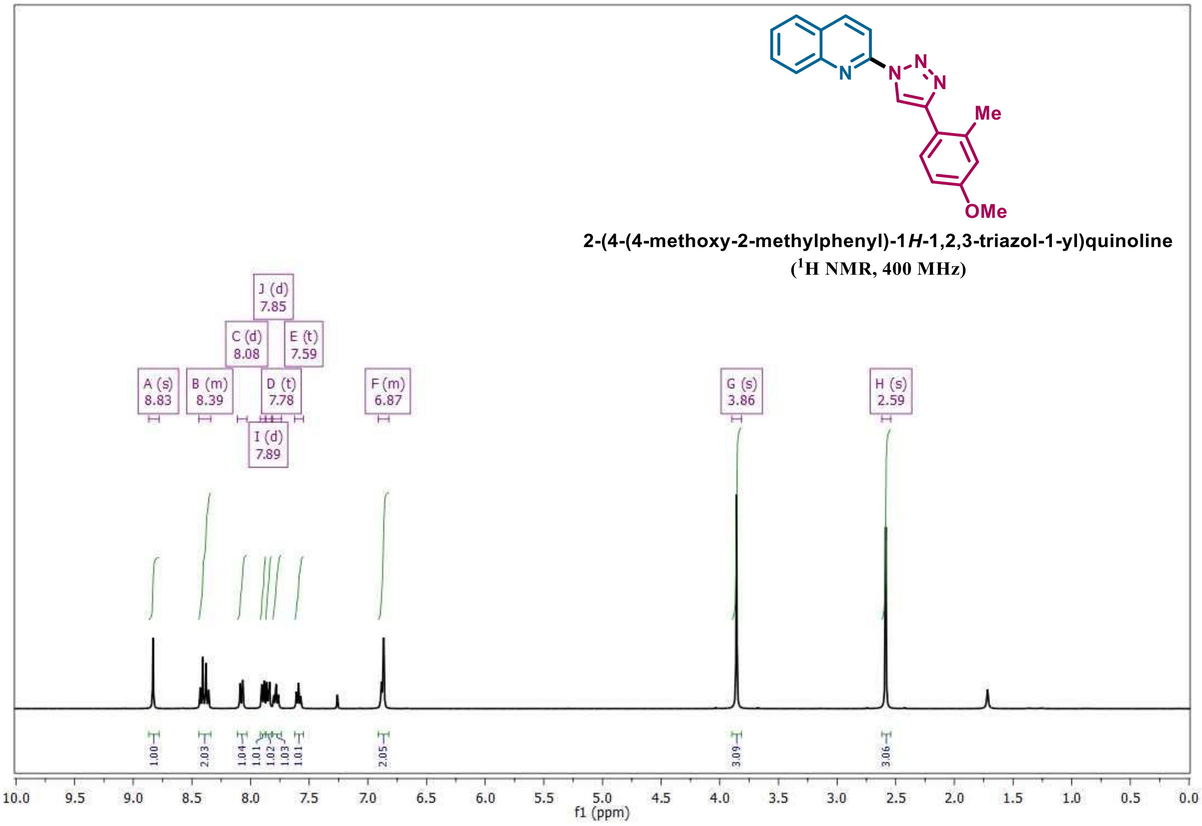

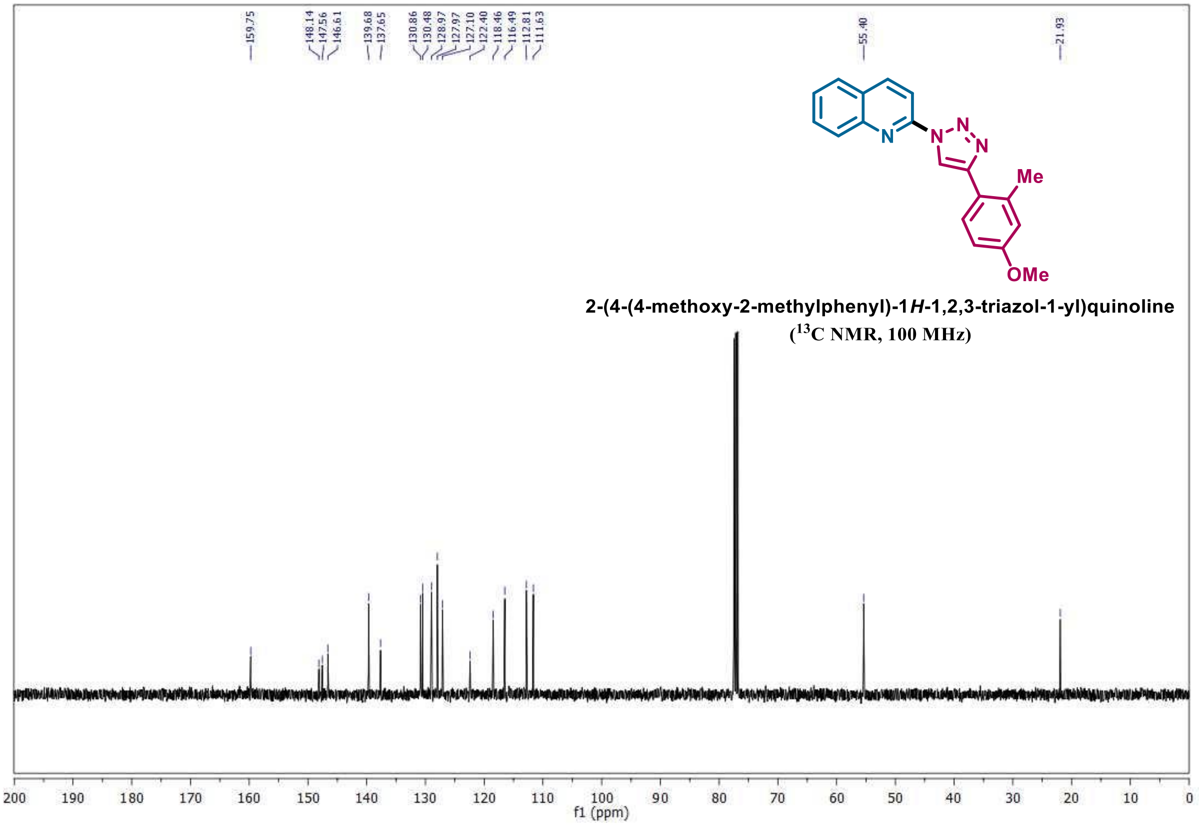

^{1}H and ^{13}C NMR Spectra of 3g

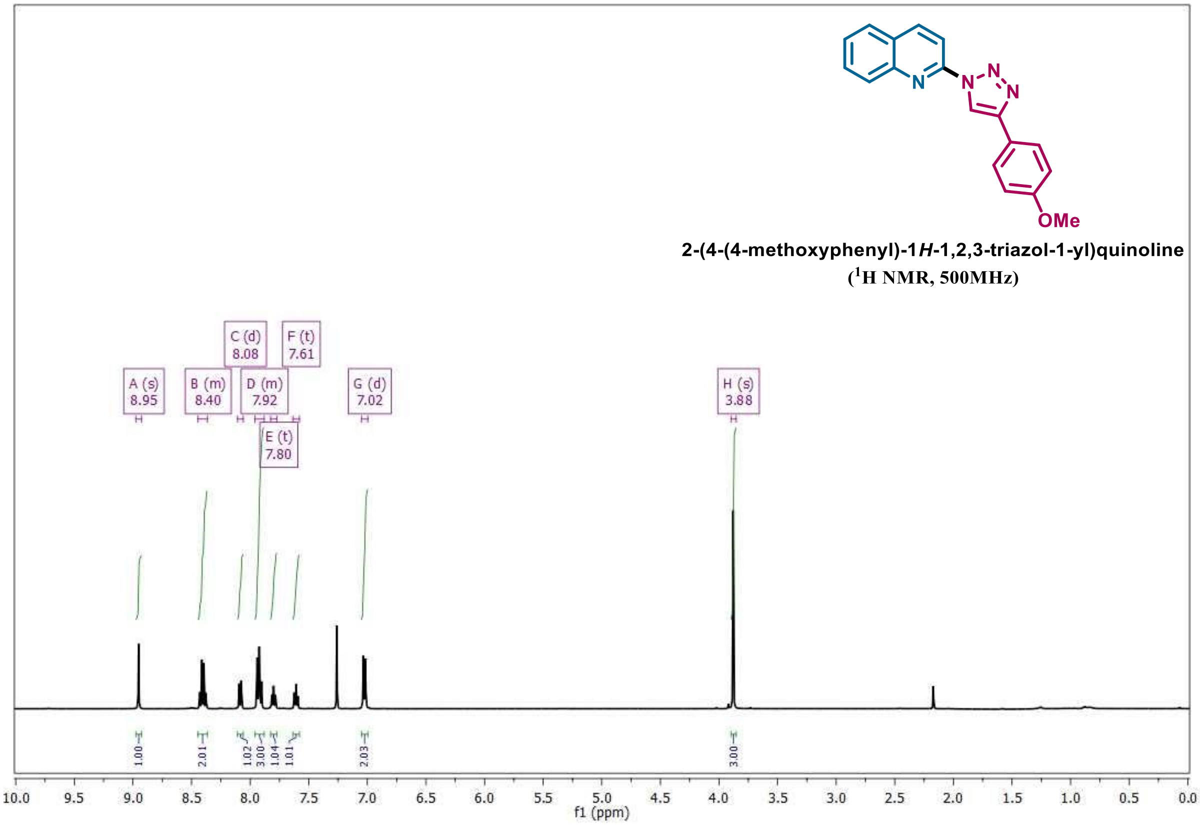

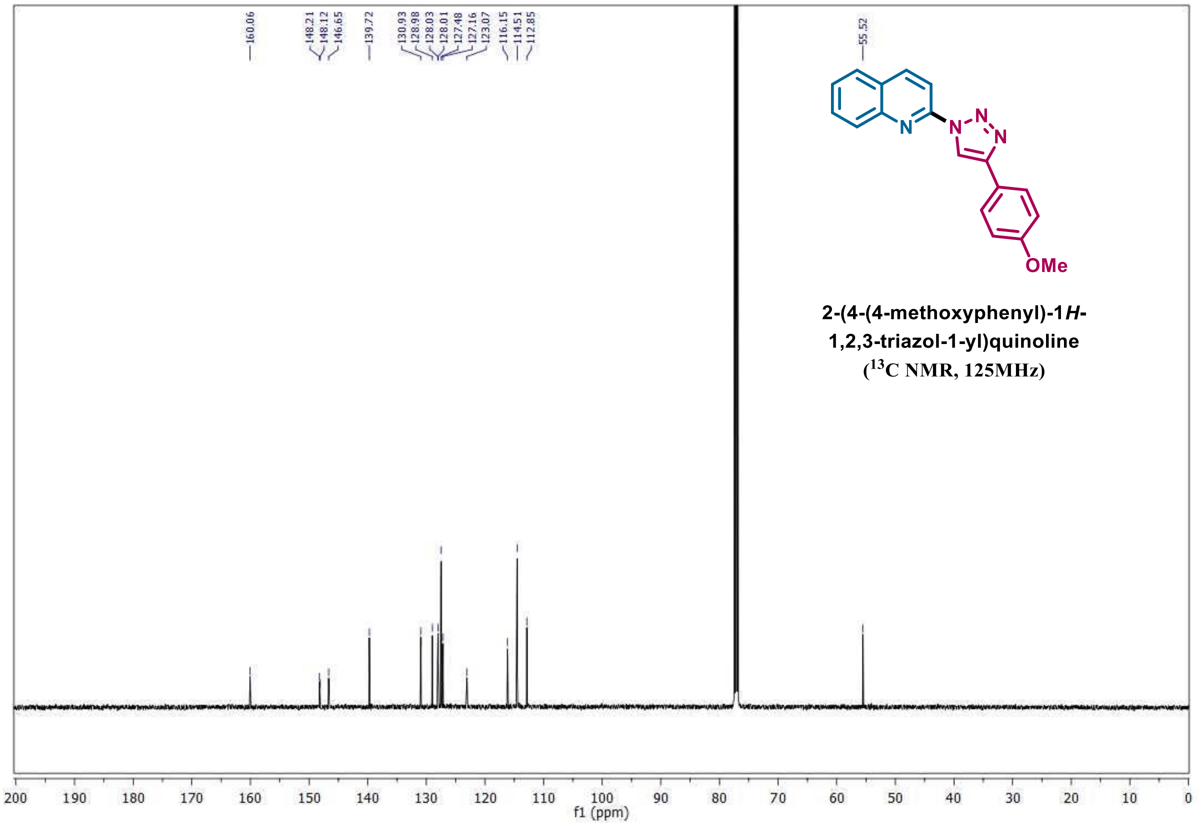

^{1}H and ^{13}C NMR Spectra of 3h

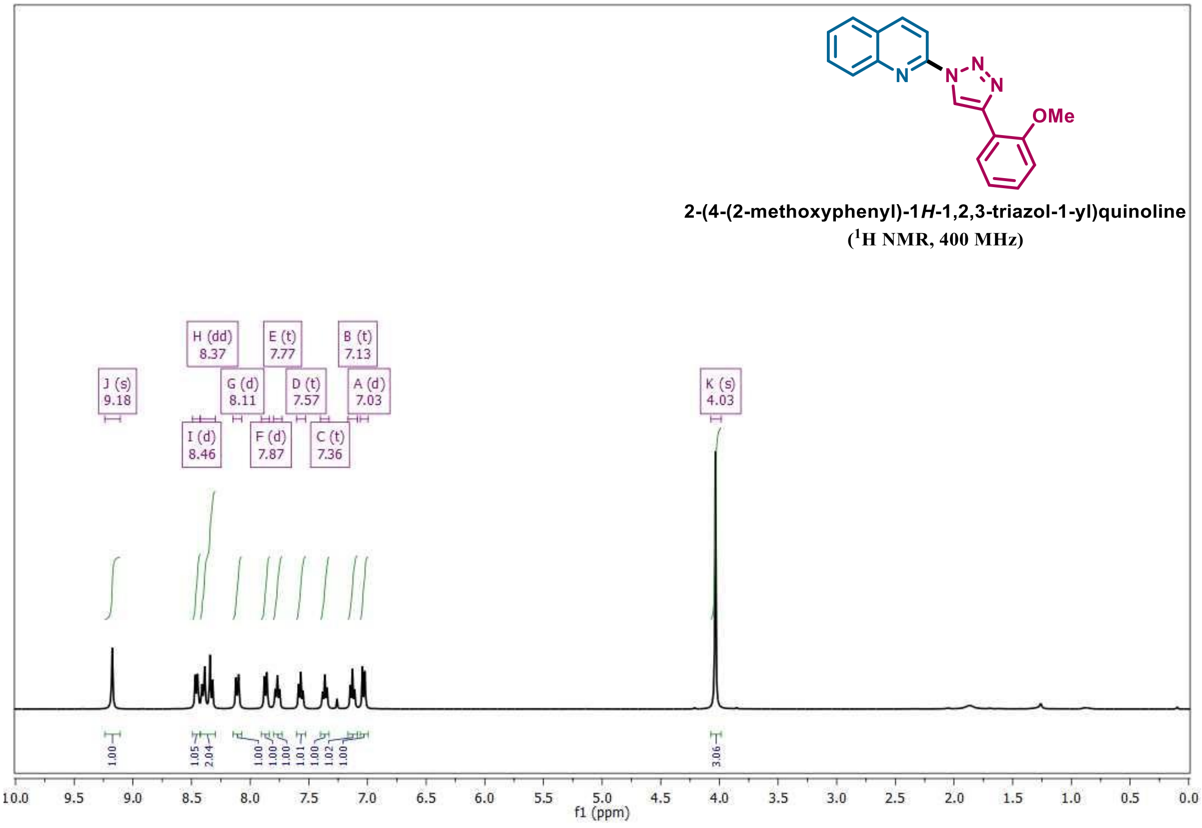

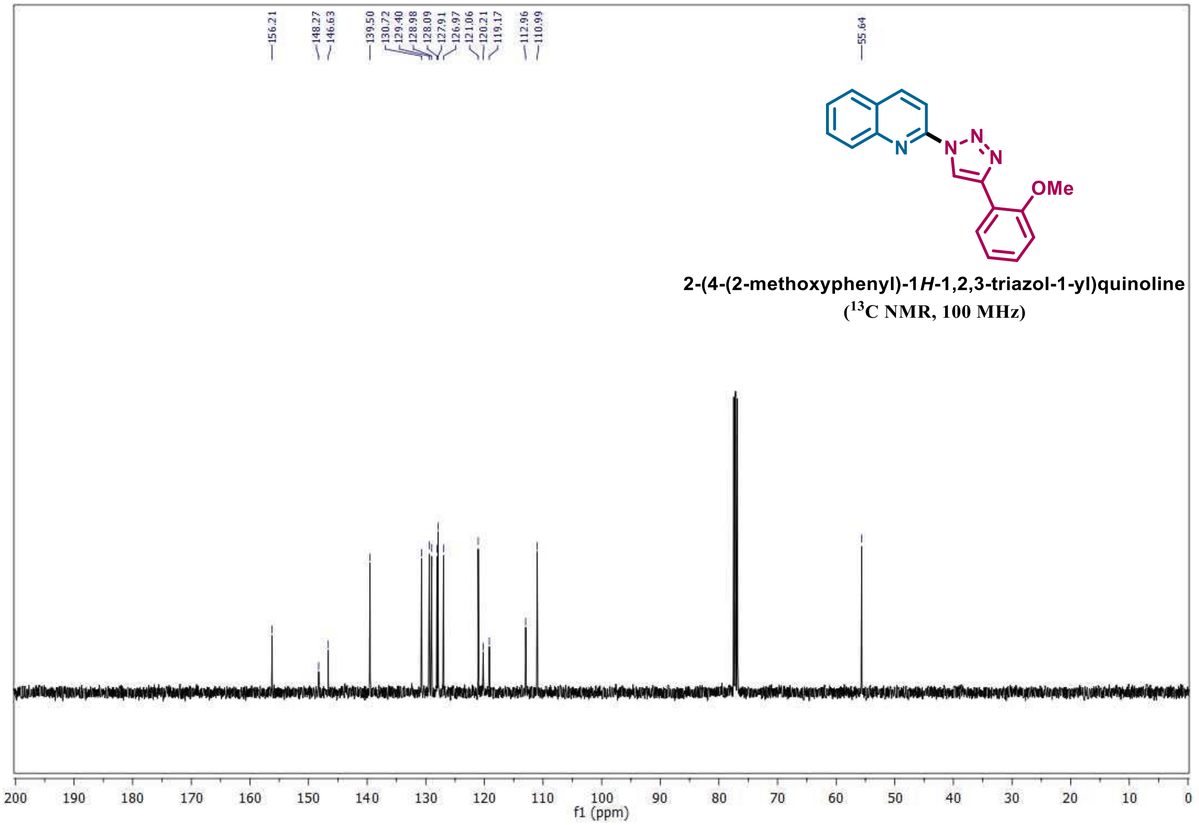

^{1}H and ^{13}C NMR Spectra of 3i

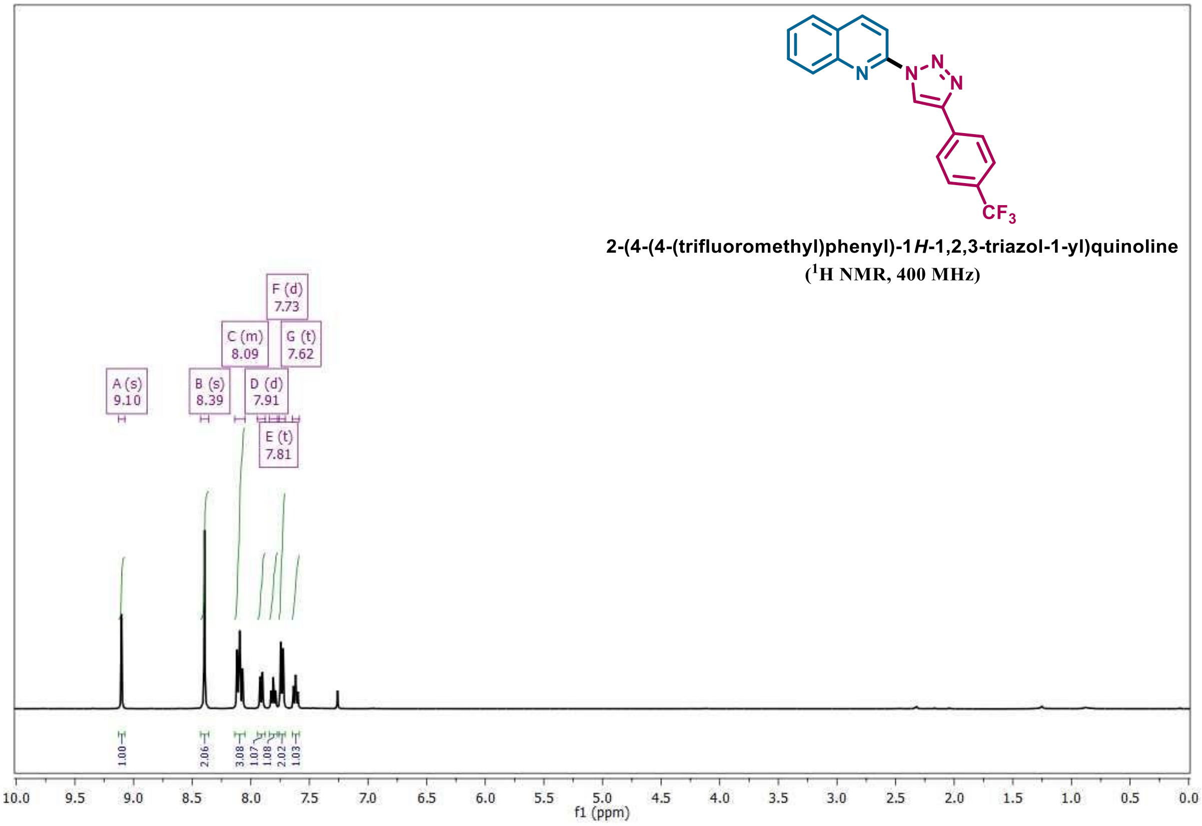

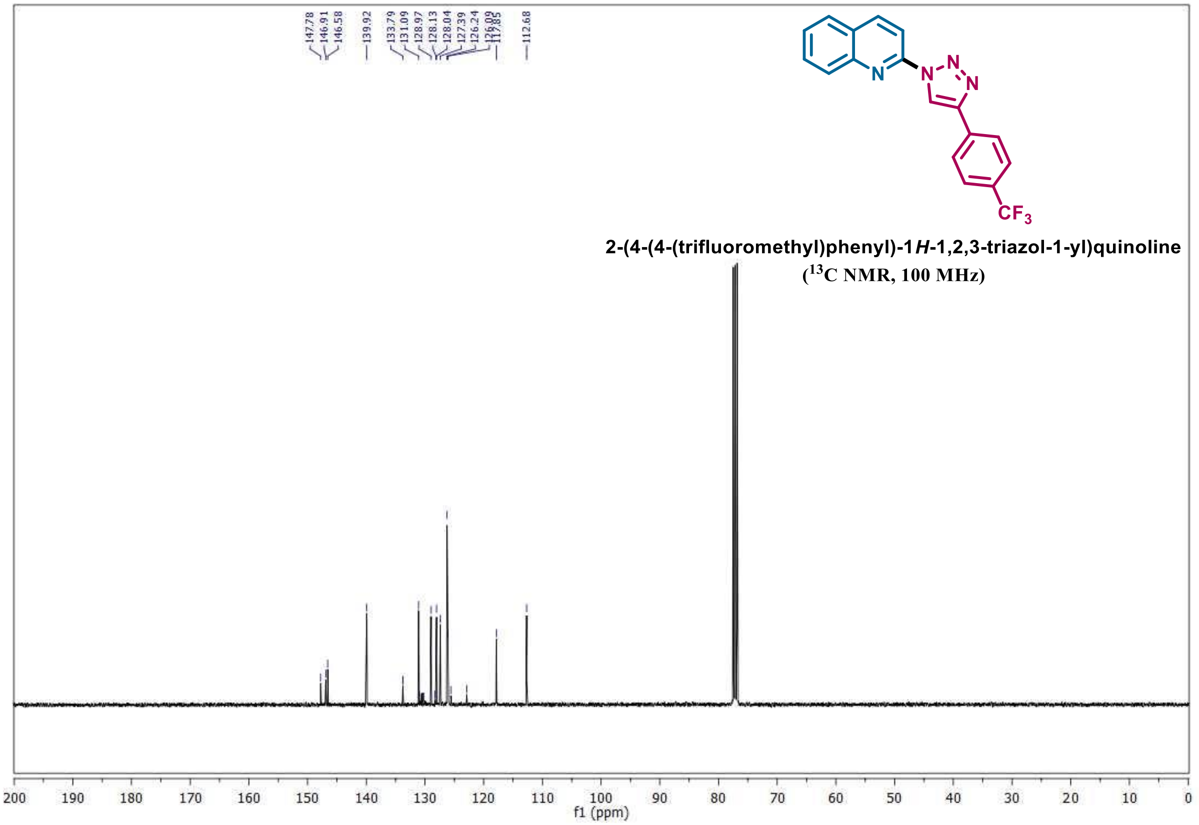

^{1}H and ^{13}C NMR Spectra of 3j

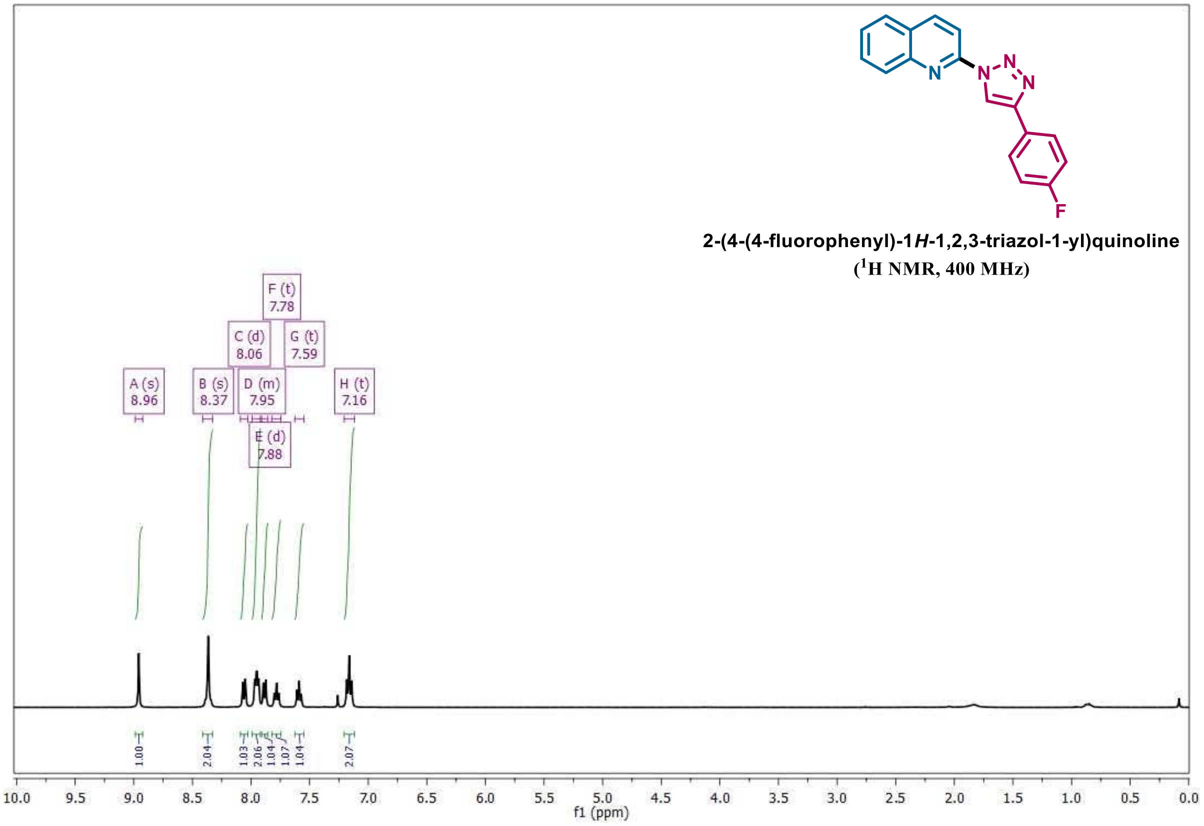

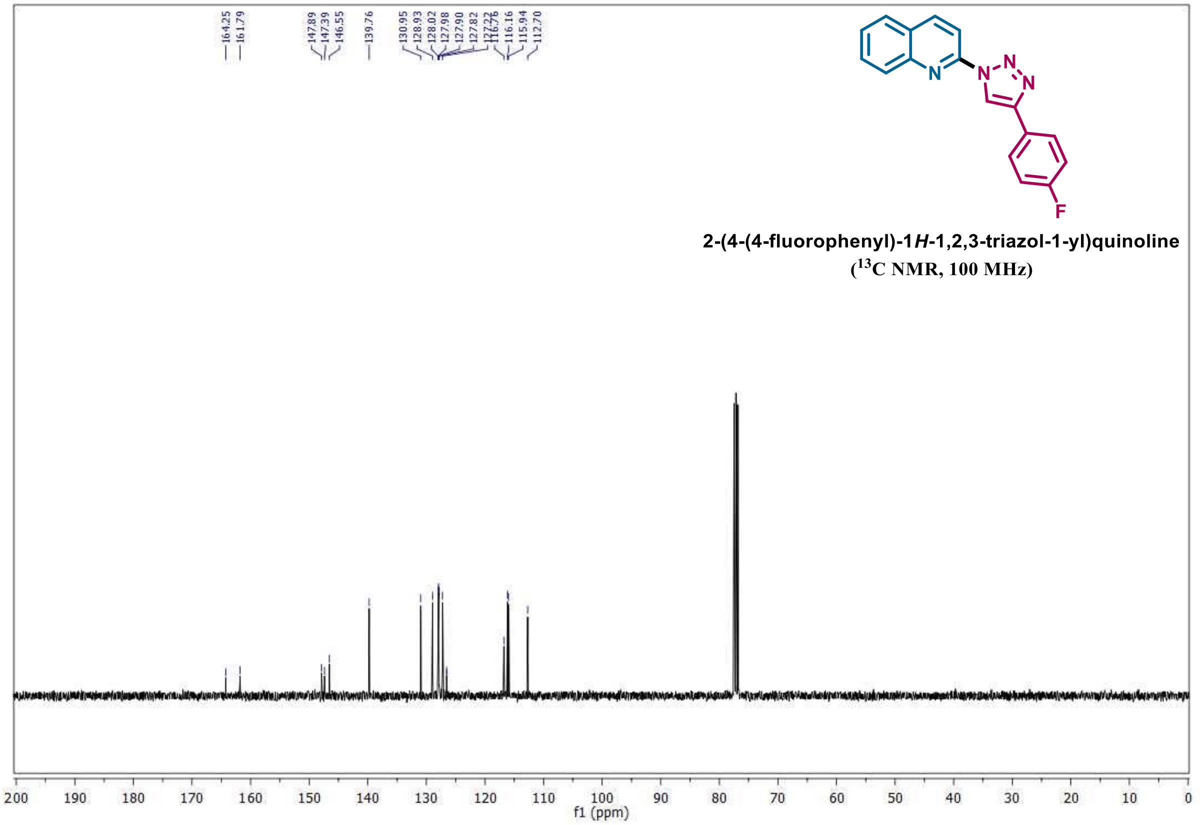

^{1}H and ^{13}C NMR Spectra of 3k

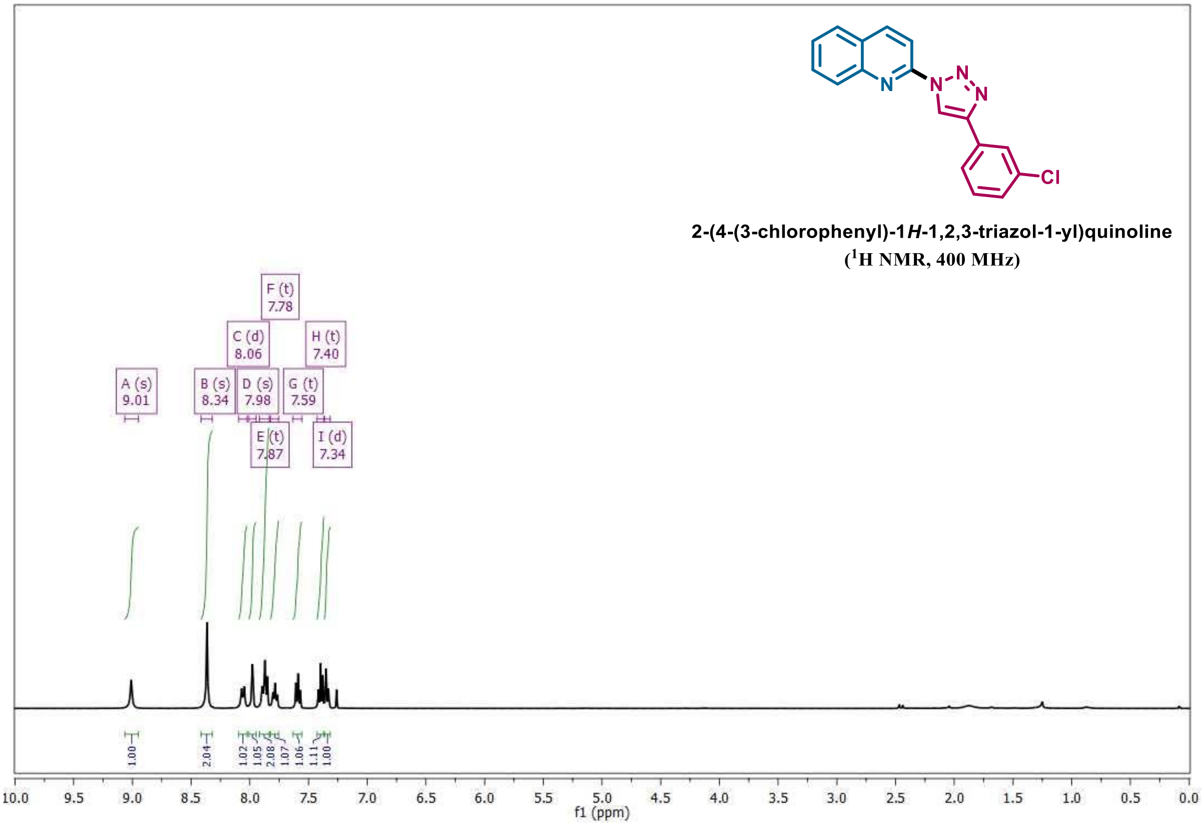

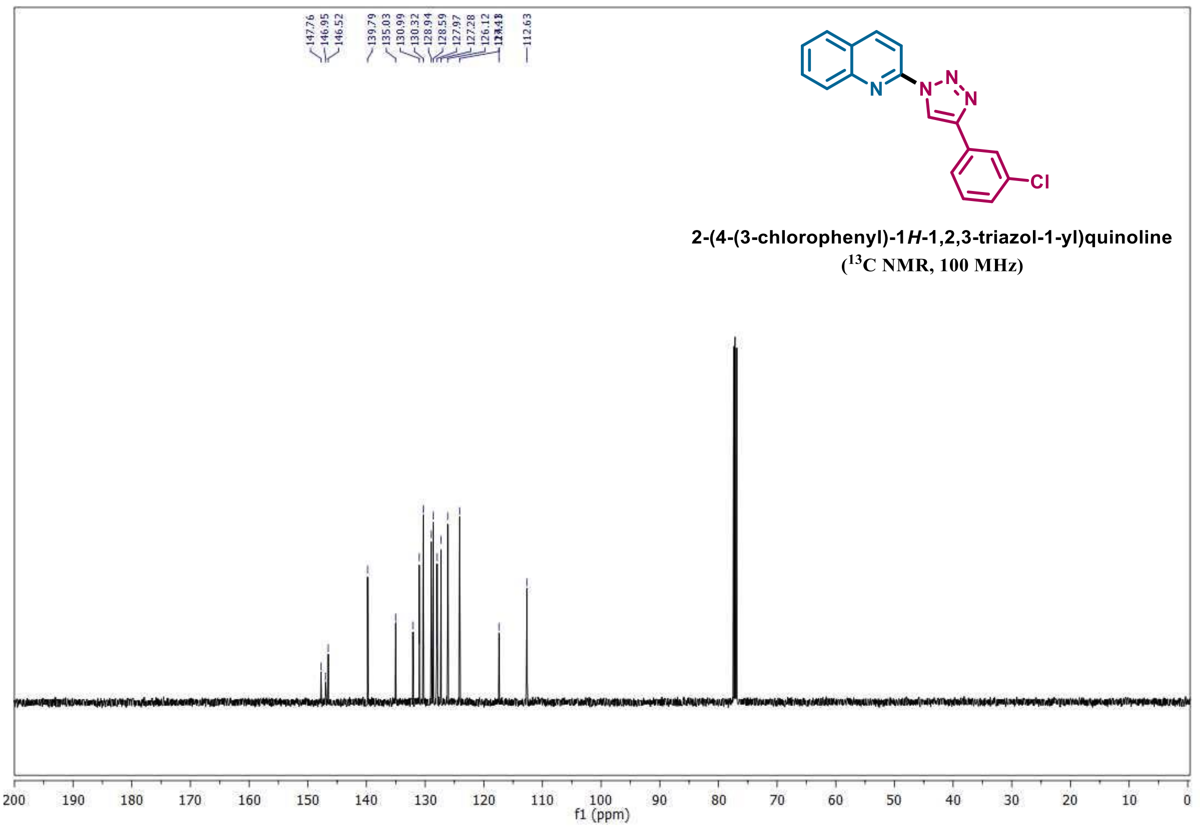

^{1}H and ^{13}C NMR Spectra of 3l

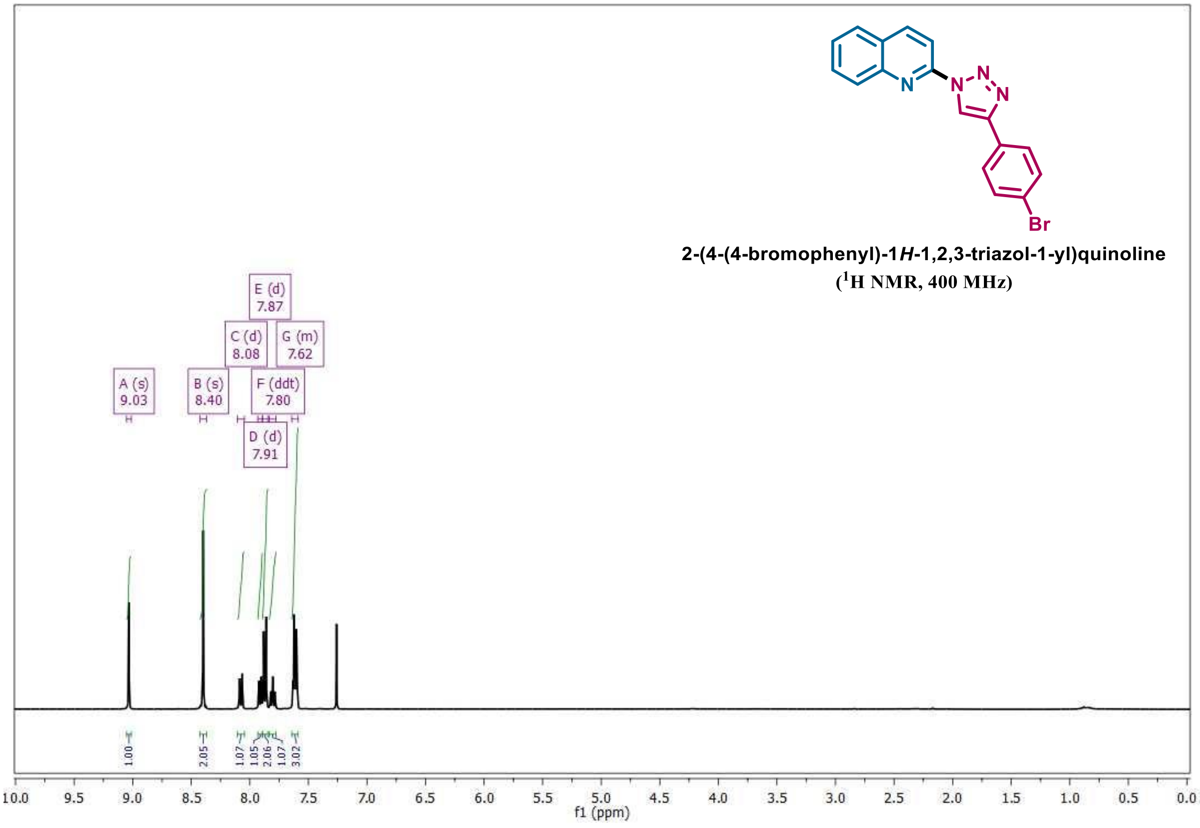

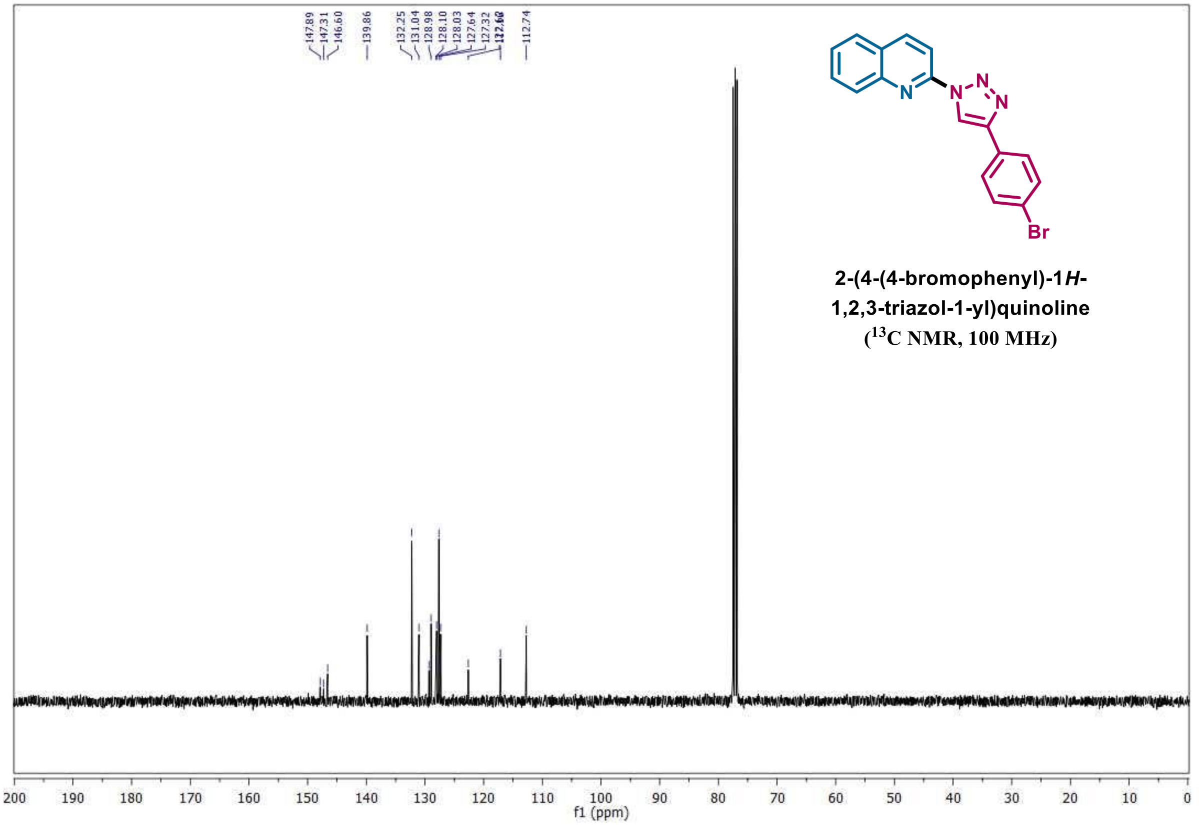

^{1}H and ^{13}C NMR Spectra of 3m

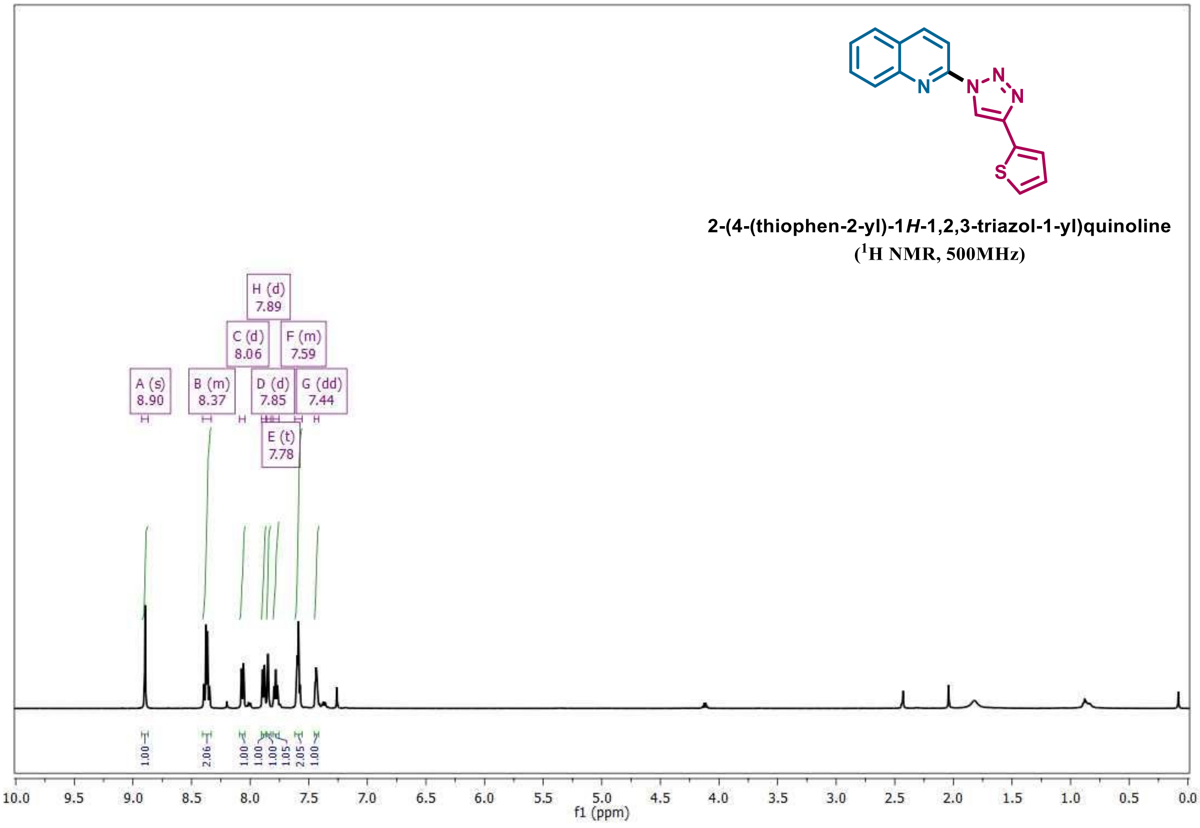

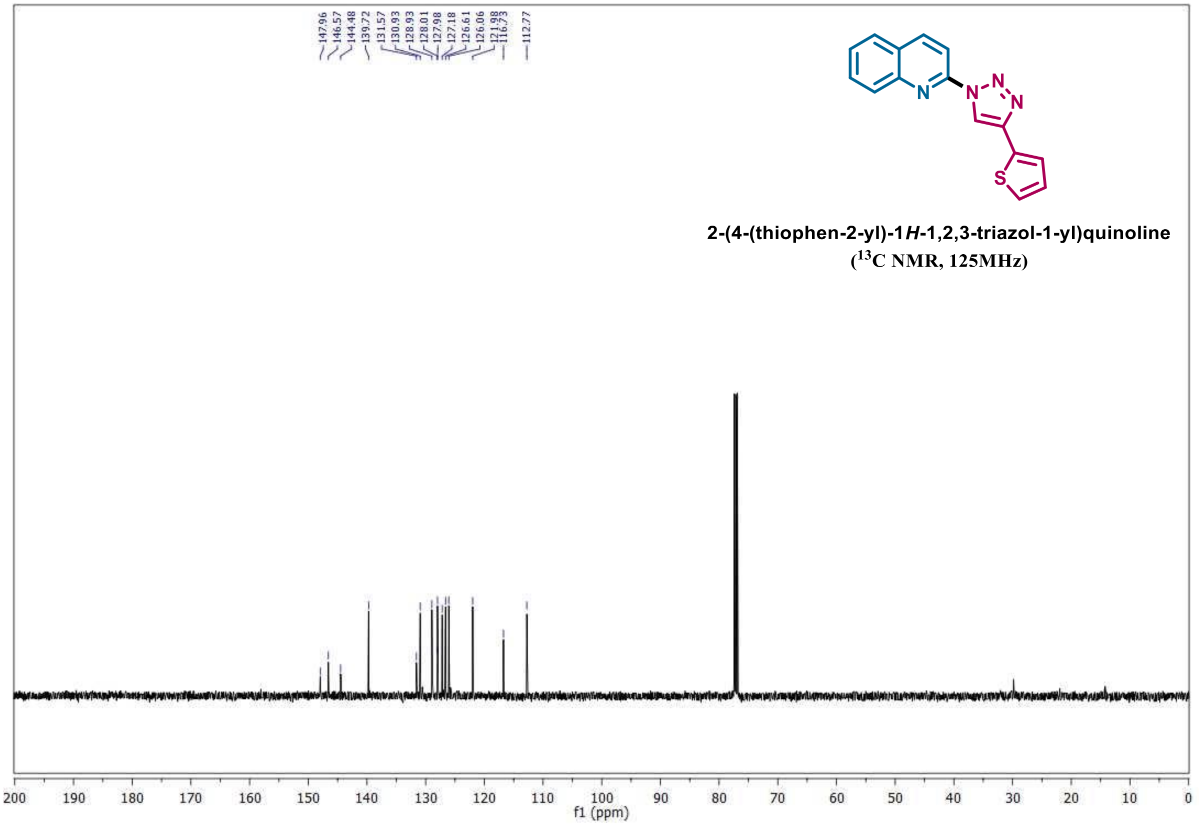

^{1}H and ^{13}C NMR Spectra of 3n

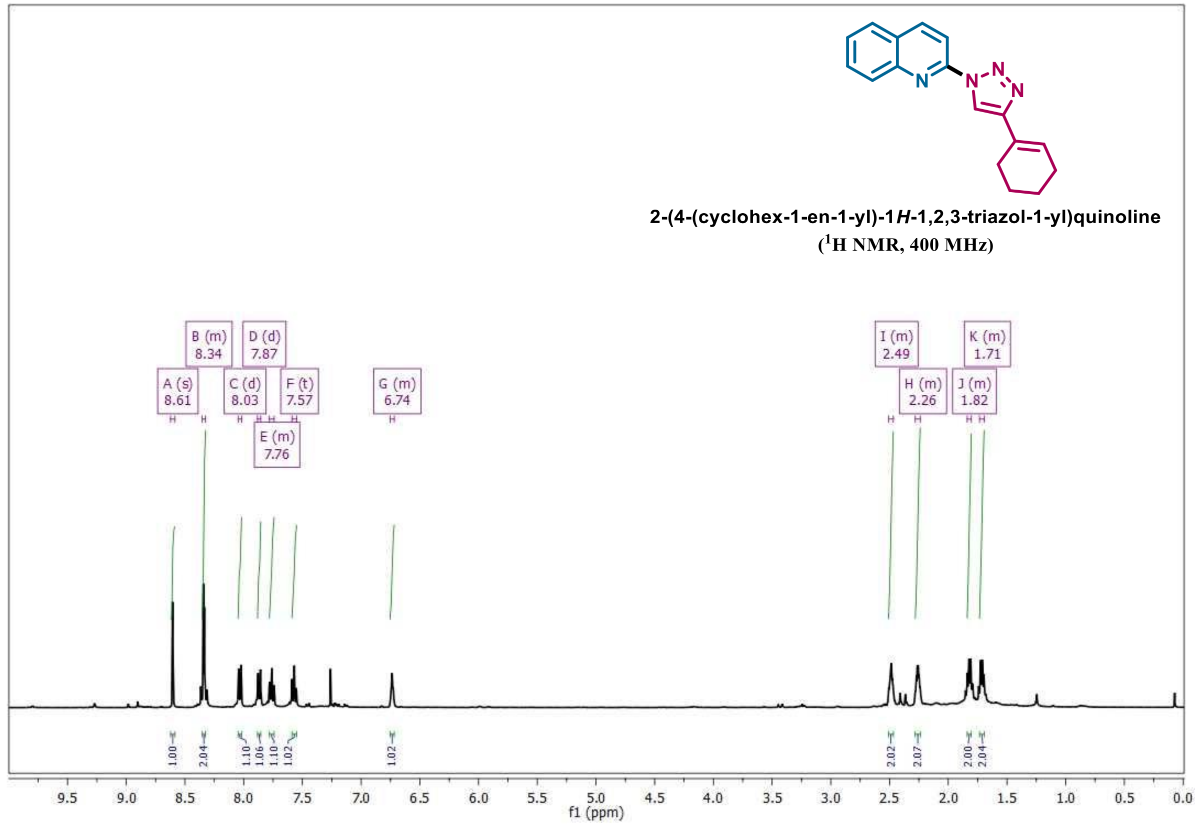

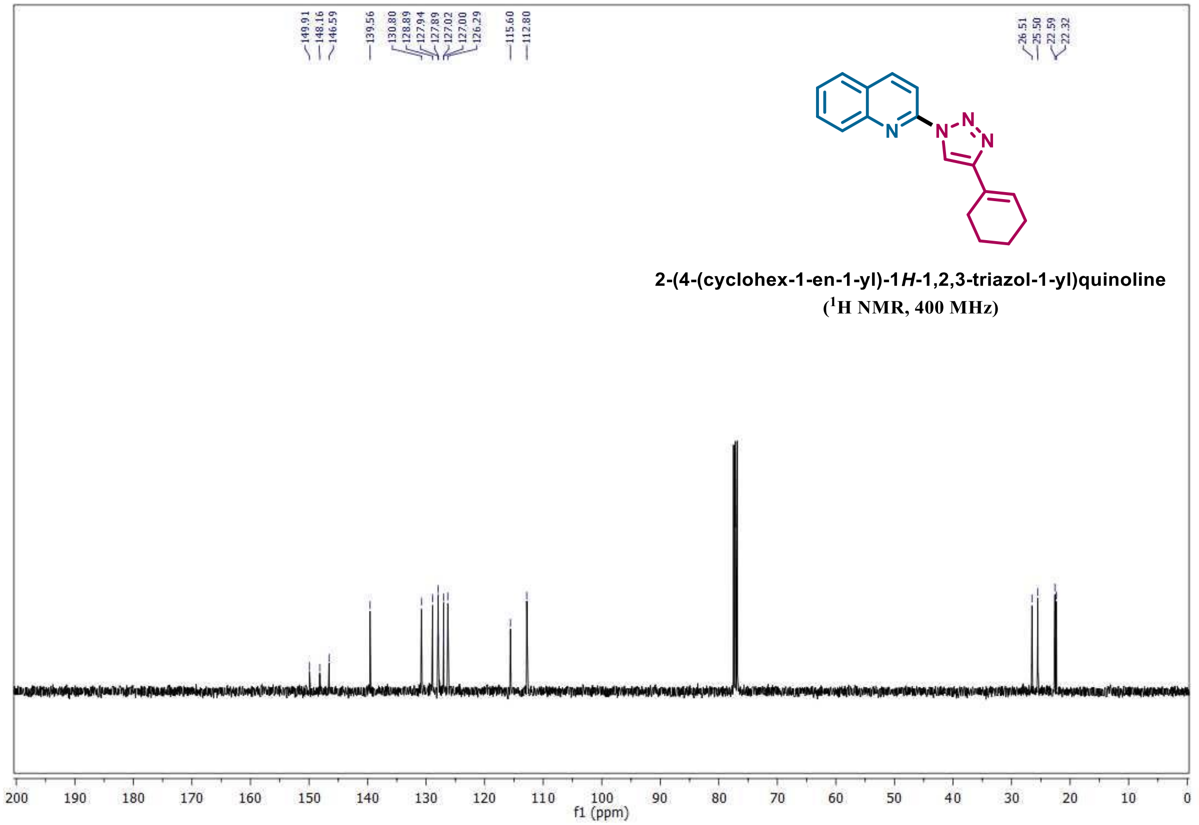

^{1}H and ^{13}C NMR Spectra of 3o

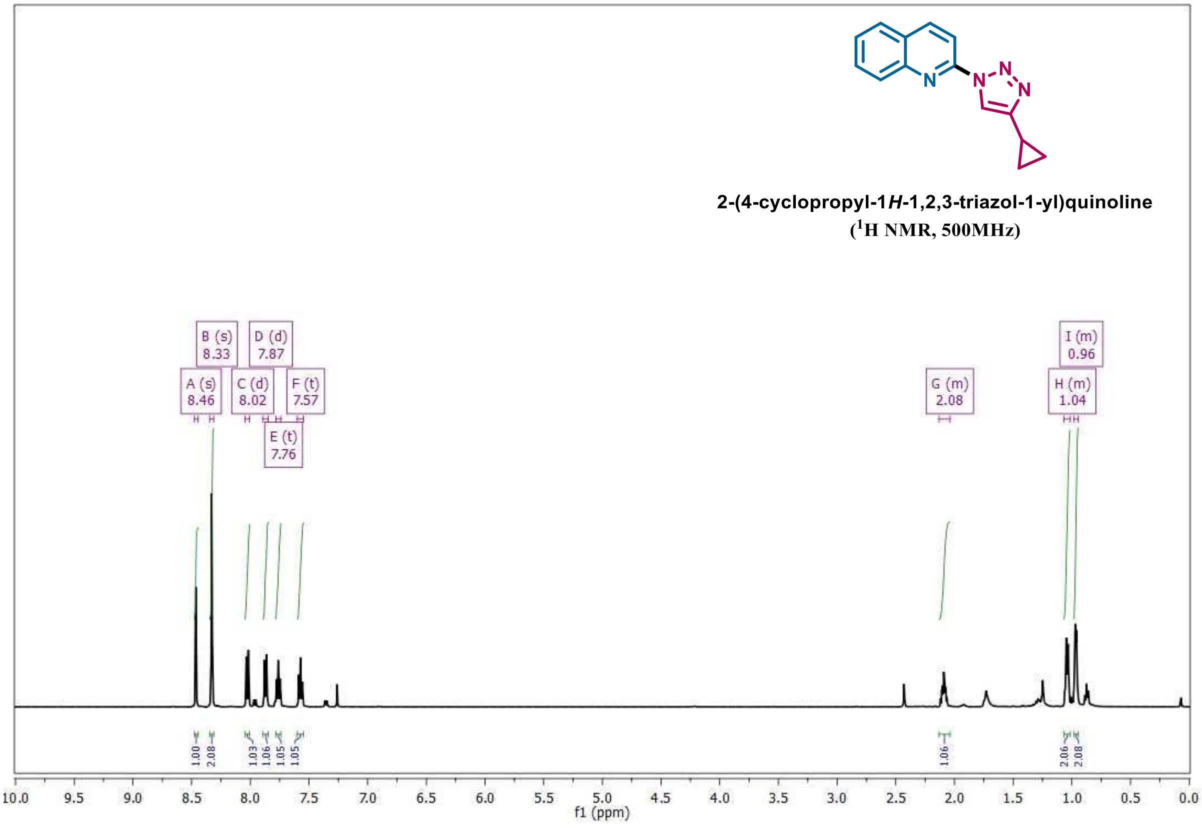

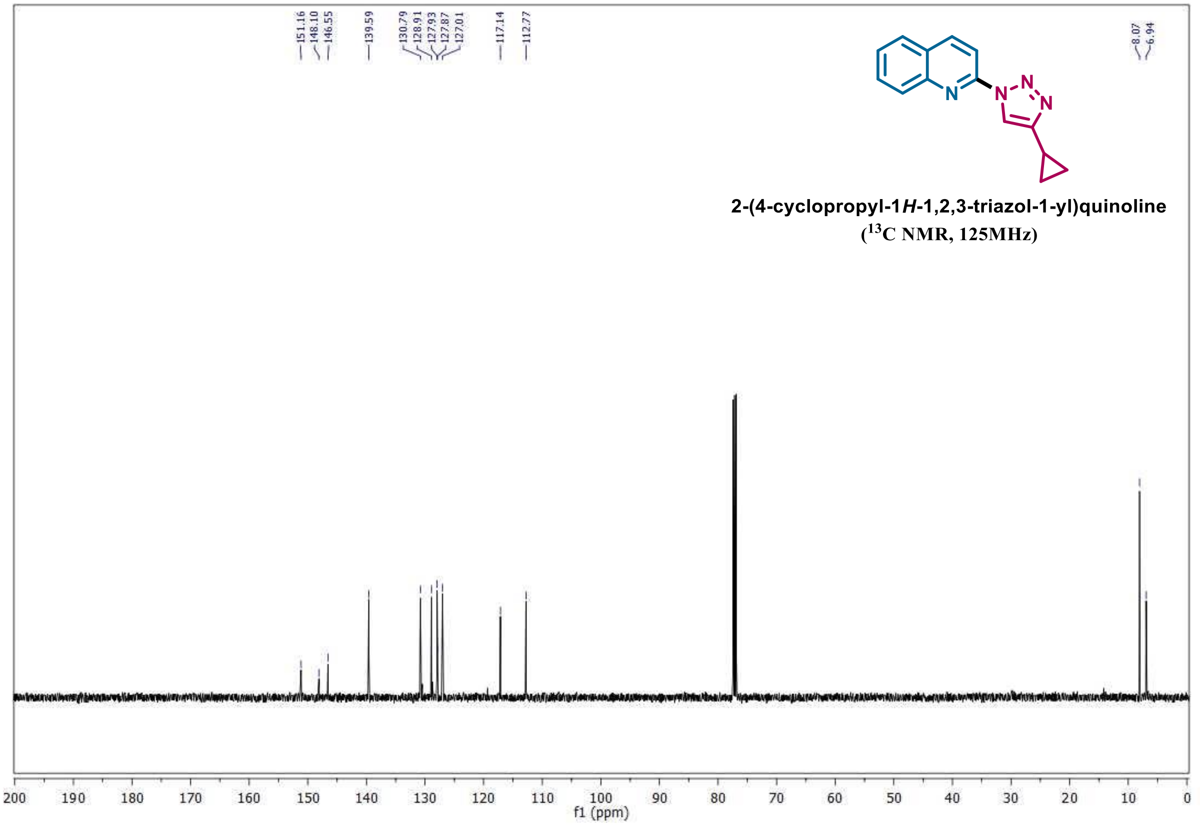

^{1}H and ^{13}C NMR Spectra of 3p

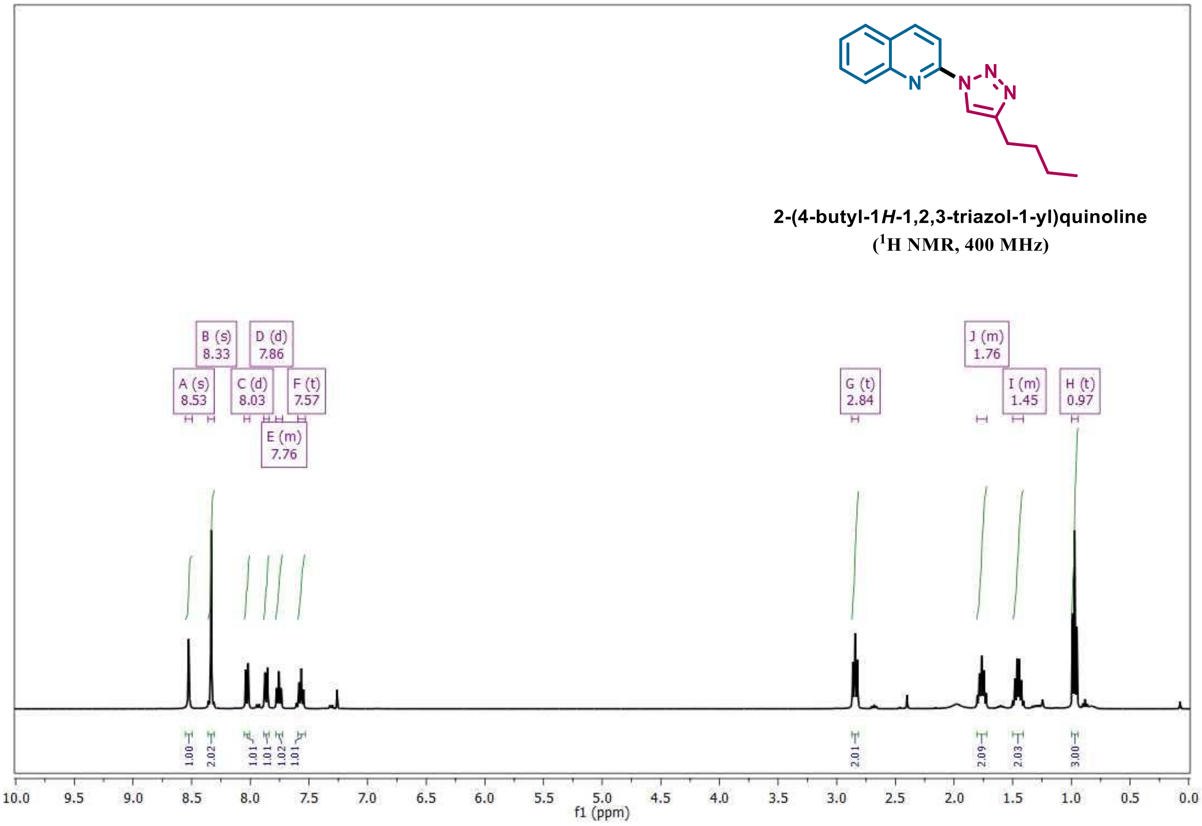

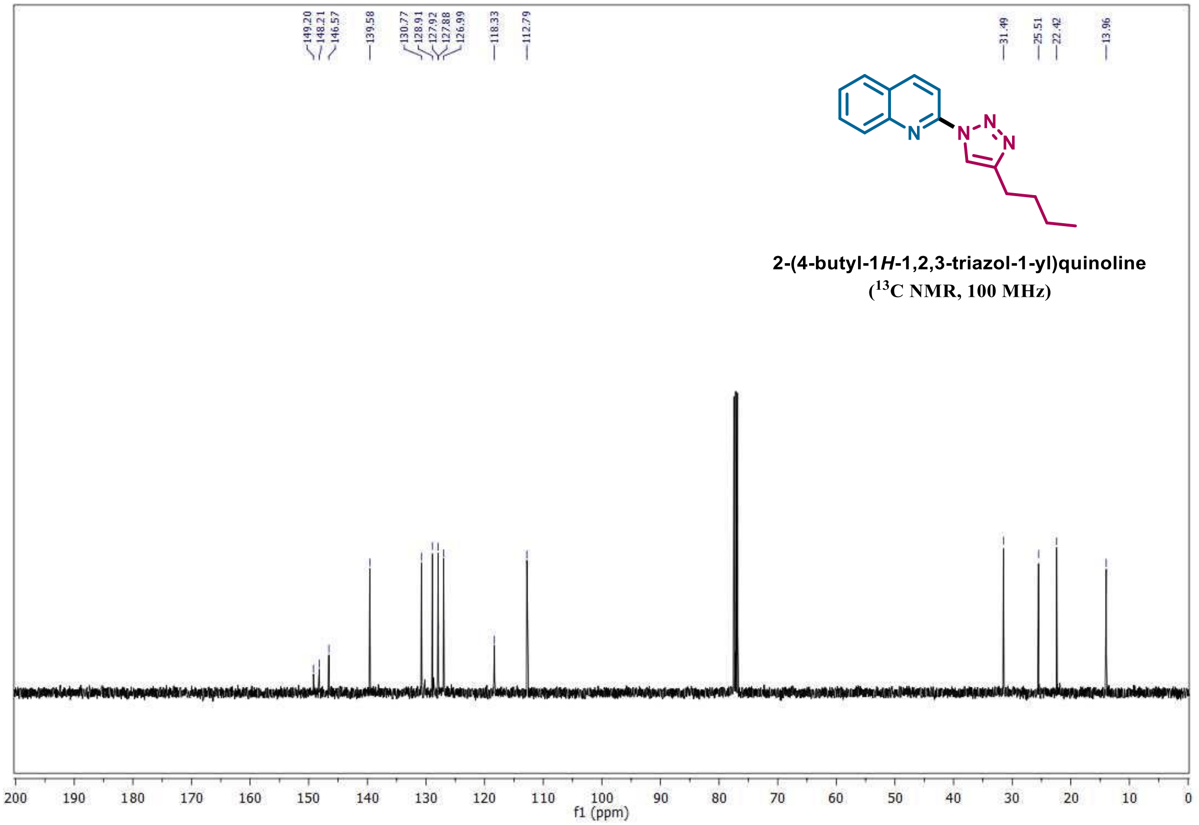

^{1}H and ^{13}C NMR Spectra of 3q

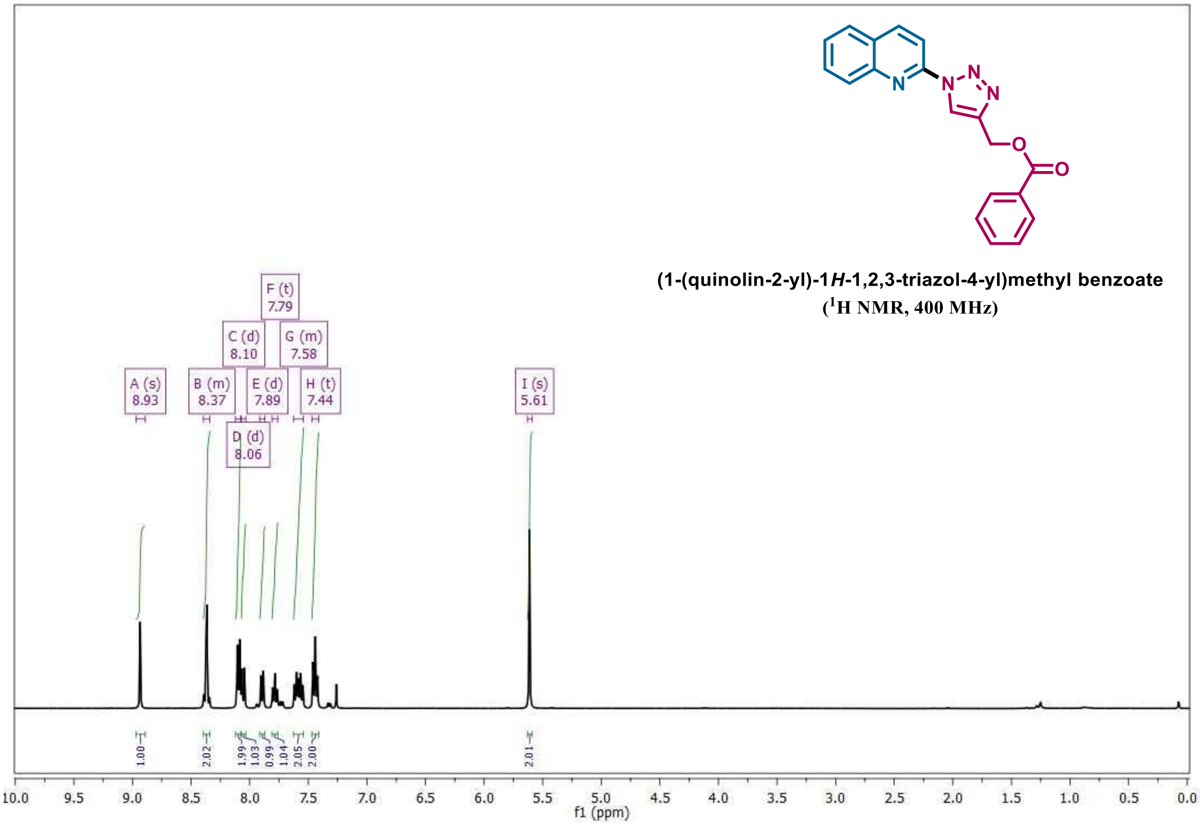

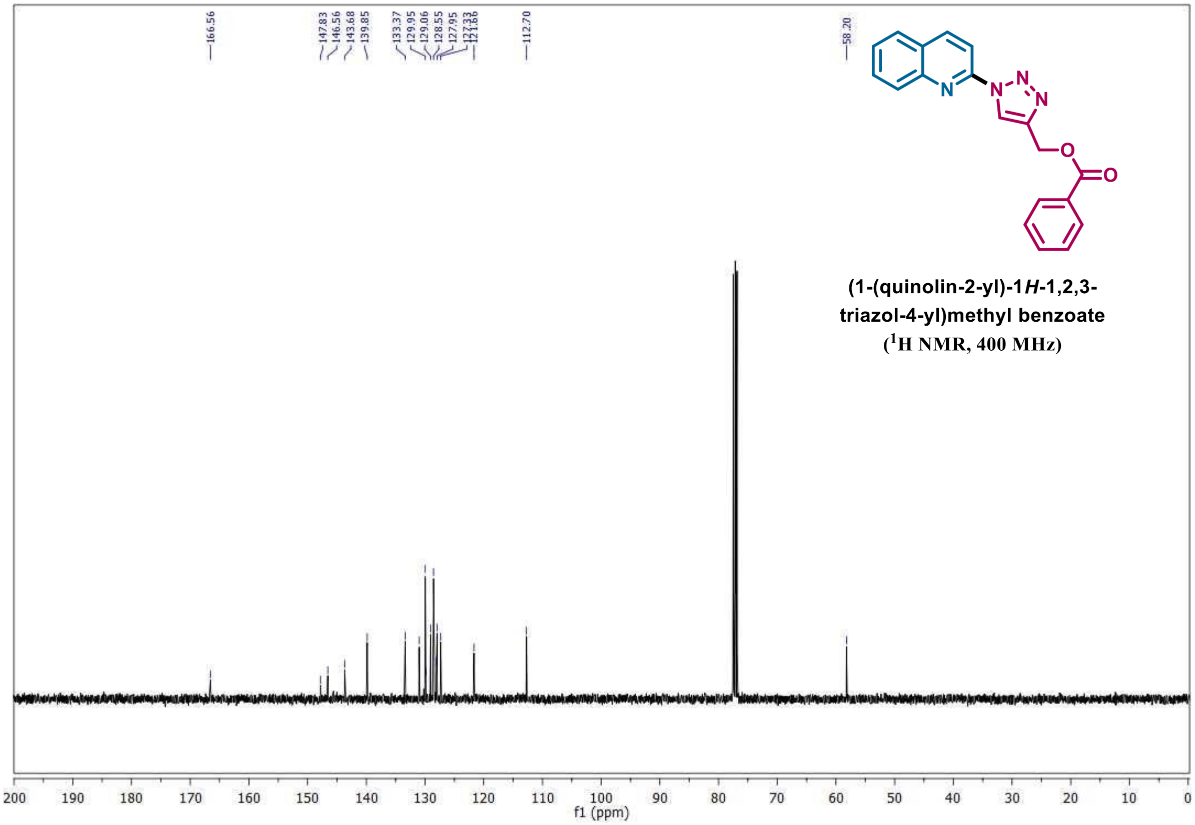

^{1}H and ^{13}C NMR Spectra of 3r

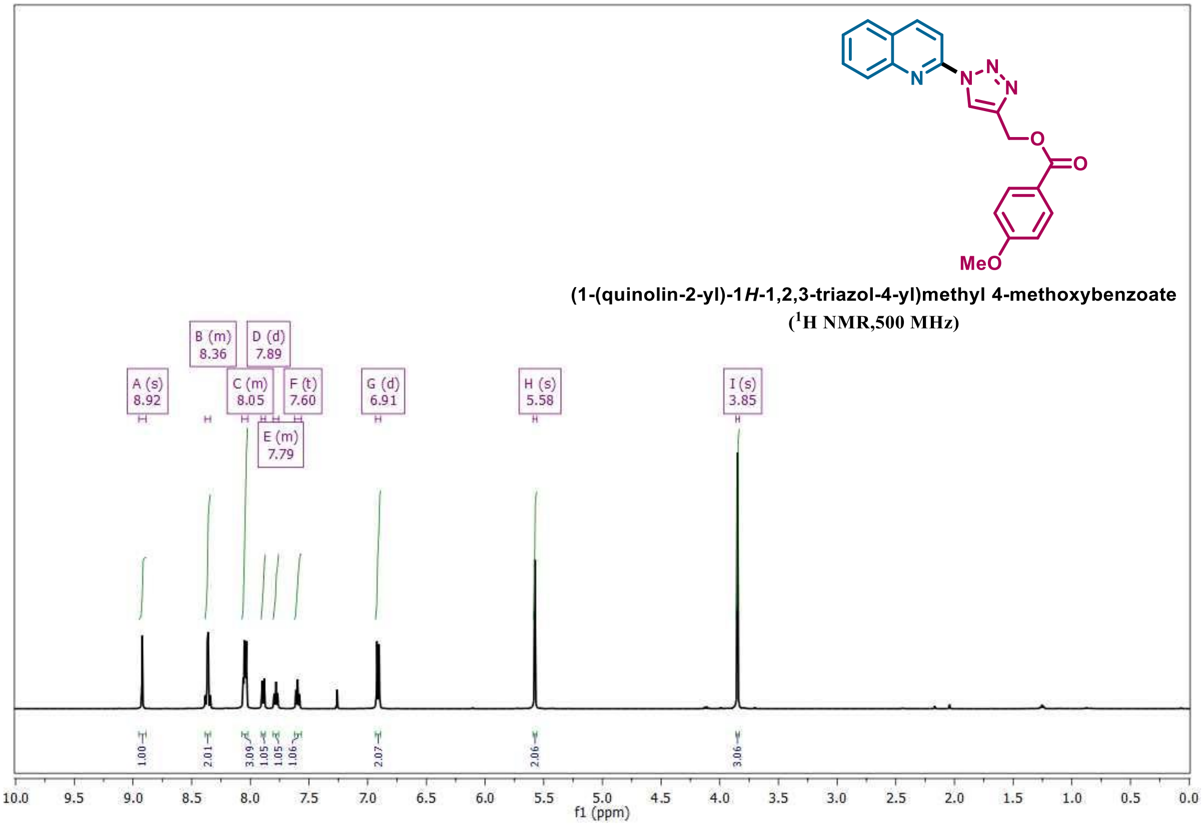

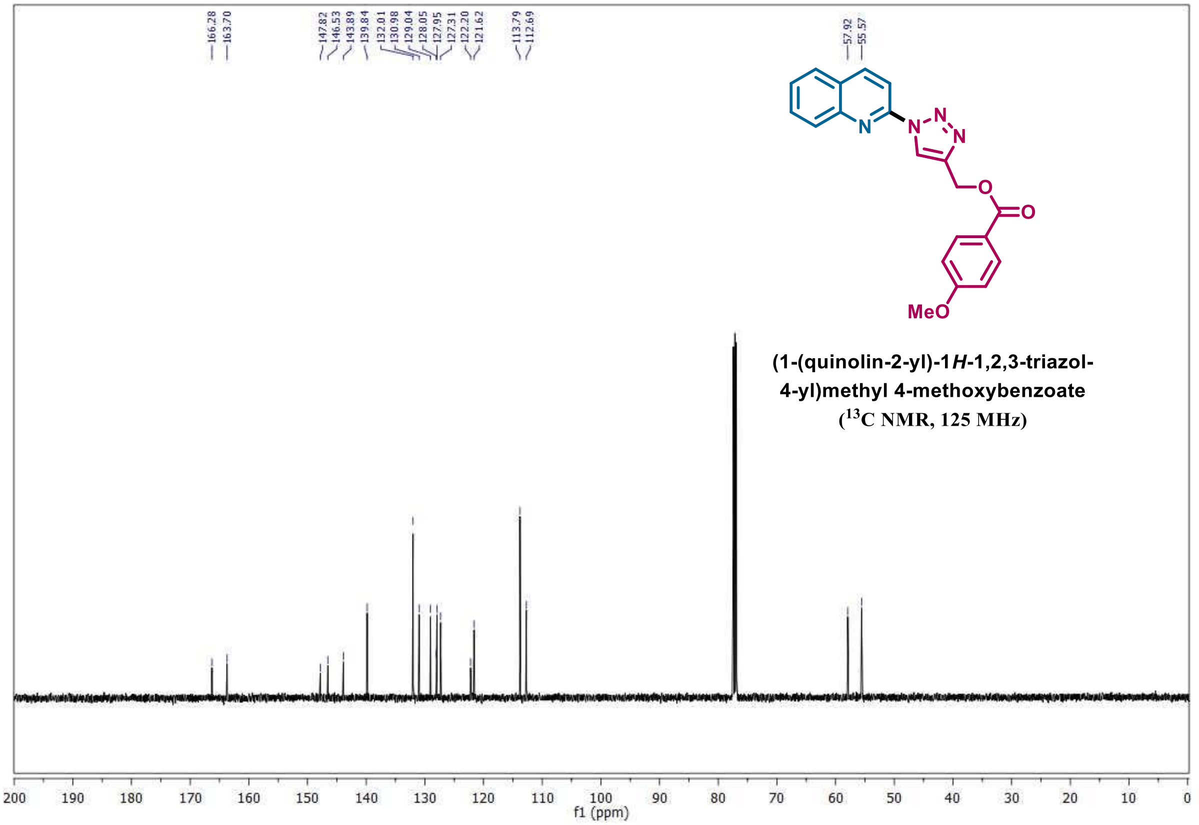

^{1}H and ^{13}C NMR Spectra of 3s

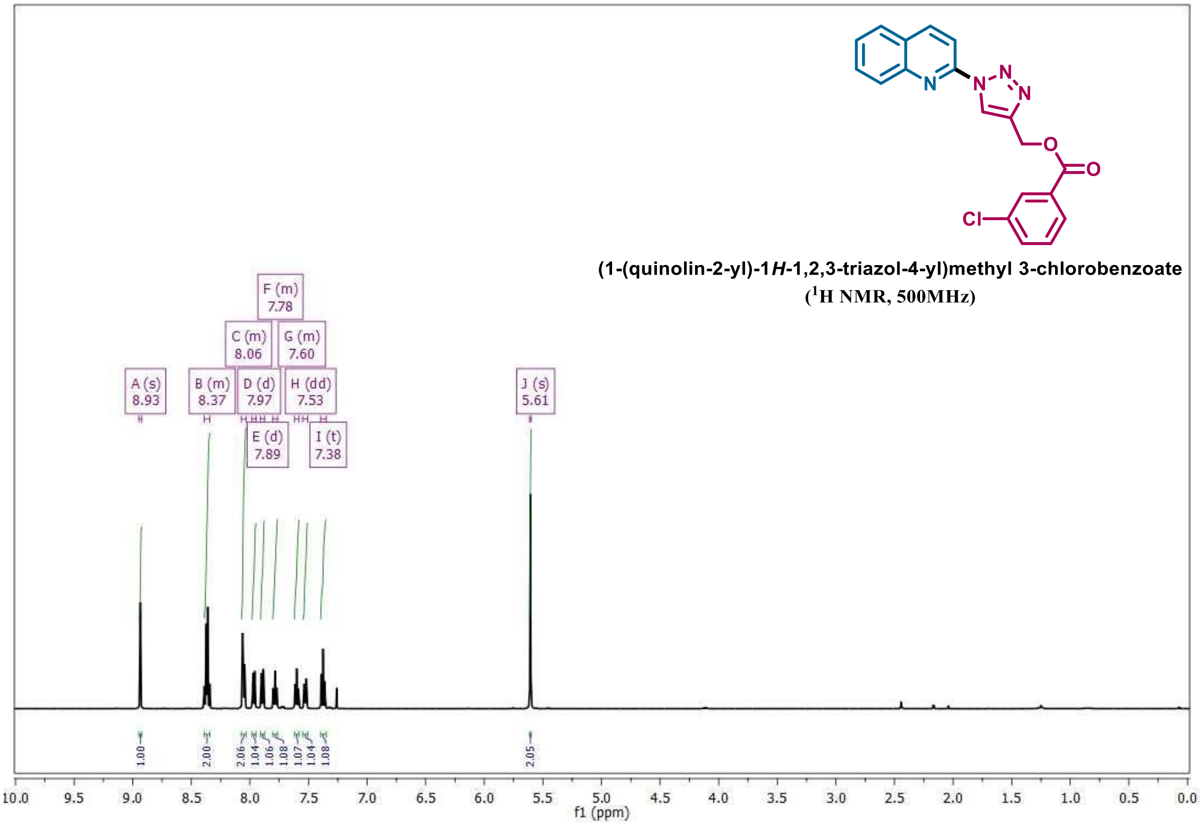

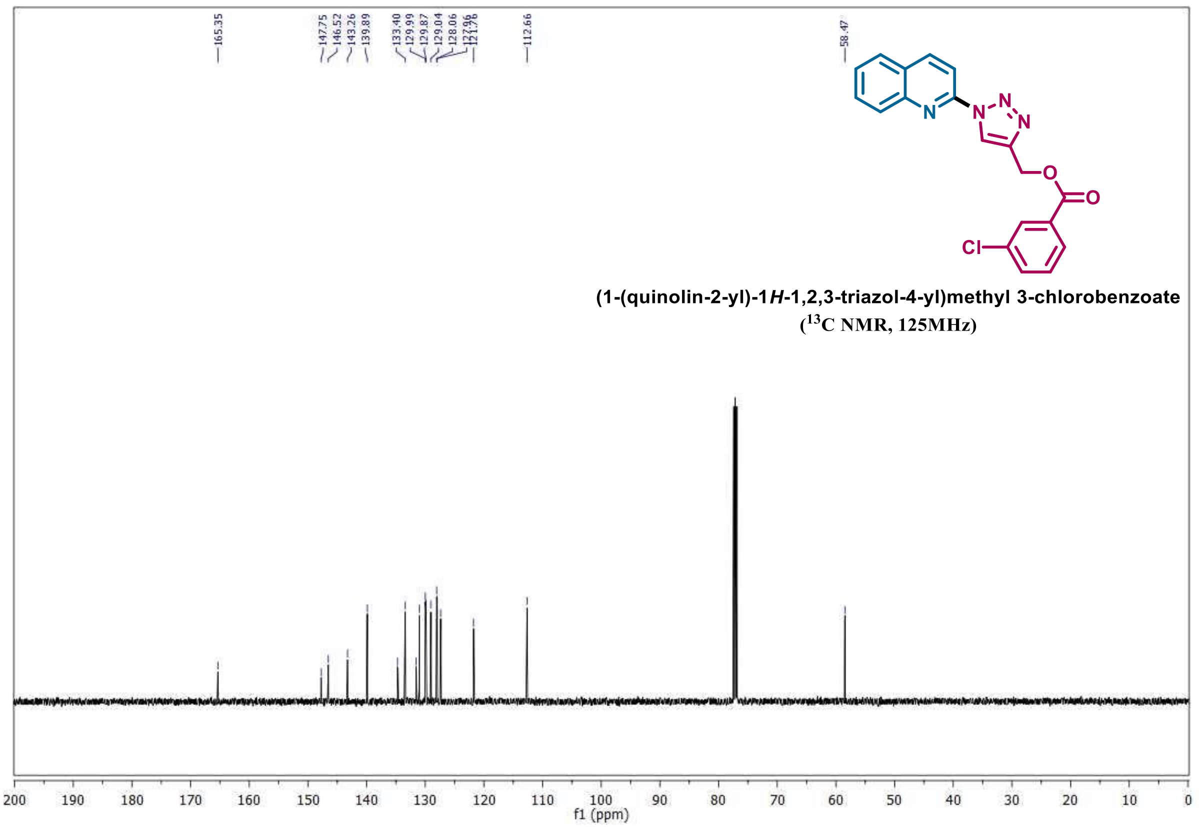

^{1}H and ^{13}C NMR Spectra of 3t

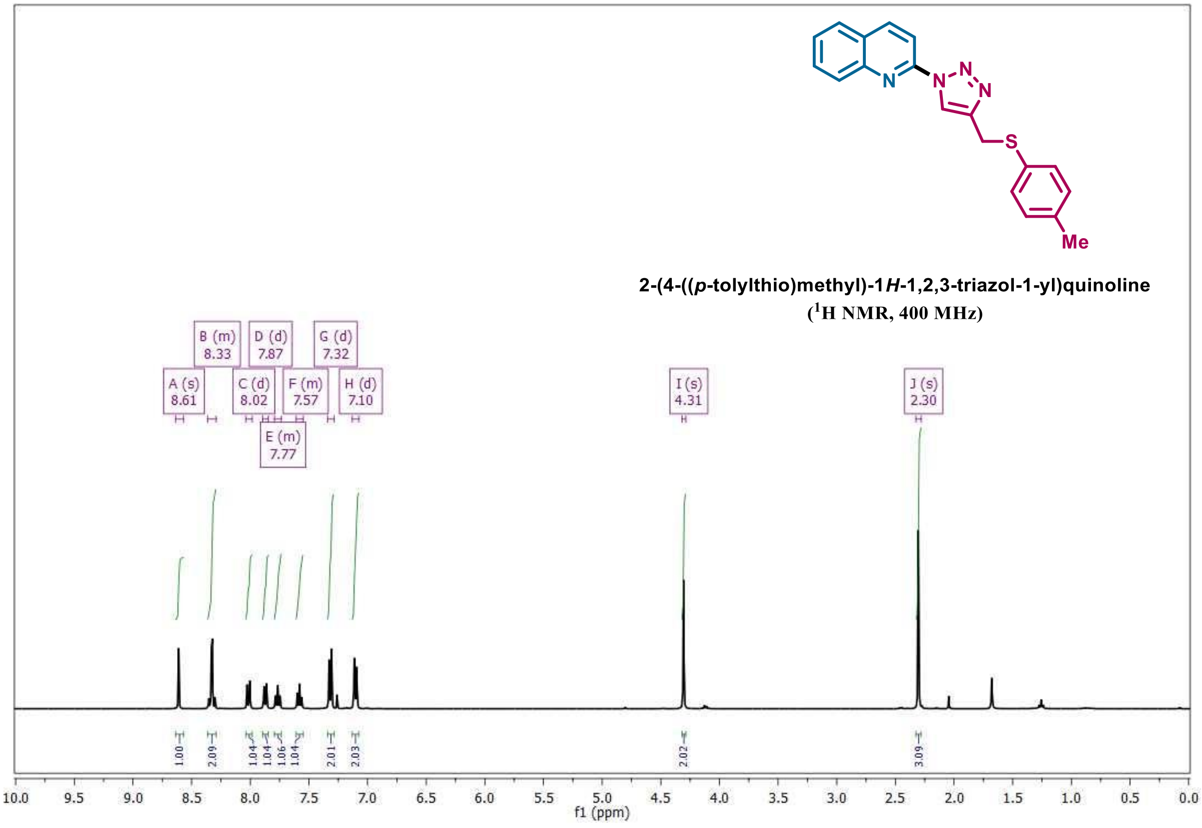

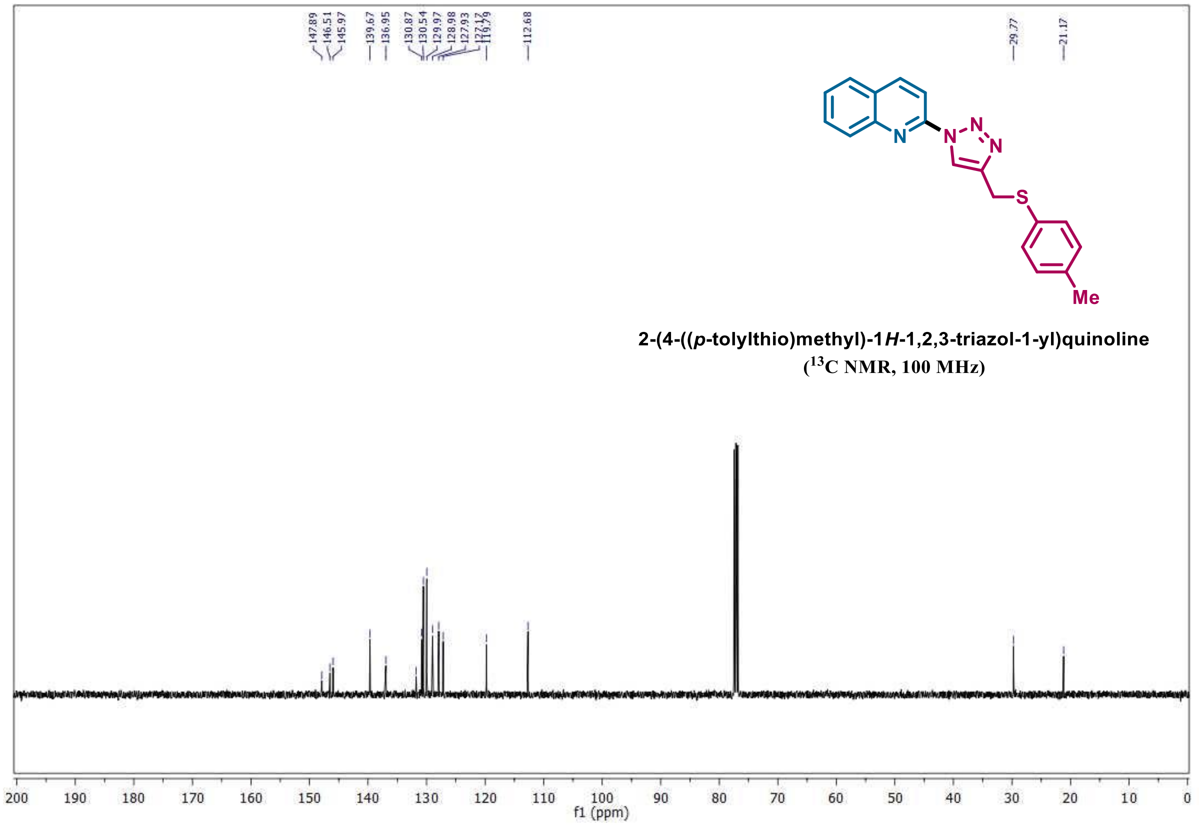

^{1}H and ^{13}C NMR Spectra of 3u

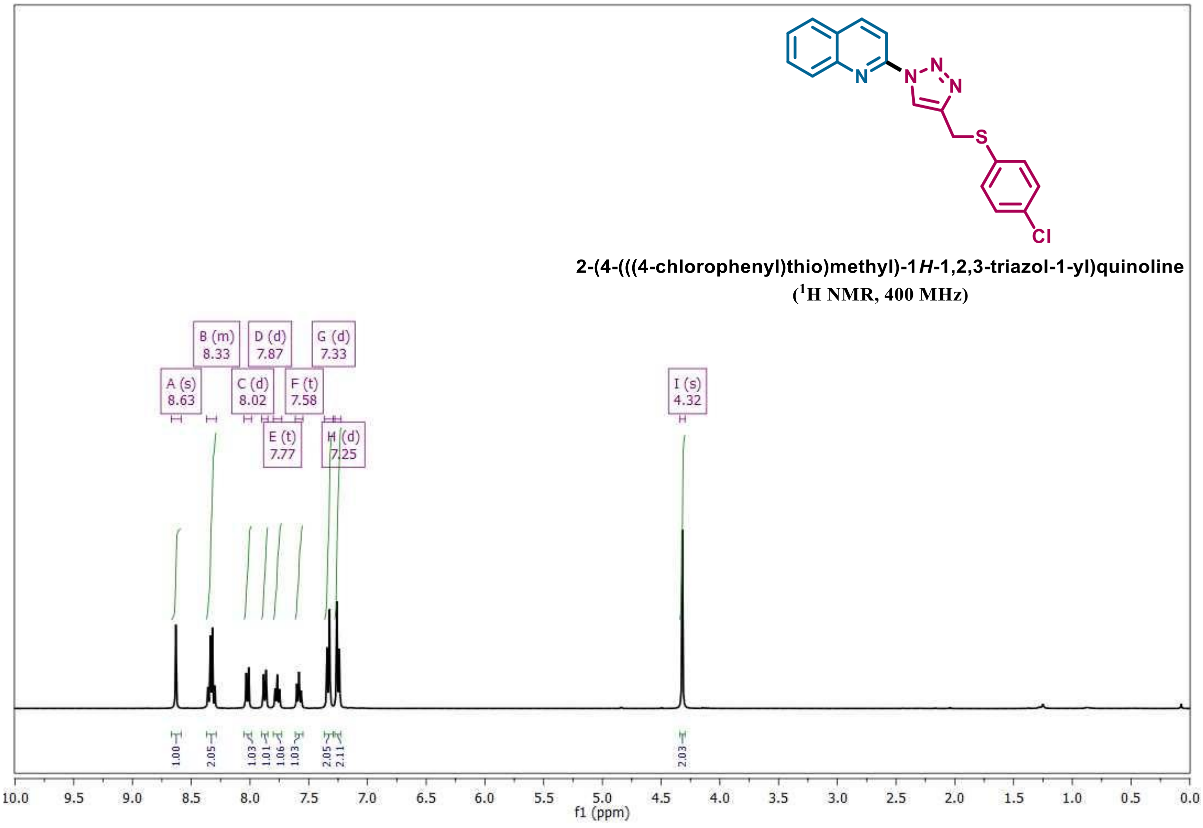

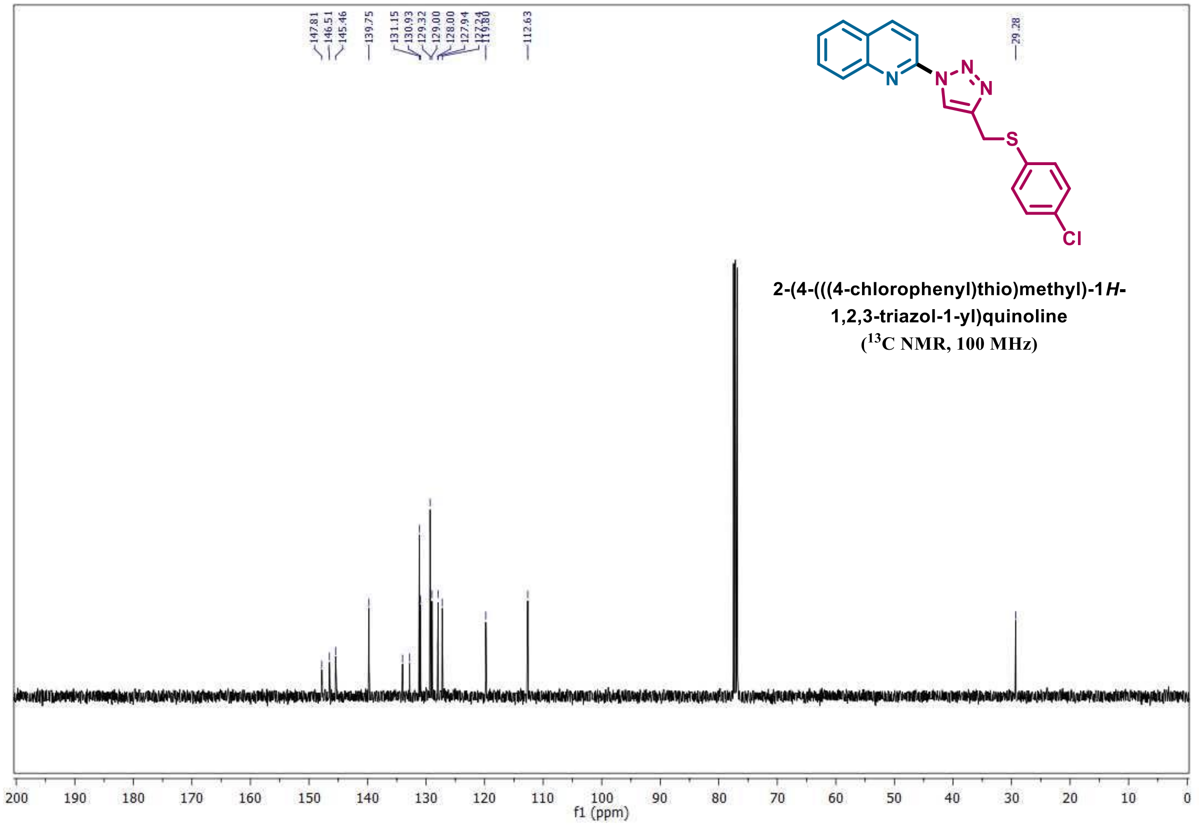

^{1}H and ^{13}C NMR Spectra of 3v

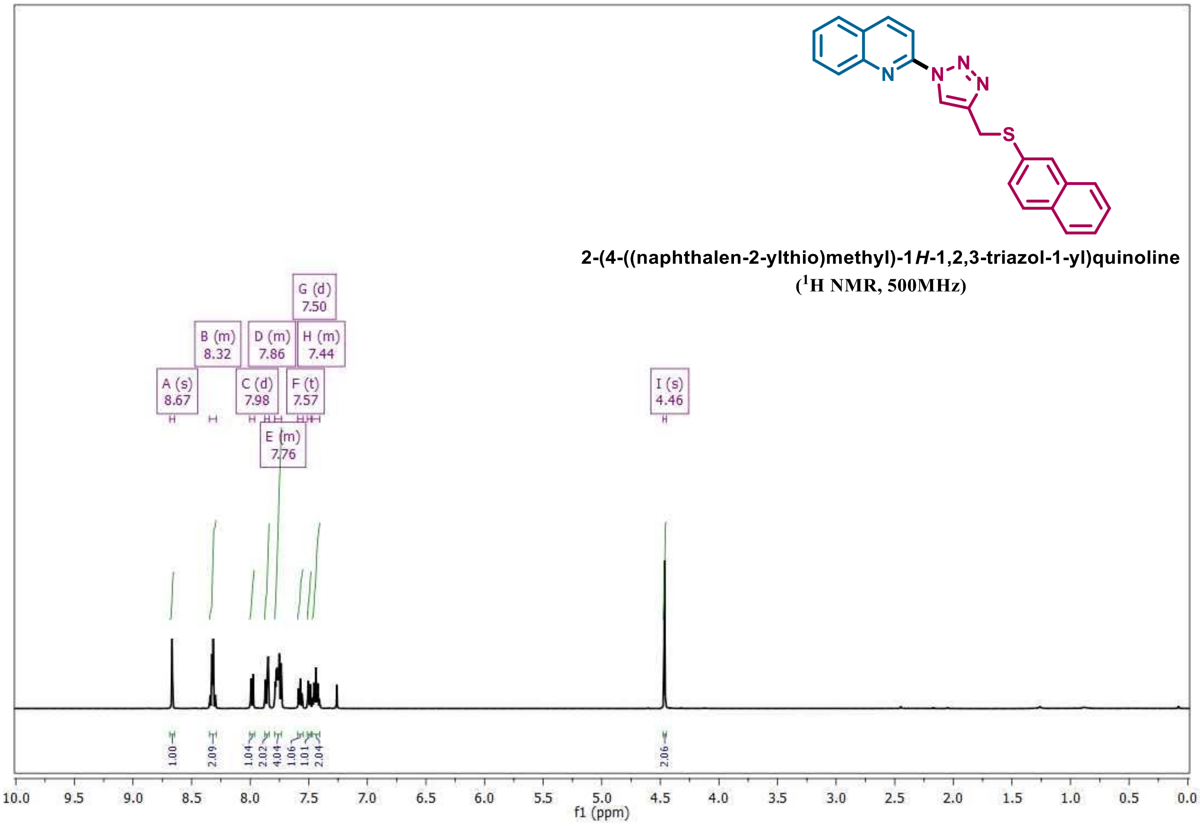

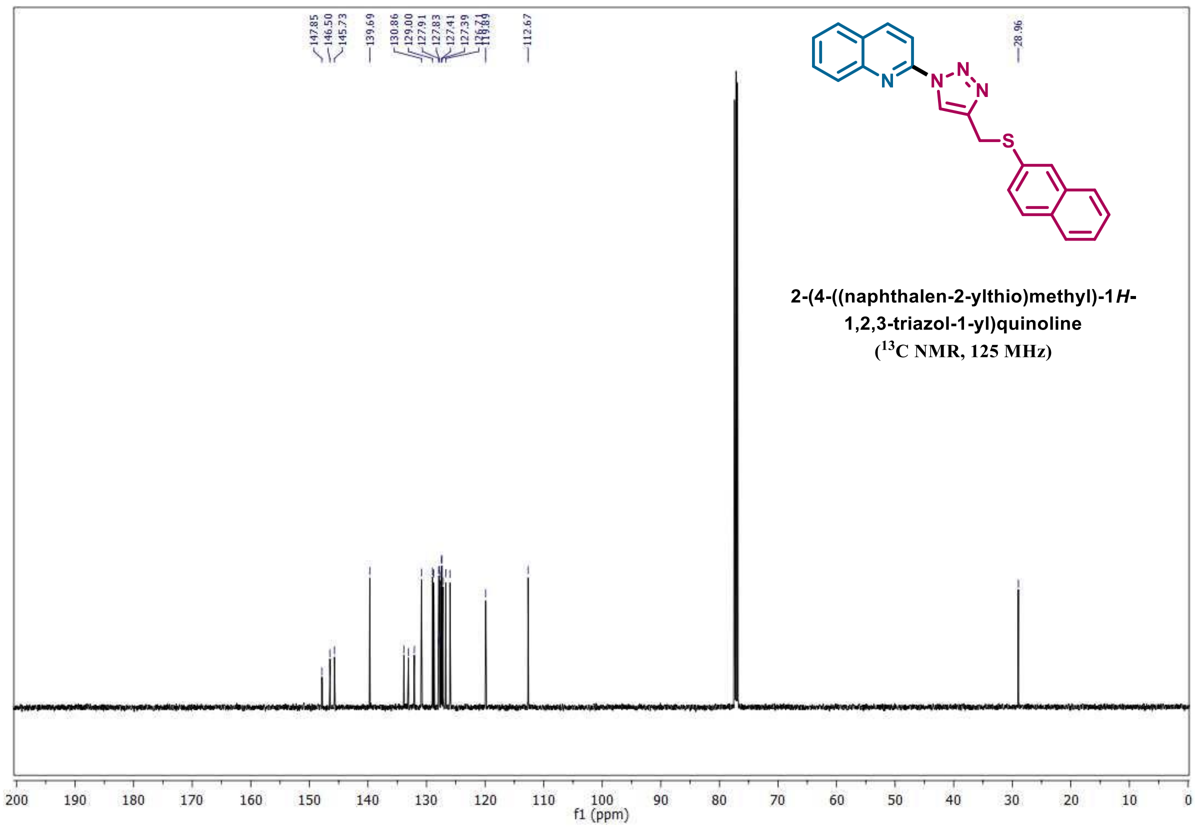

^{1}H and ^{13}C NMR Spectra of 3w

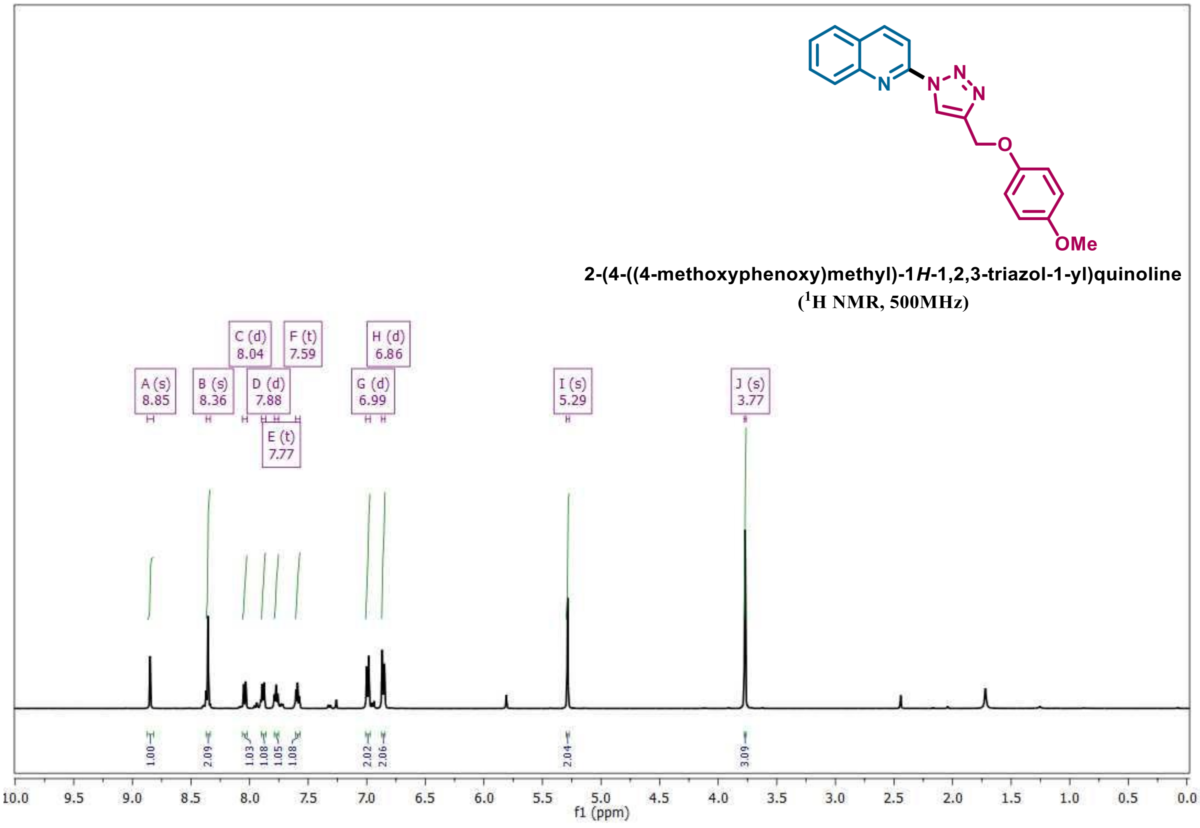

2-(4-((4-methoxyphenoxy)methyl)-1*H*-1,2,3-triazol-1-yl)quinoline
(^{1}H NMR, 500MHz)

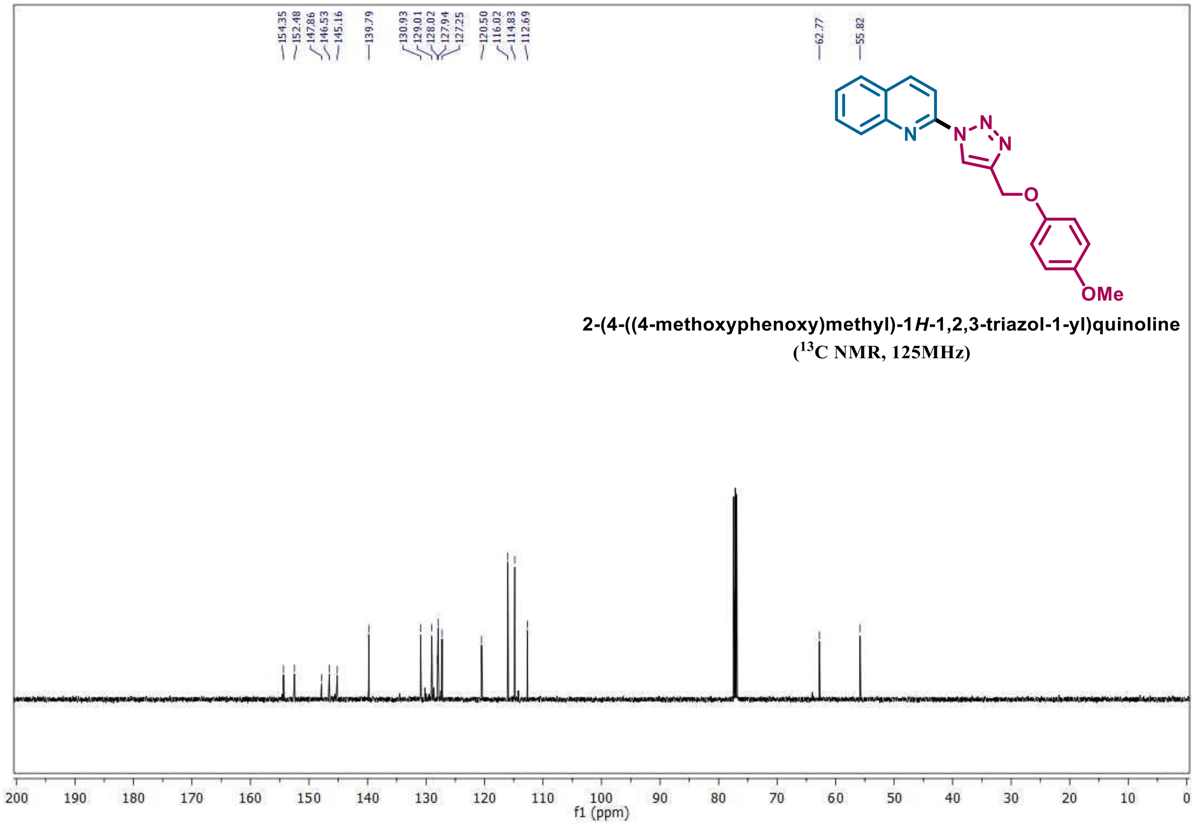

2-(4-((4-methoxyphenoxy)methyl)-1*H*-1,2,3-triazol-1-yl)quinoline
(^{13}C NMR, 125MHz)

^{1}H and ^{13}C NMR Spectra of 3x

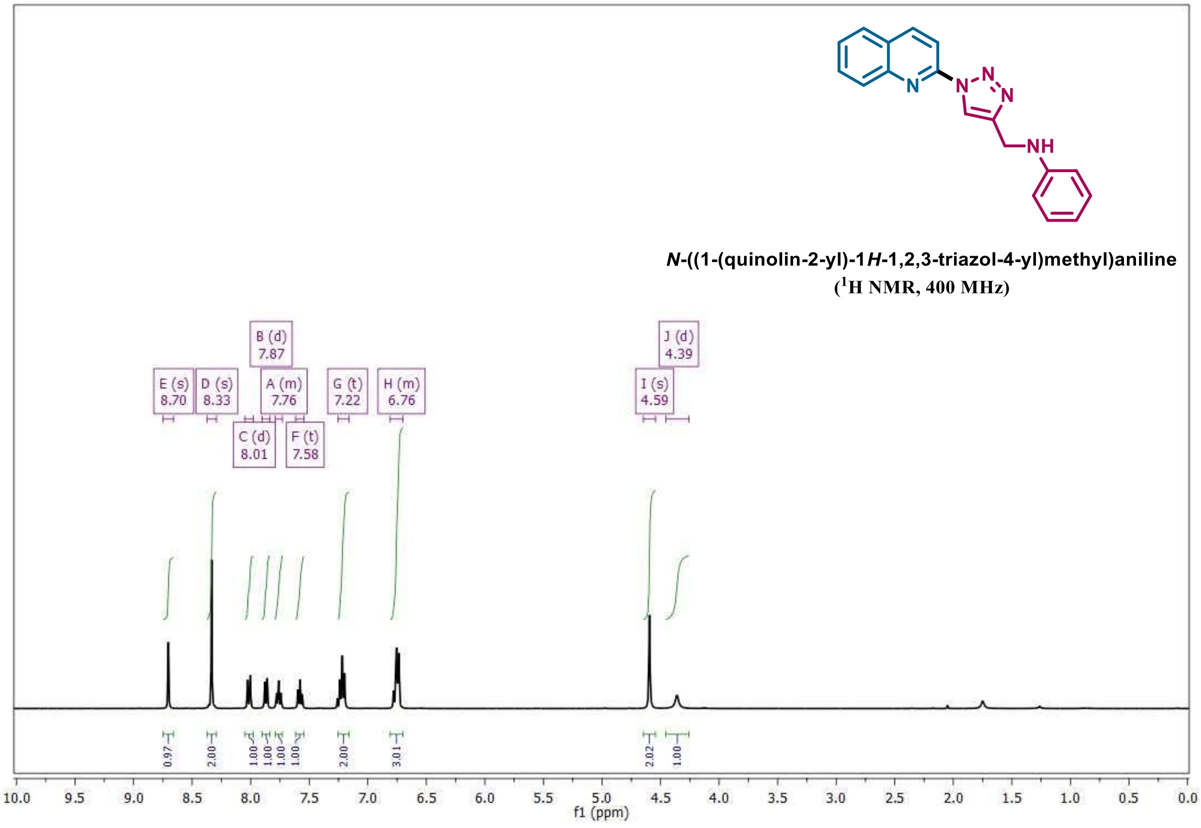

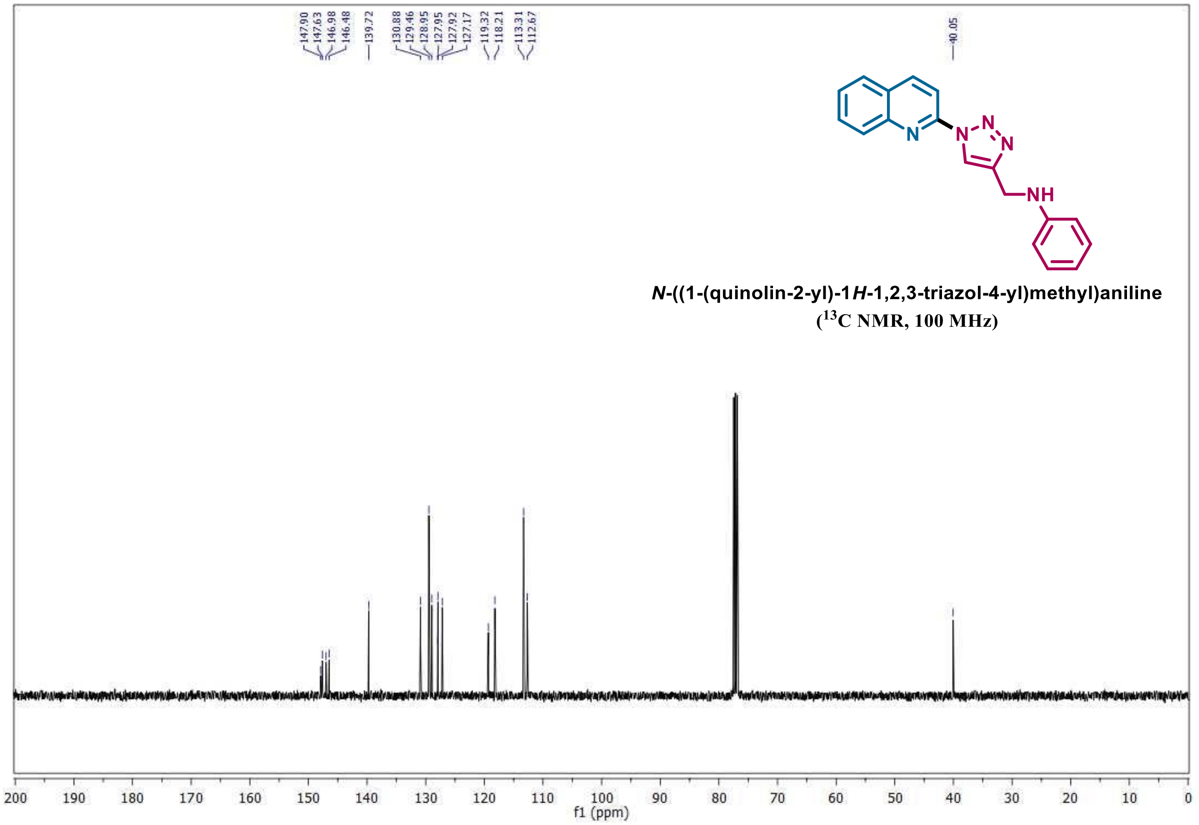

^{1}H and ^{13}C NMR Spectra of 3y

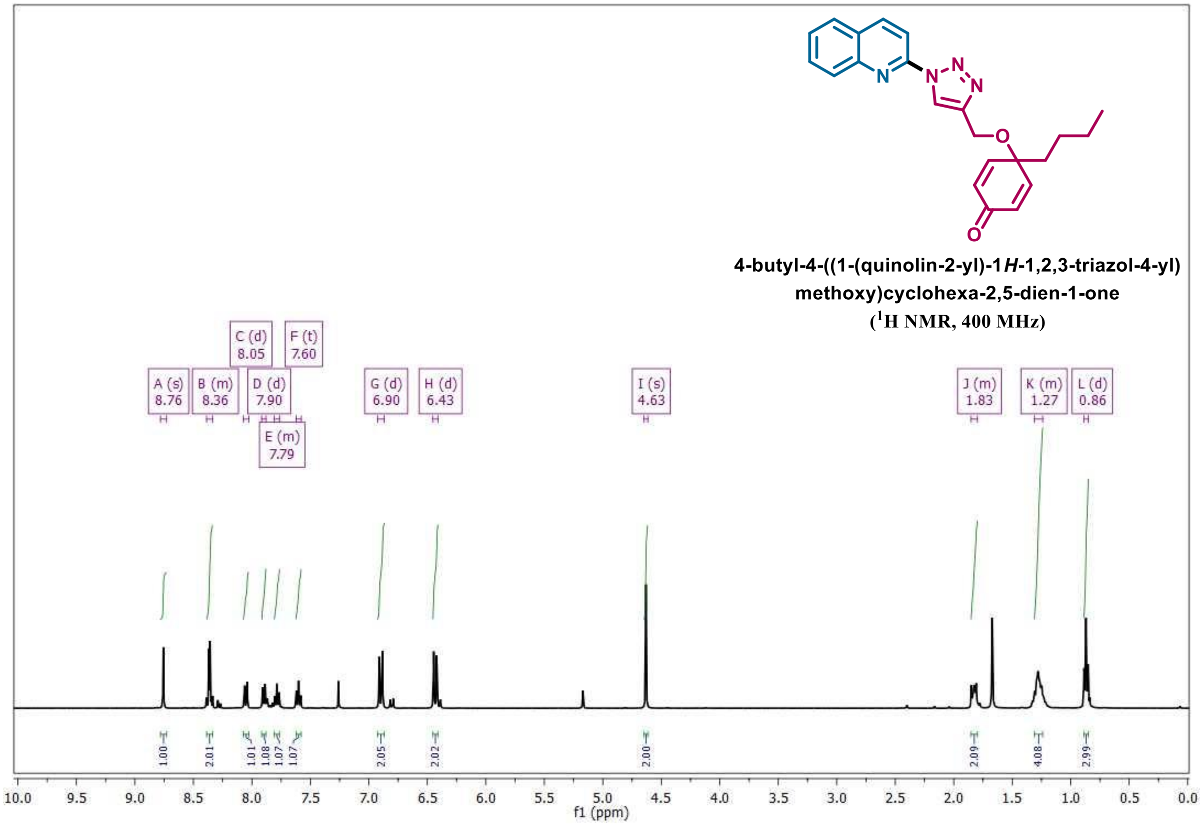

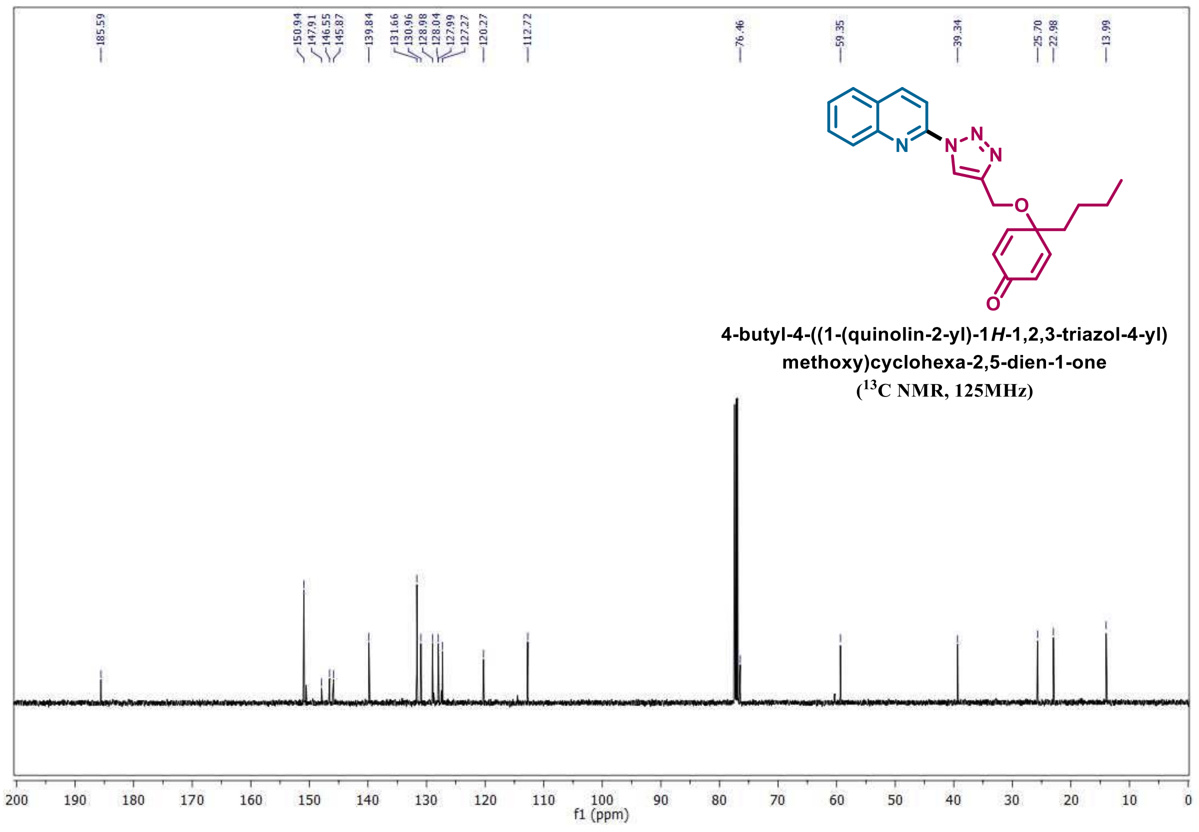

^{1}H and ^{13}C NMR Spectra of 3z

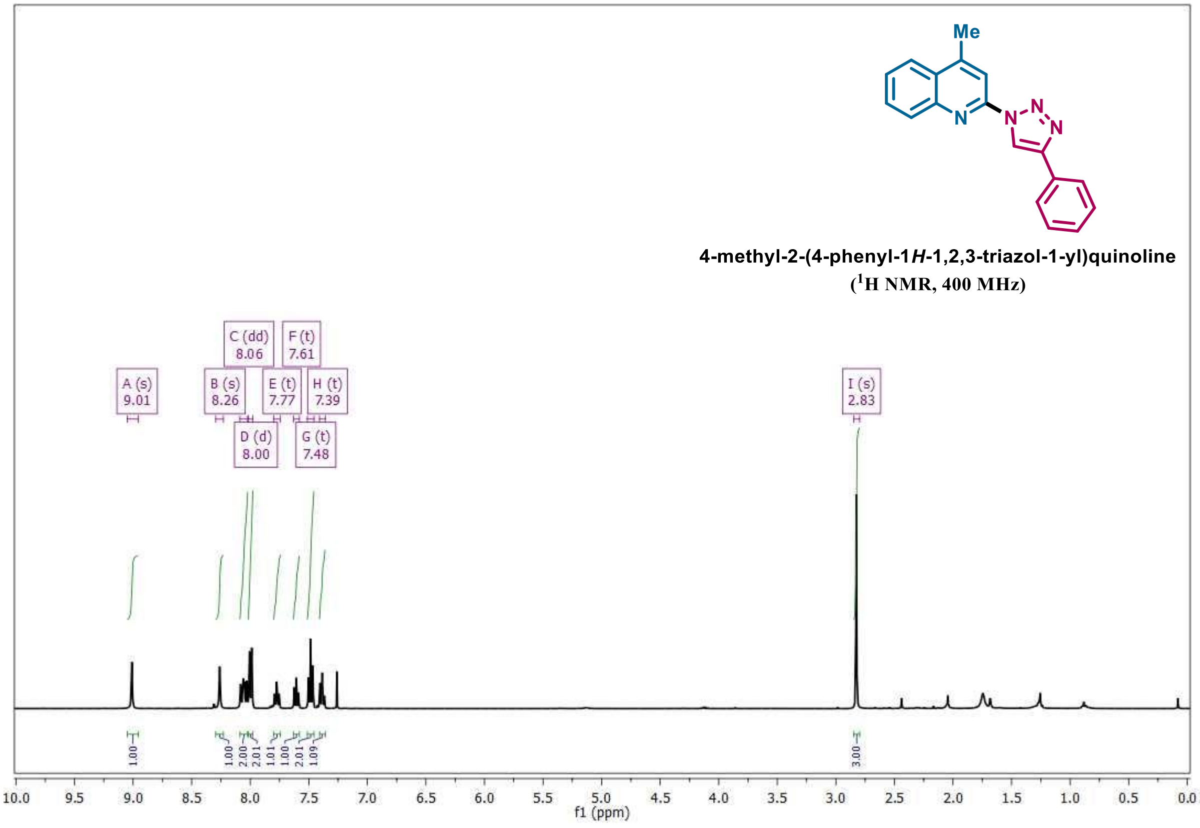

4-methyl-2-(4-phenyl-1*H*-1,2,3-triazol-1-yl)quinoline
(^{1}H NMR, 400 MHz)

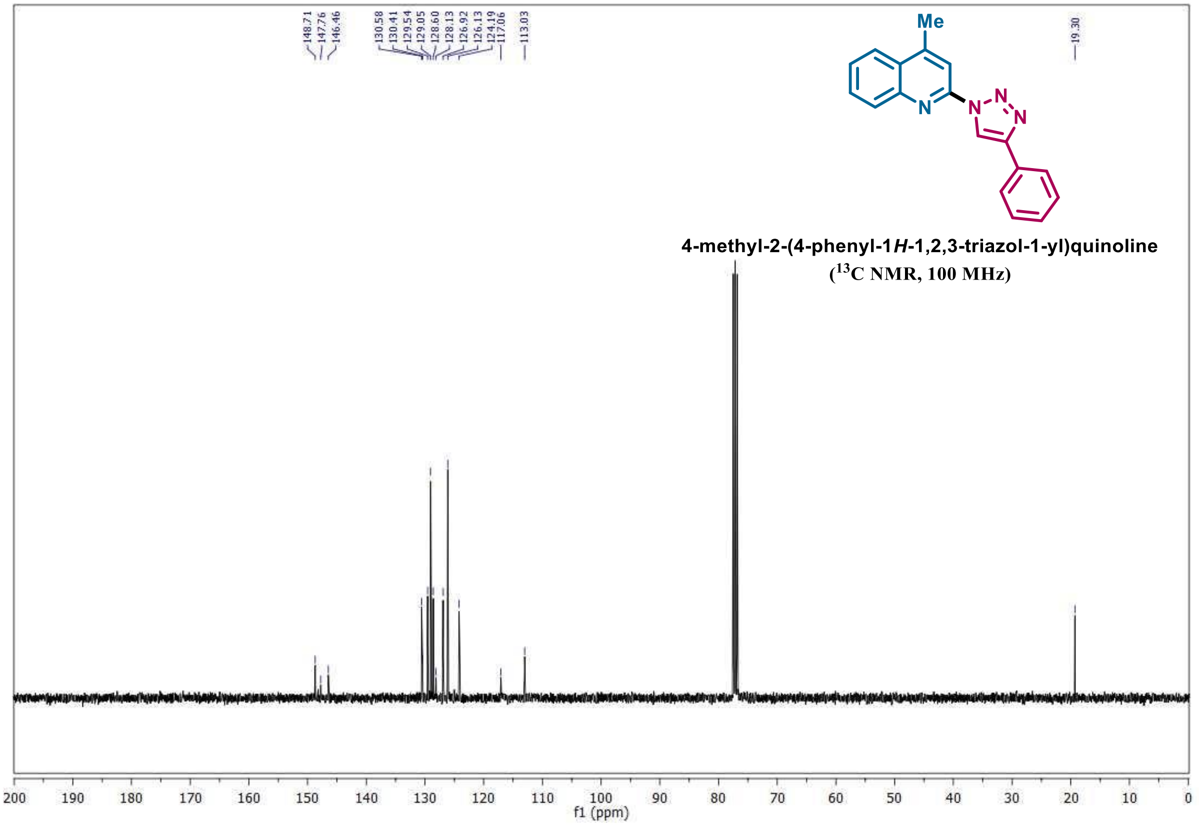

4-methyl-2-(4-phenyl-1*H*-1,2,3-triazol-1-yl)quinoline
(^{13}C NMR, 100 MHz)

^{1}H and ^{13}C NMR Spectra of 3aa

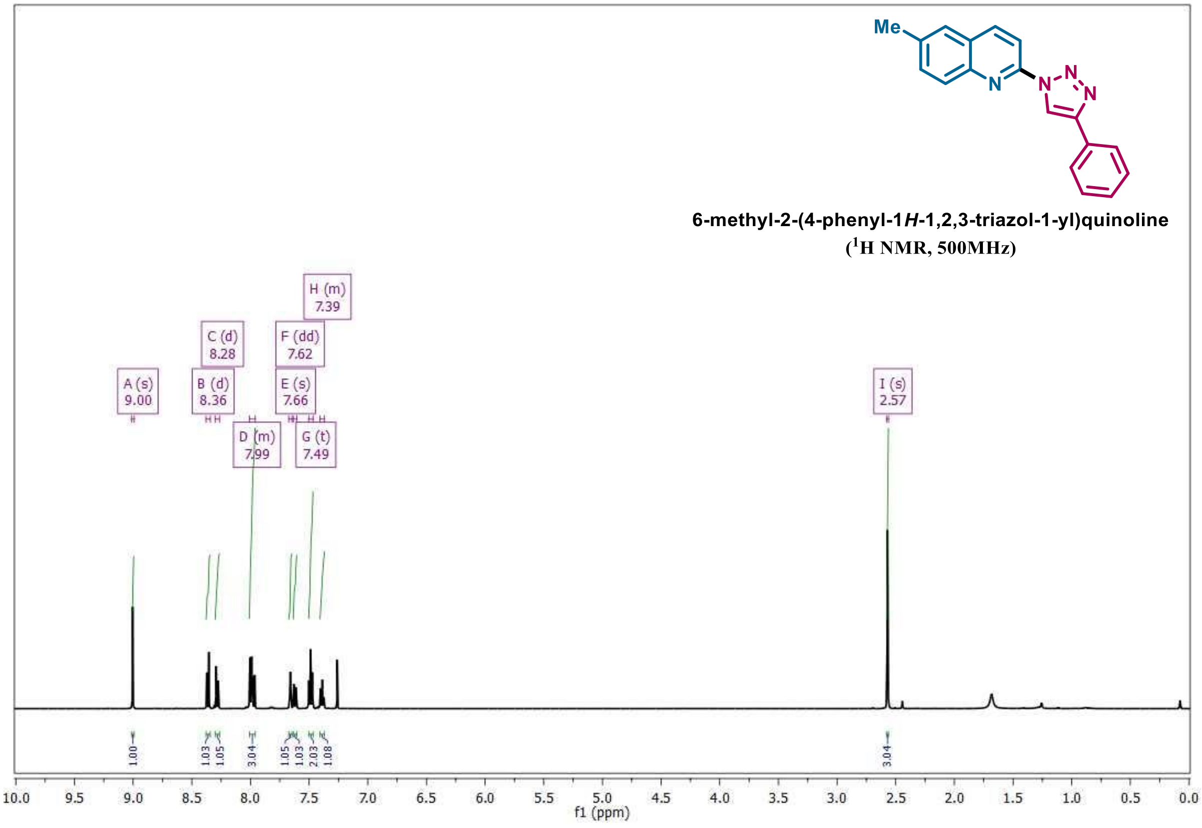

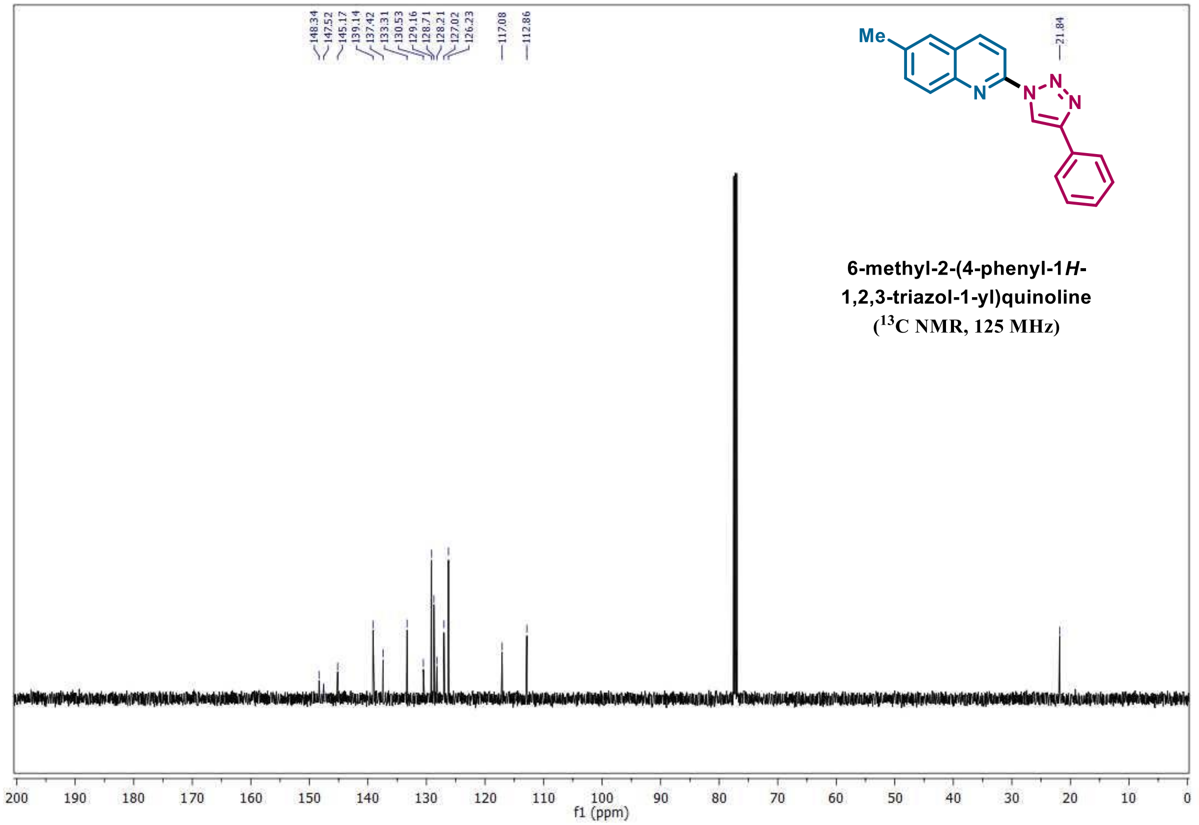

^{1}H and ^{13}C NMR Spectra of 3ab

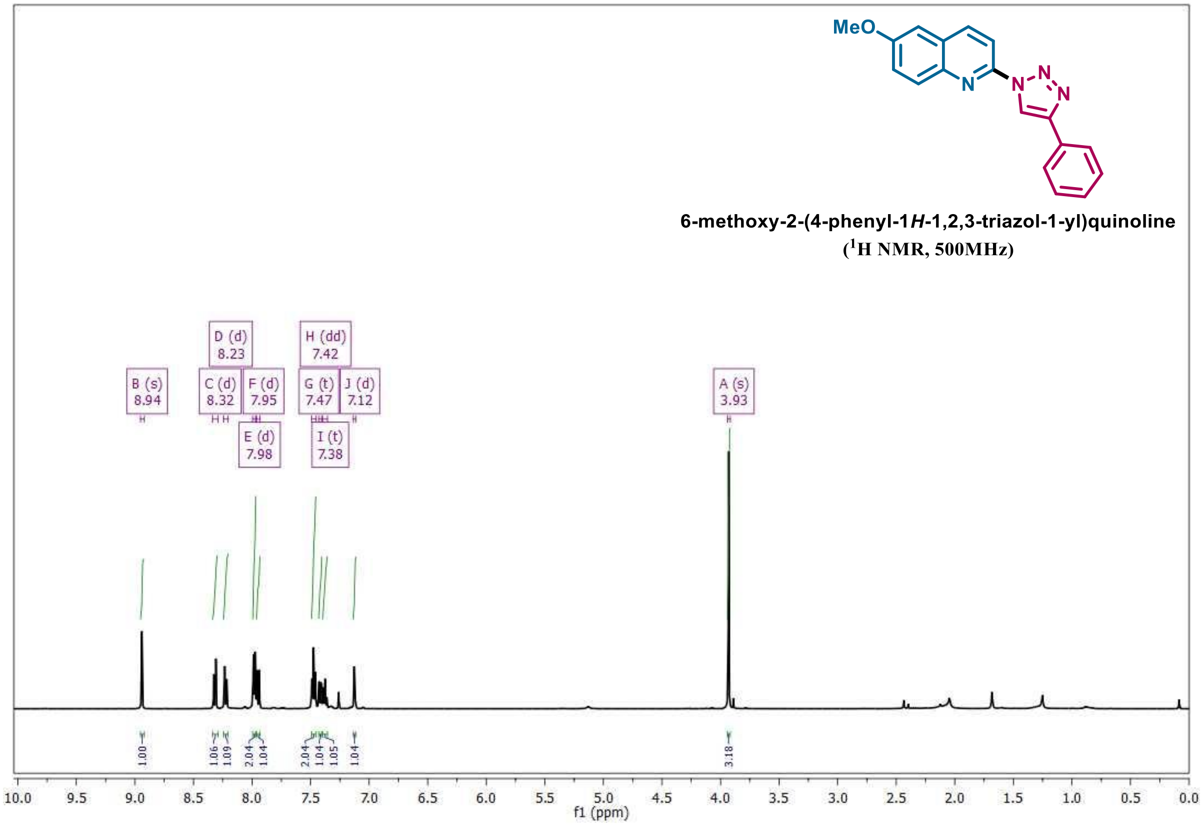

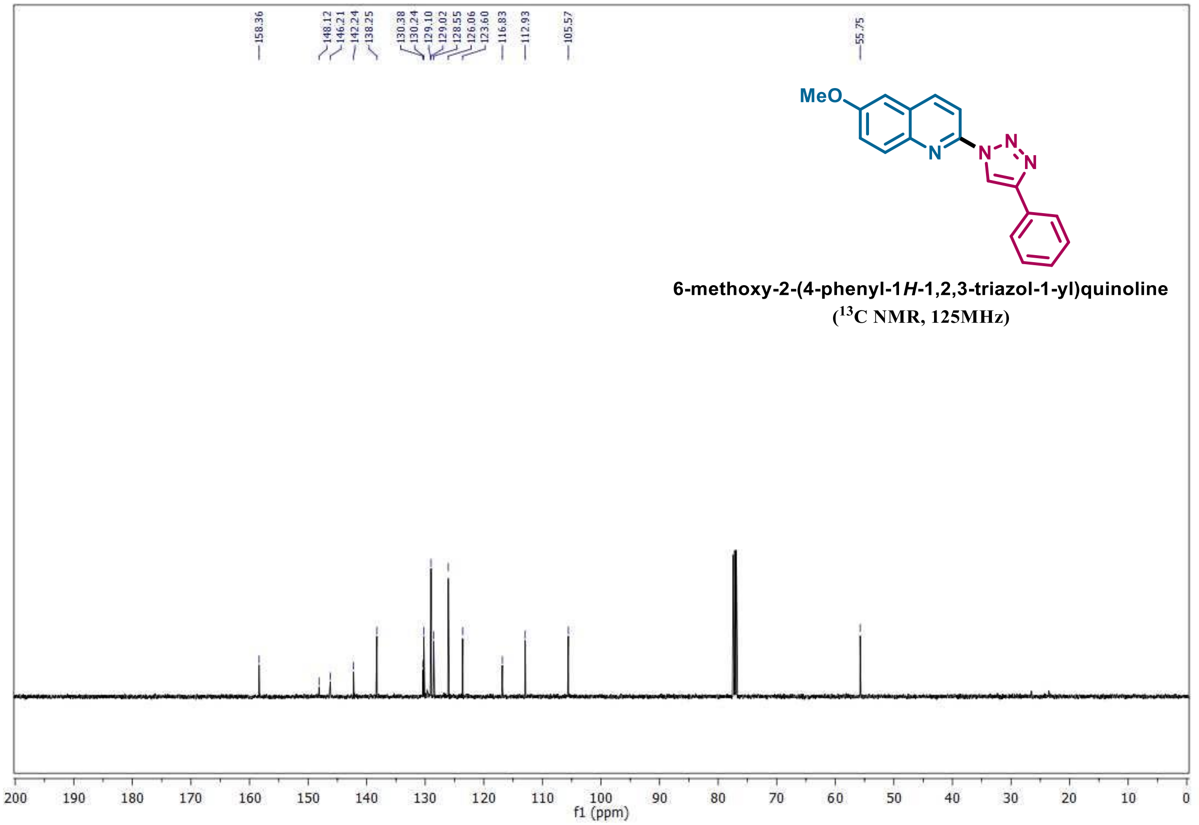

^{1}H and ^{13}C NMR Spectra of 3ac

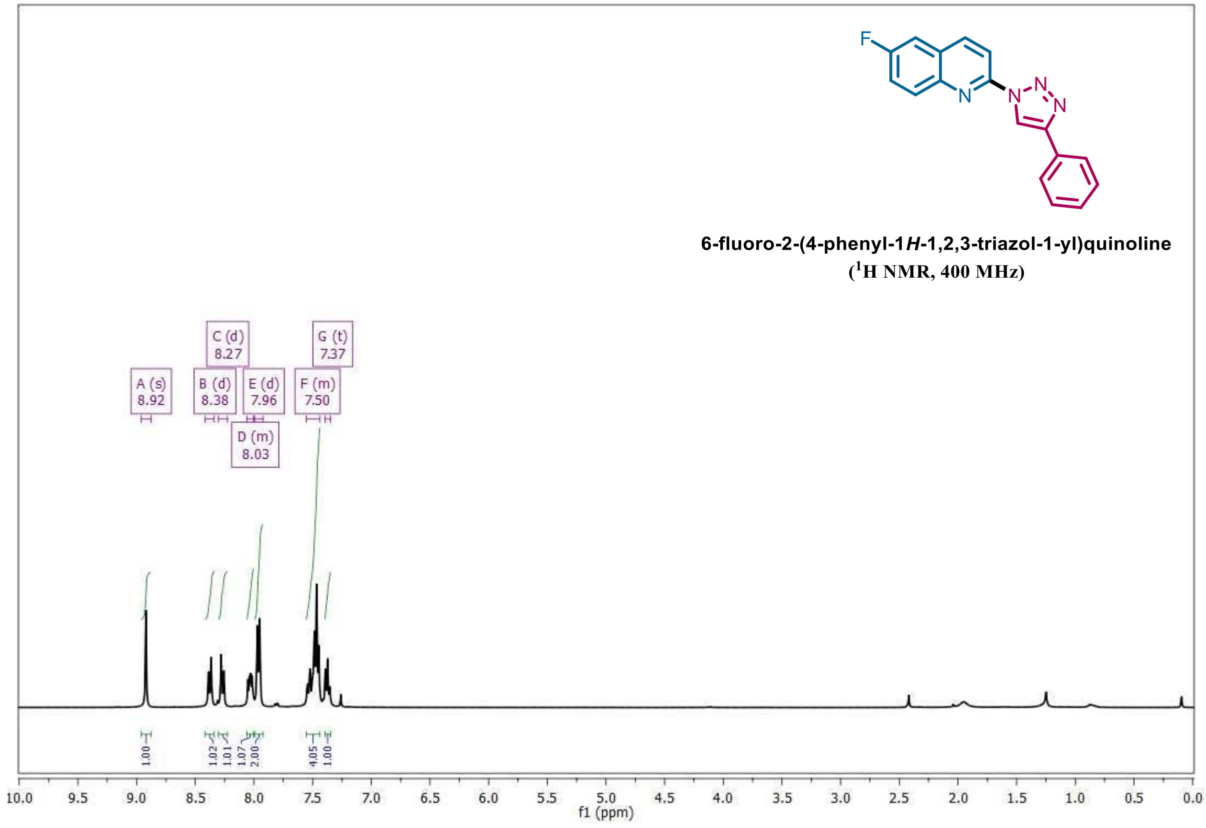

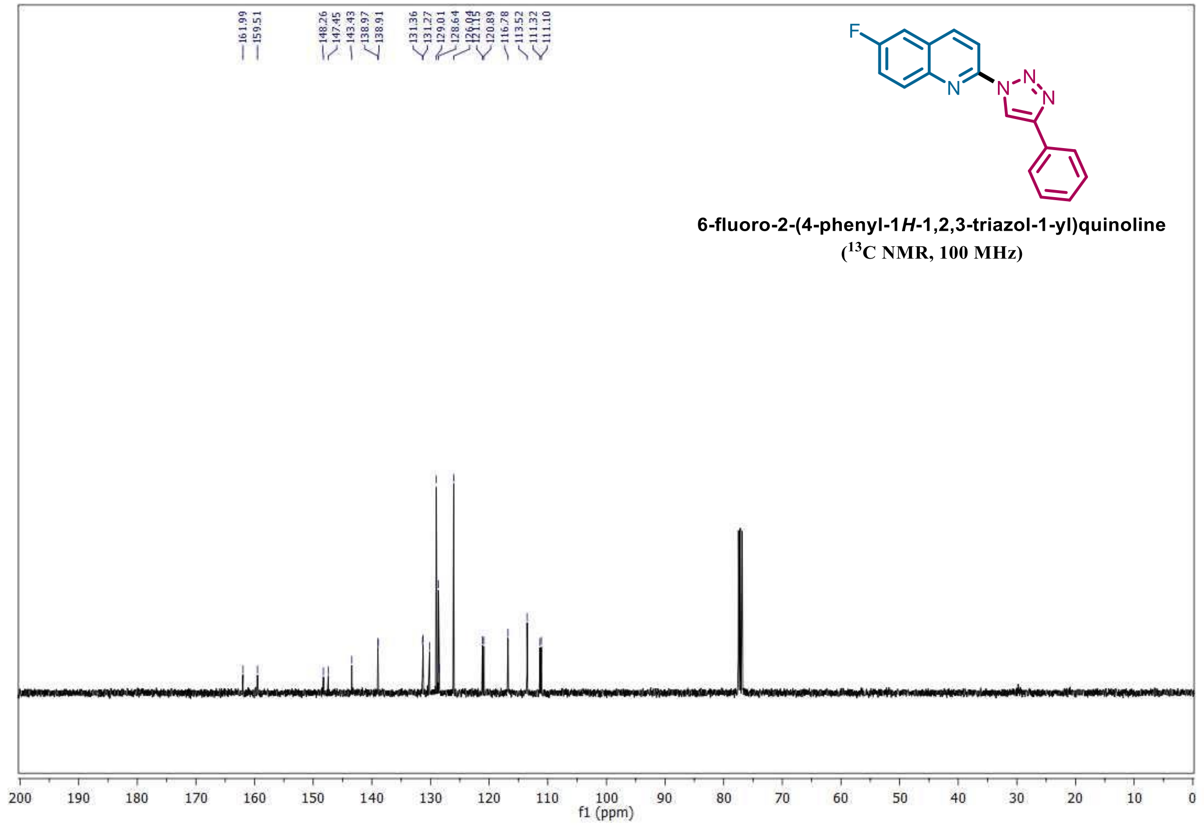

^{1}H and ^{13}C NMR Spectra of 3ad

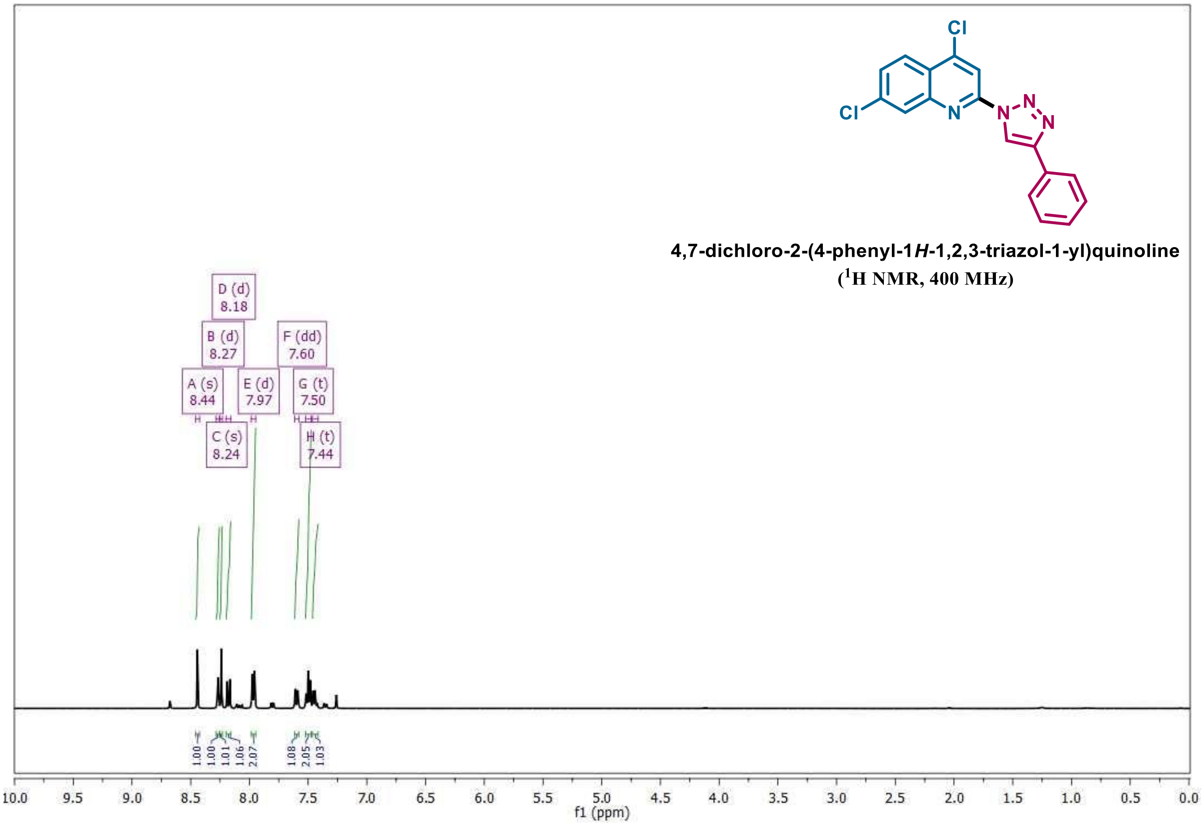

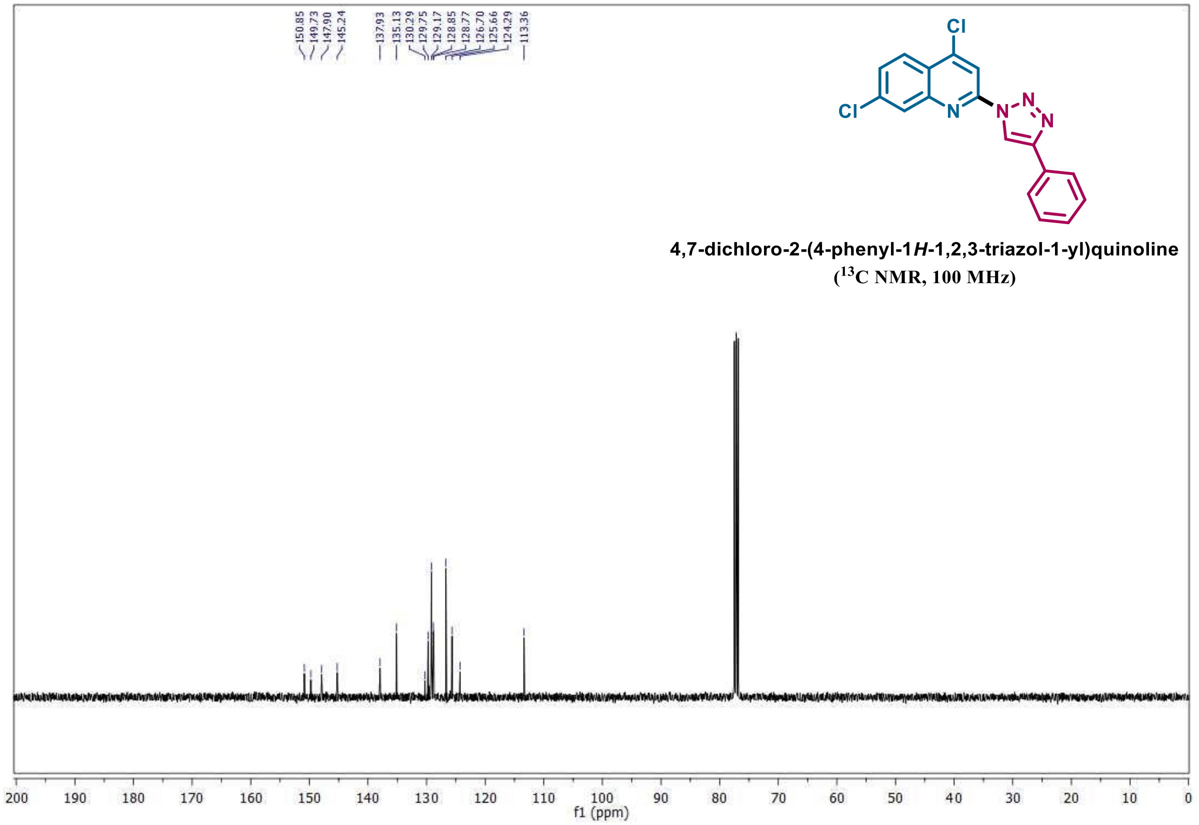

^{1}H and ^{13}C NMR Spectra of 3ae

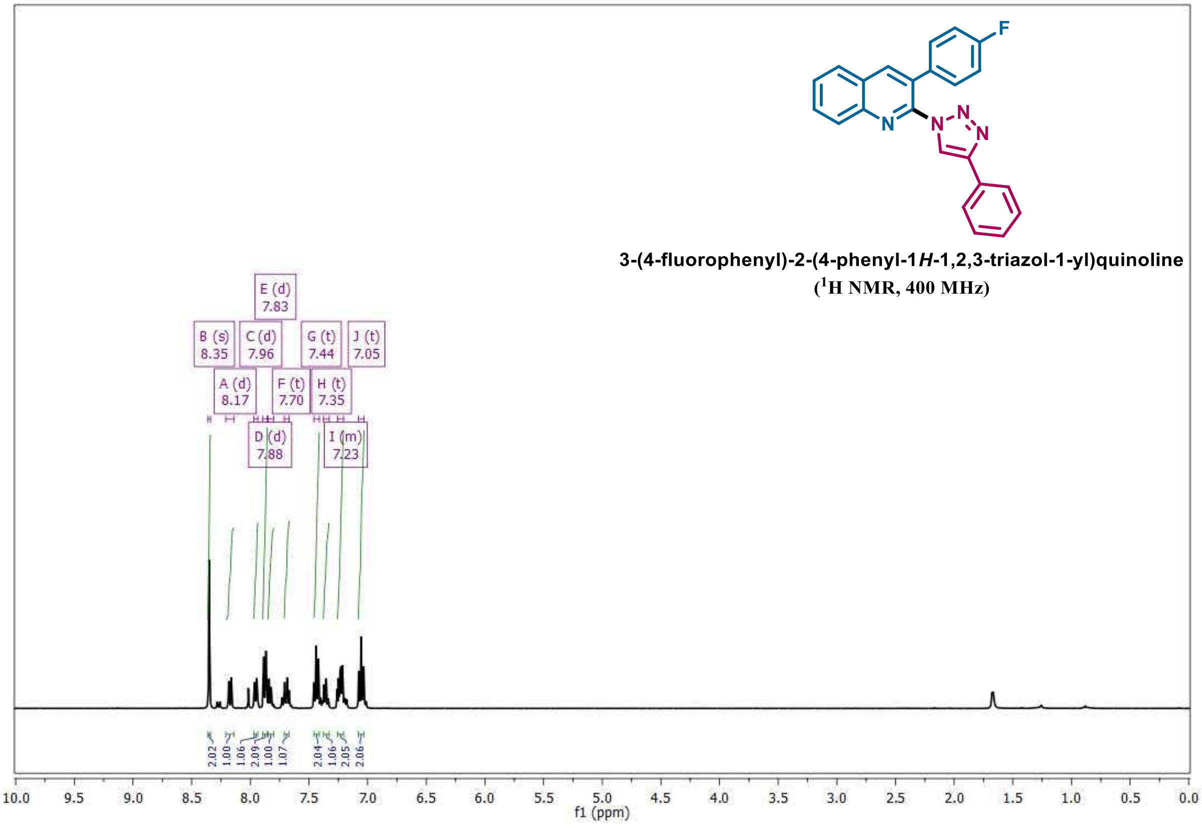

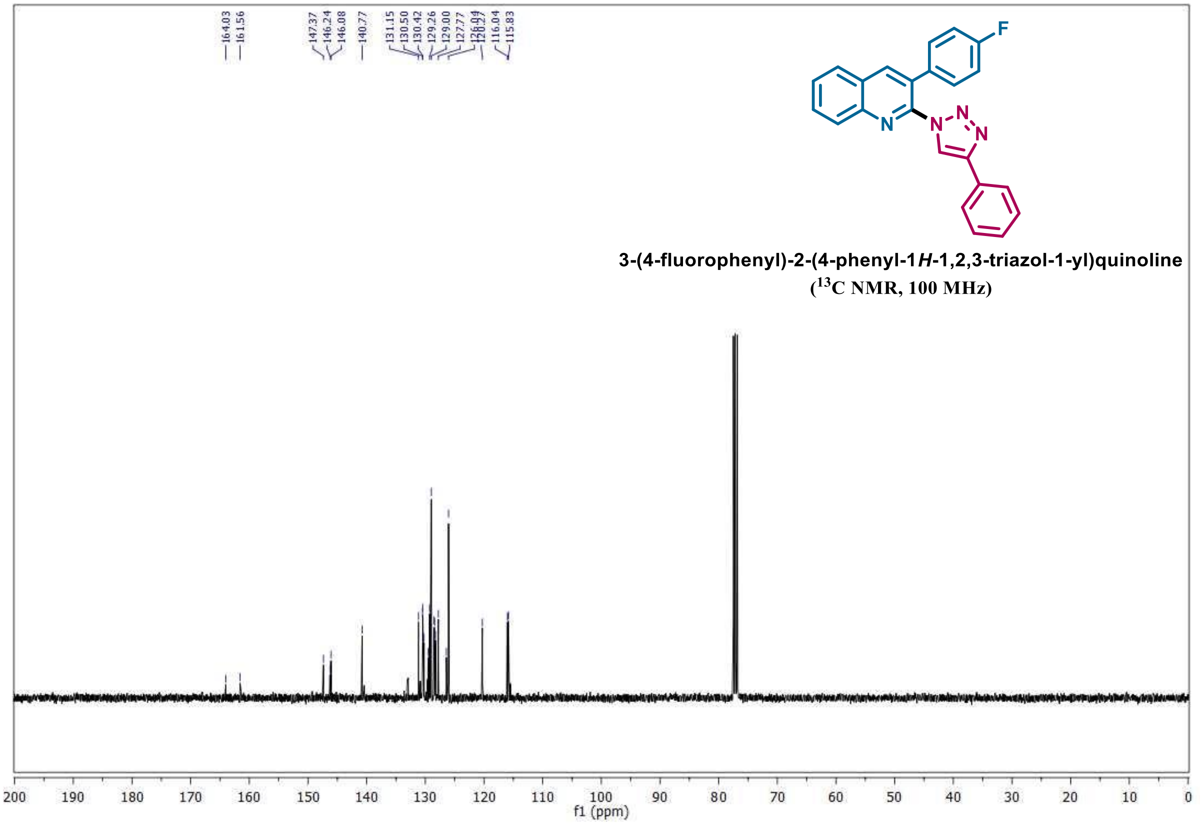

^{1}H and ^{13}C NMR Spectra of 3af

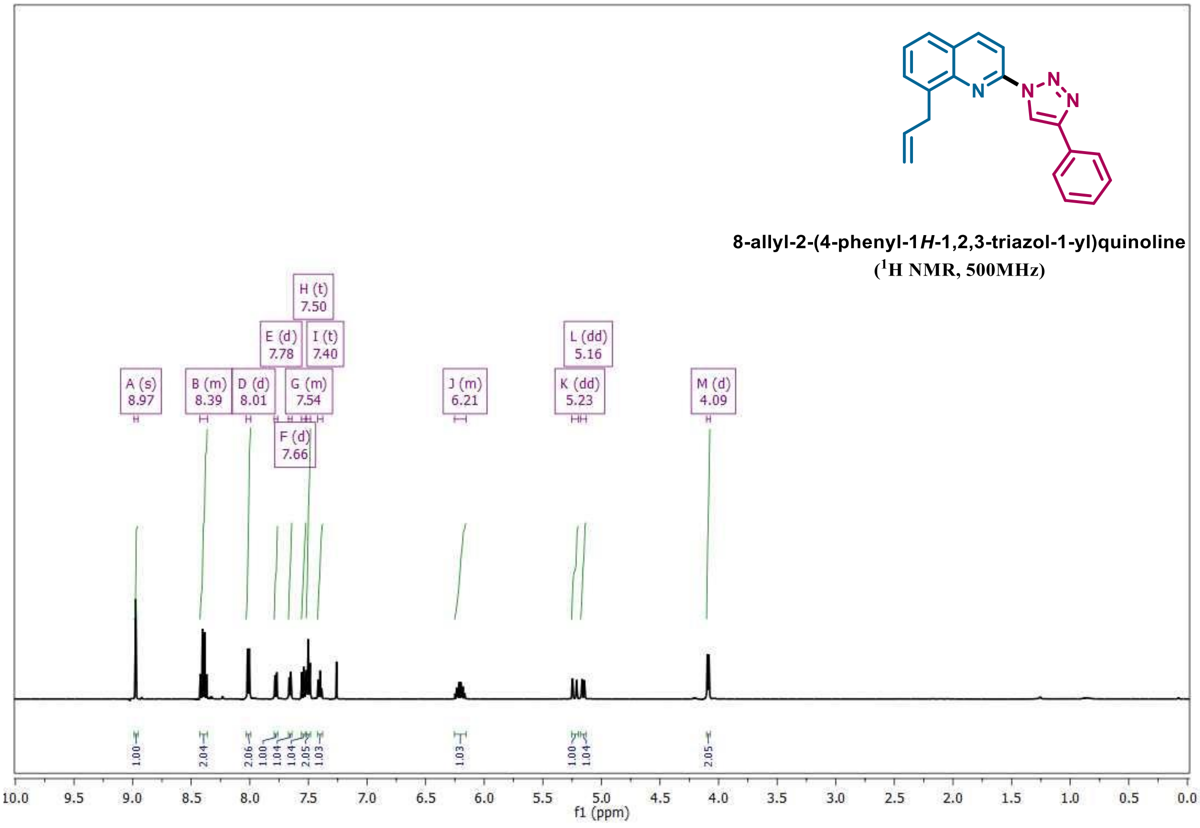

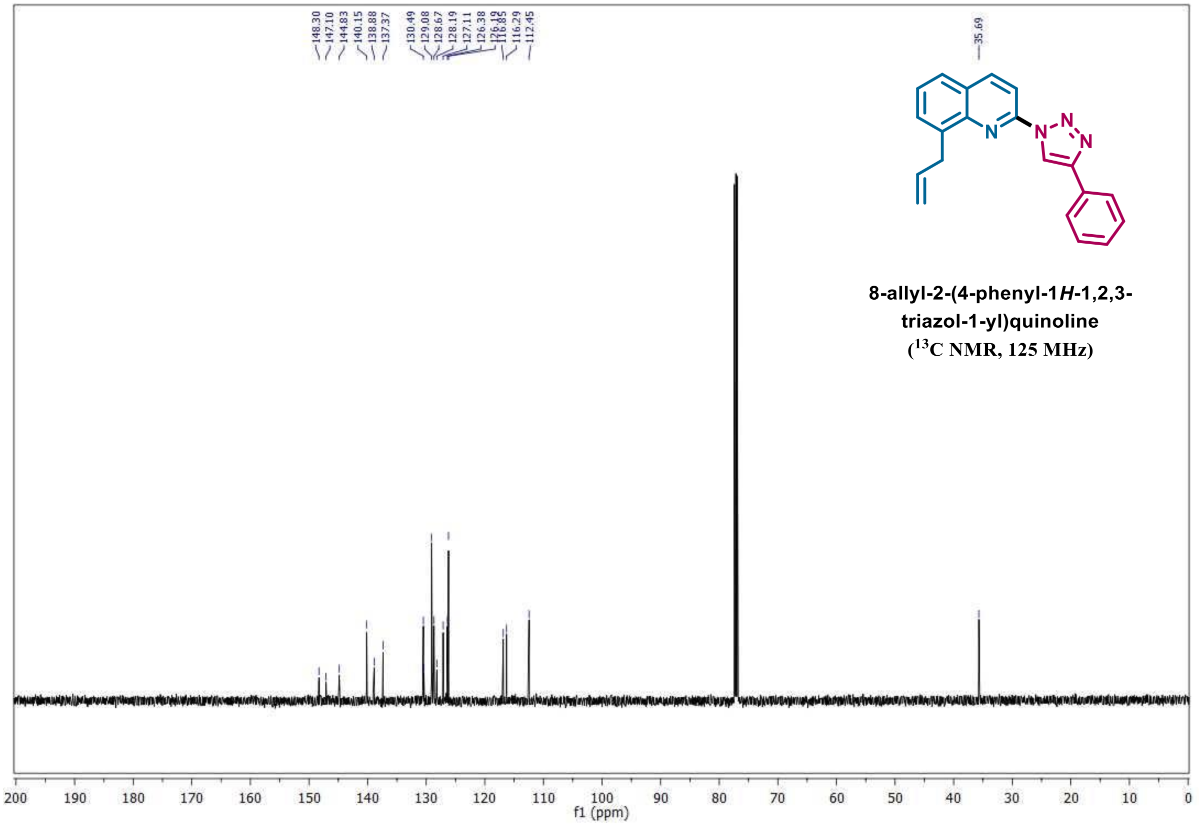

^1H and ^{13}C NMR Spectra of 4a

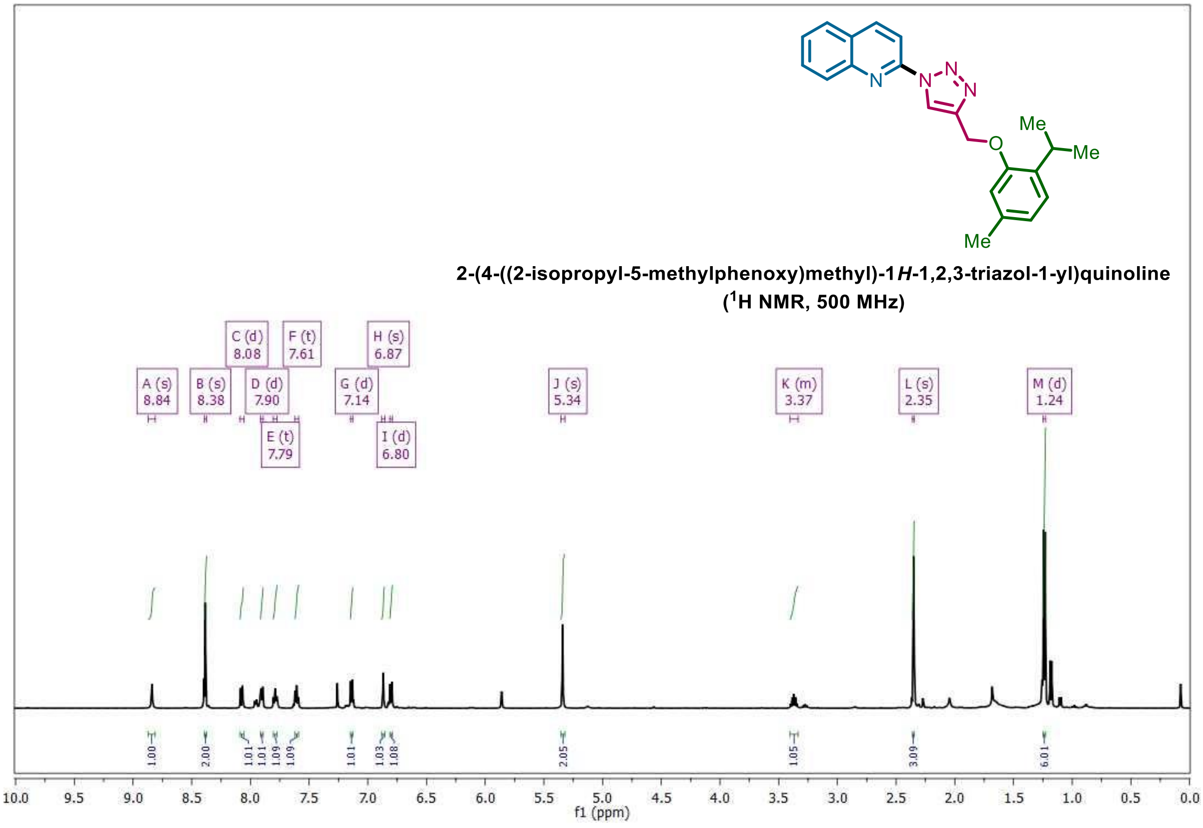

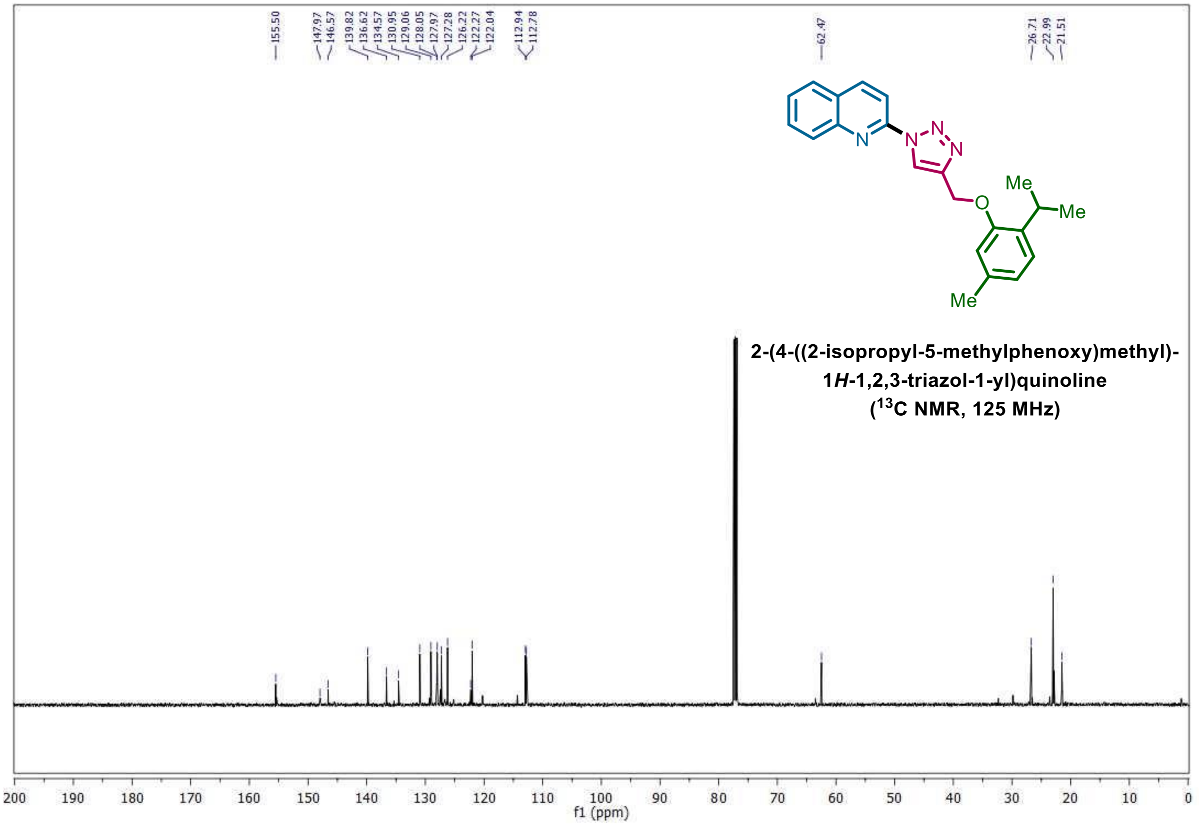

^{1}H and ^{13}C NMR Spectra of 4b

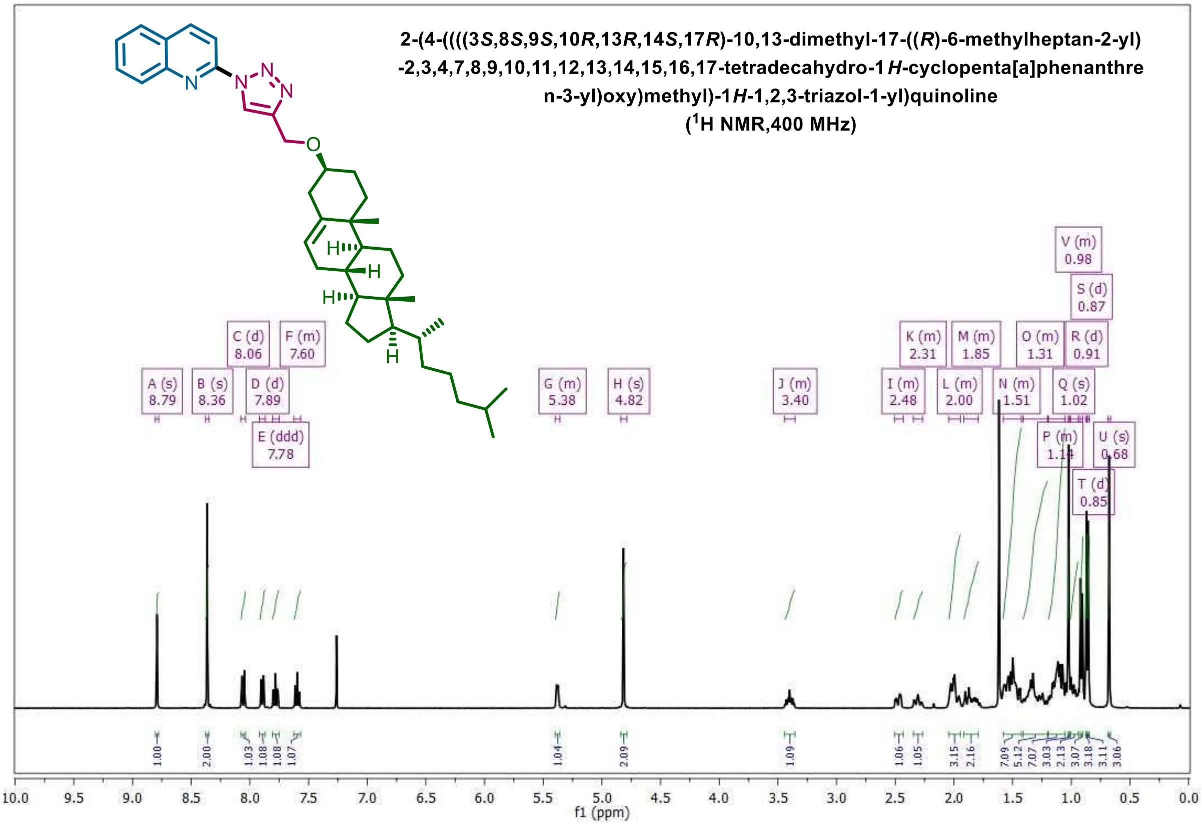

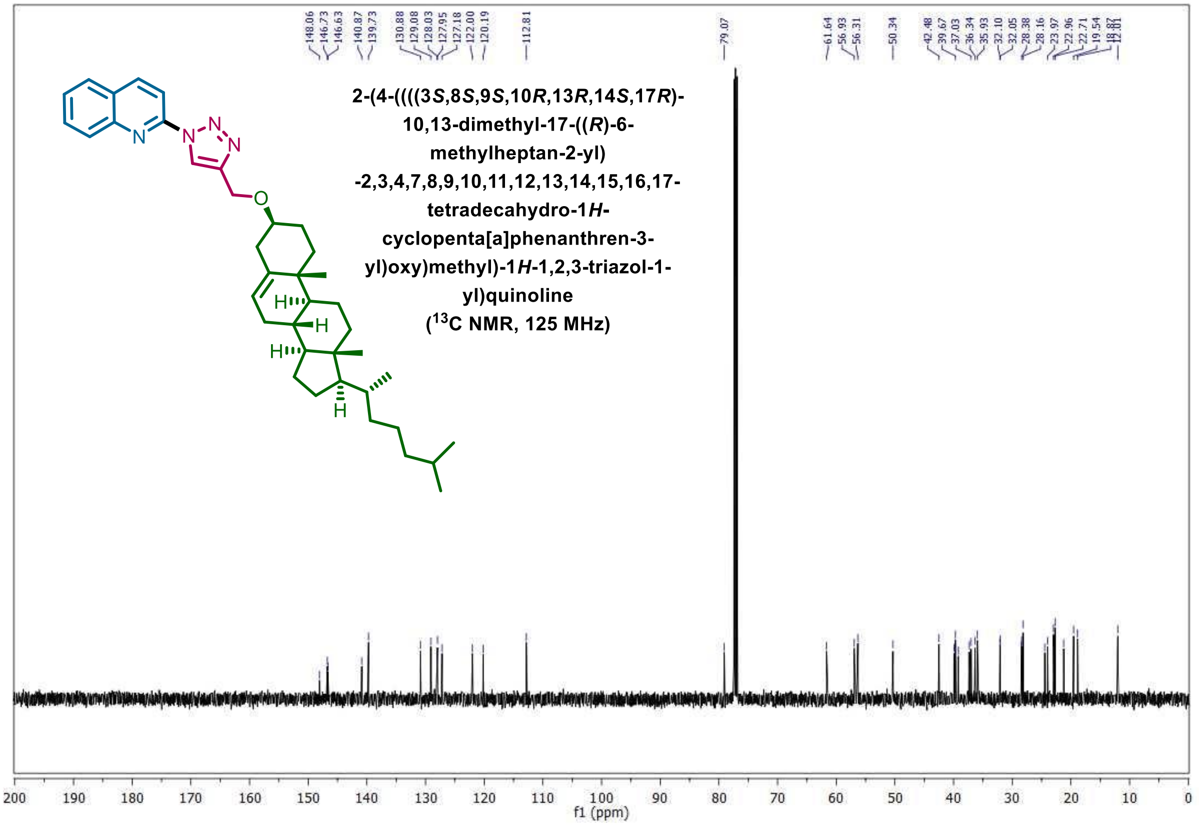

^1H and ^{13}C NMR Spectra of 4c

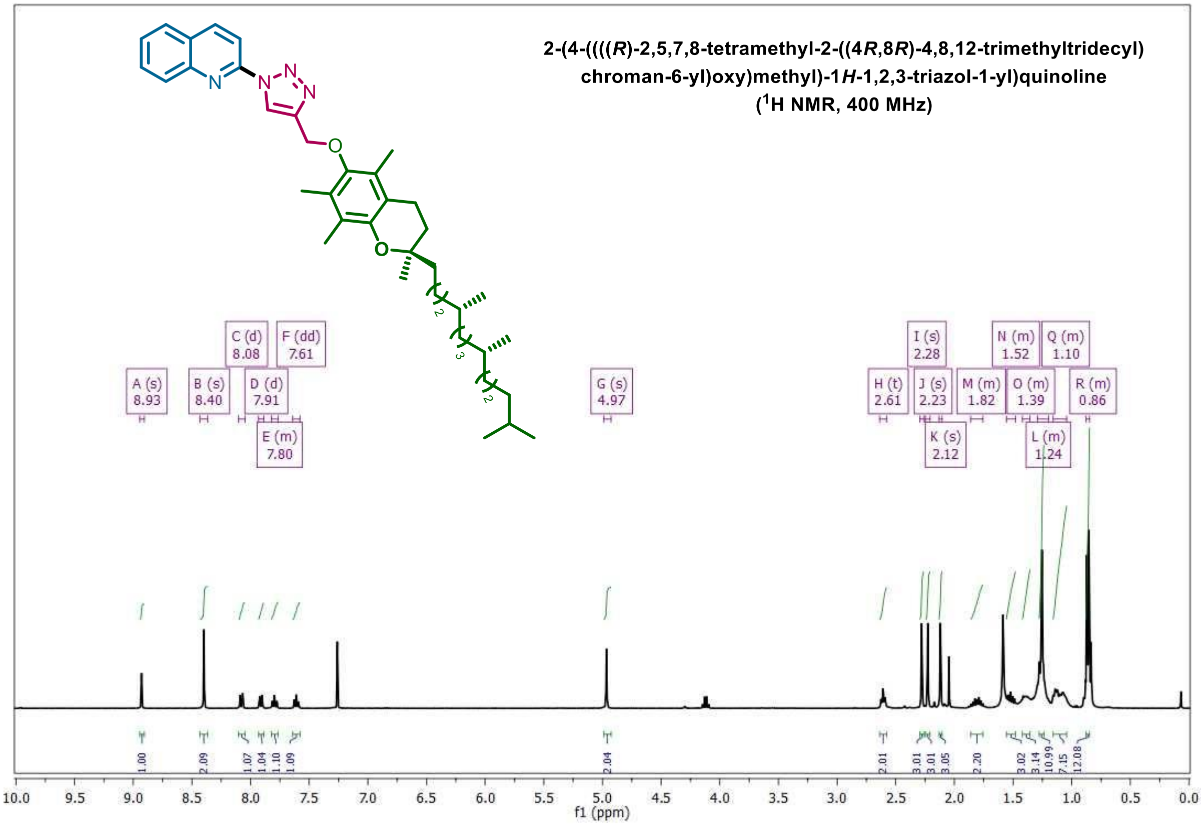

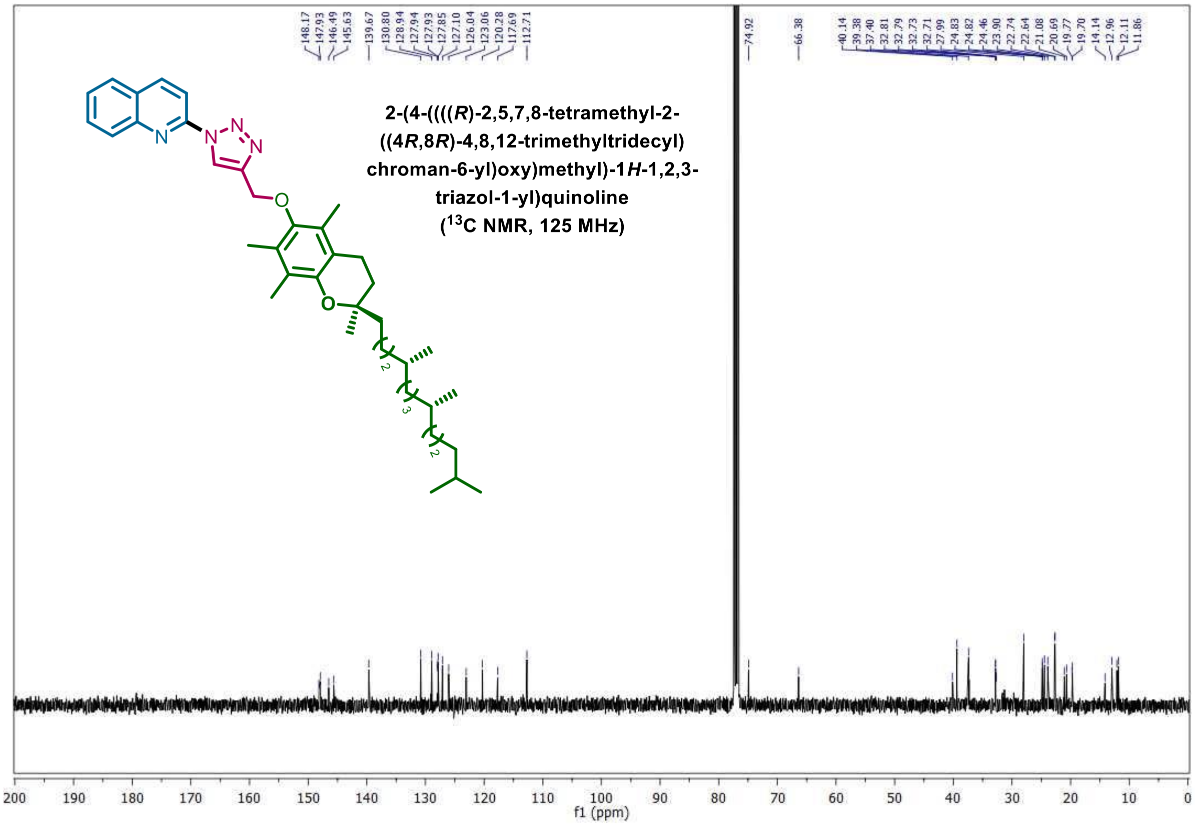

^{1}H and ^{13}C NMR Spectra of 6a

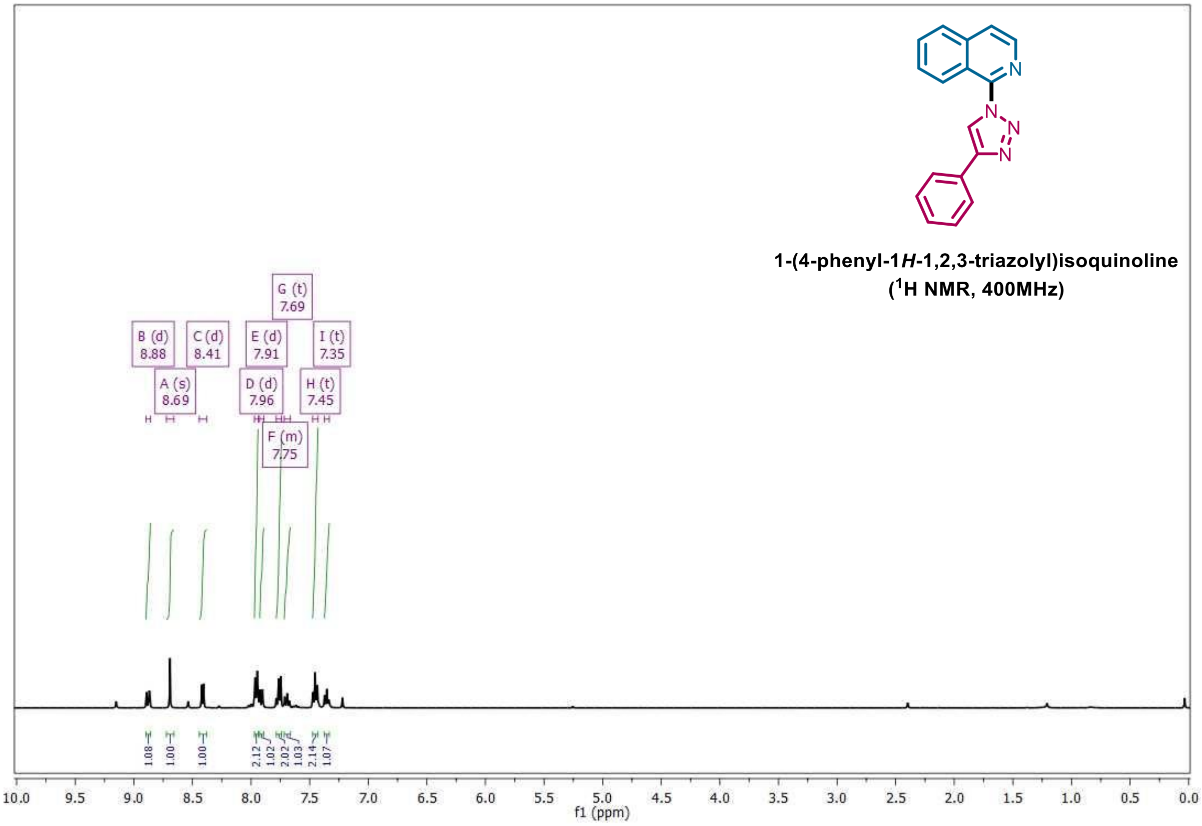

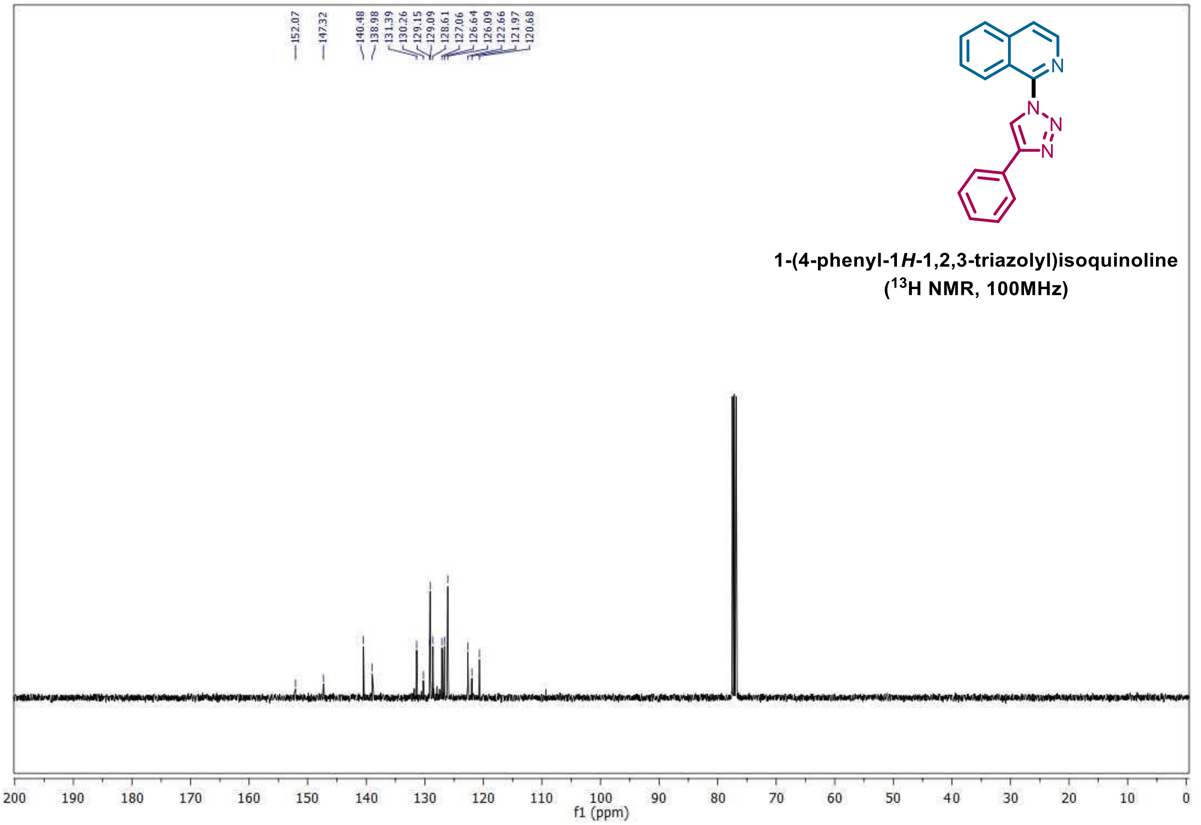

^{1}H and ^{13}C NMR Spectra of 6b

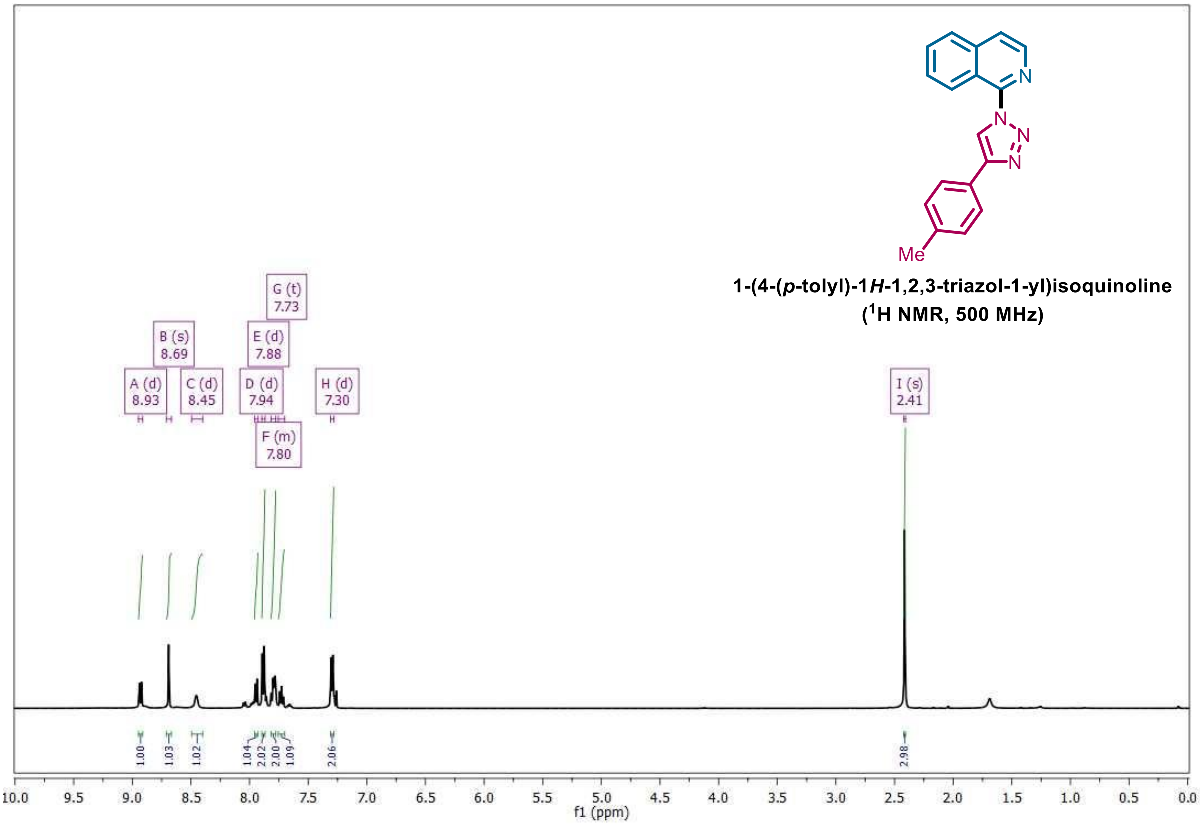

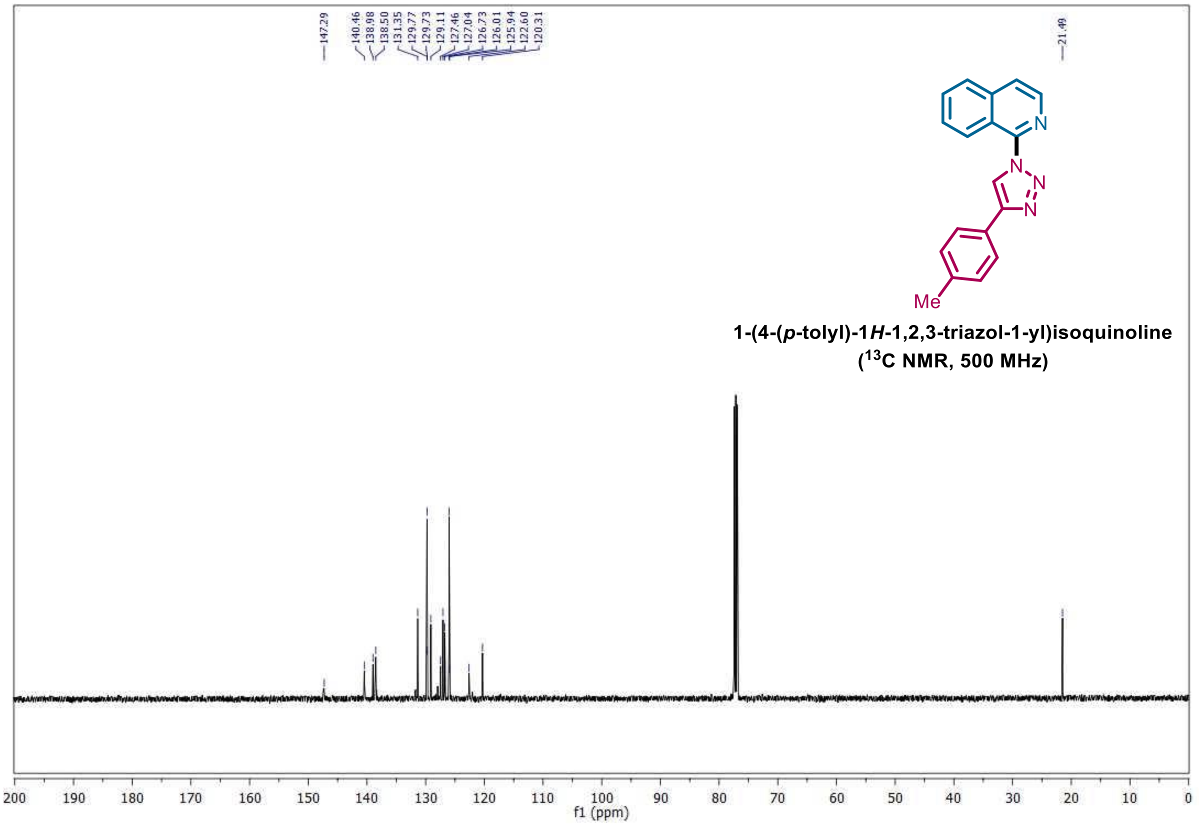

^{1}H and ^{13}C NMR Spectra of 6c

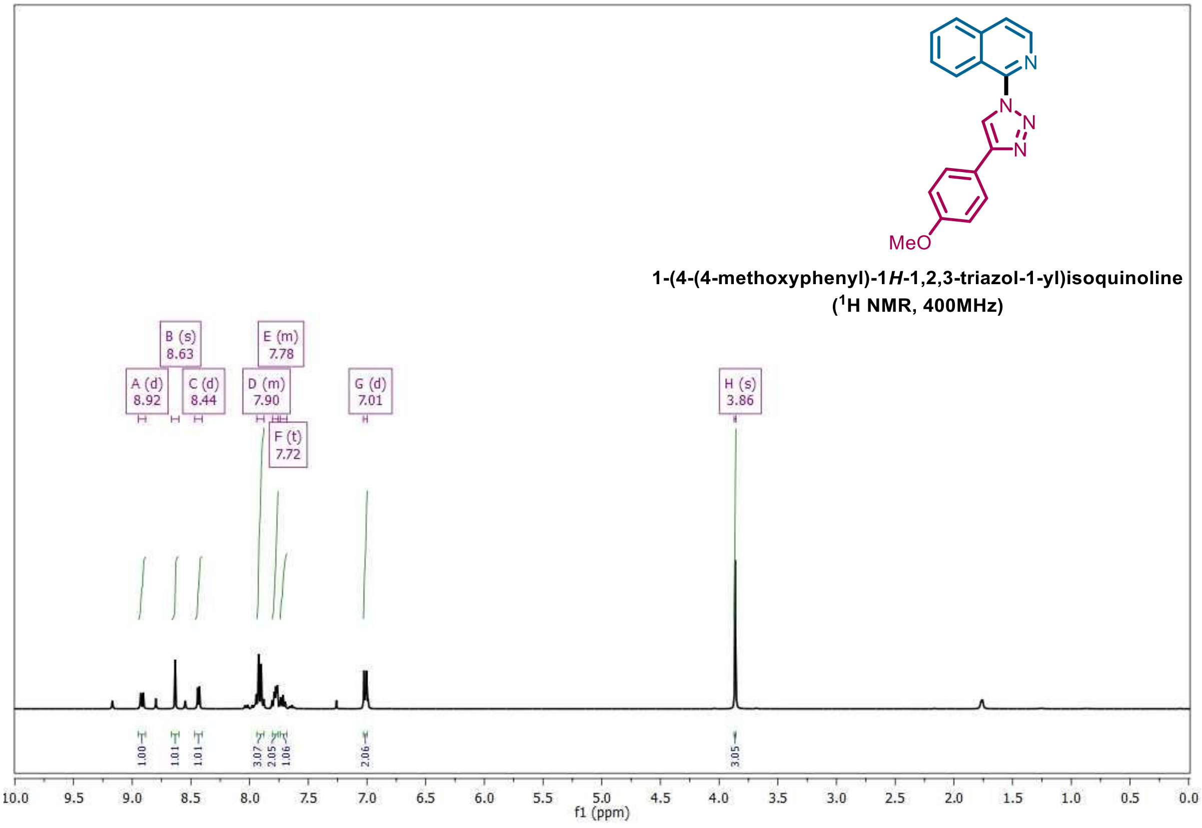

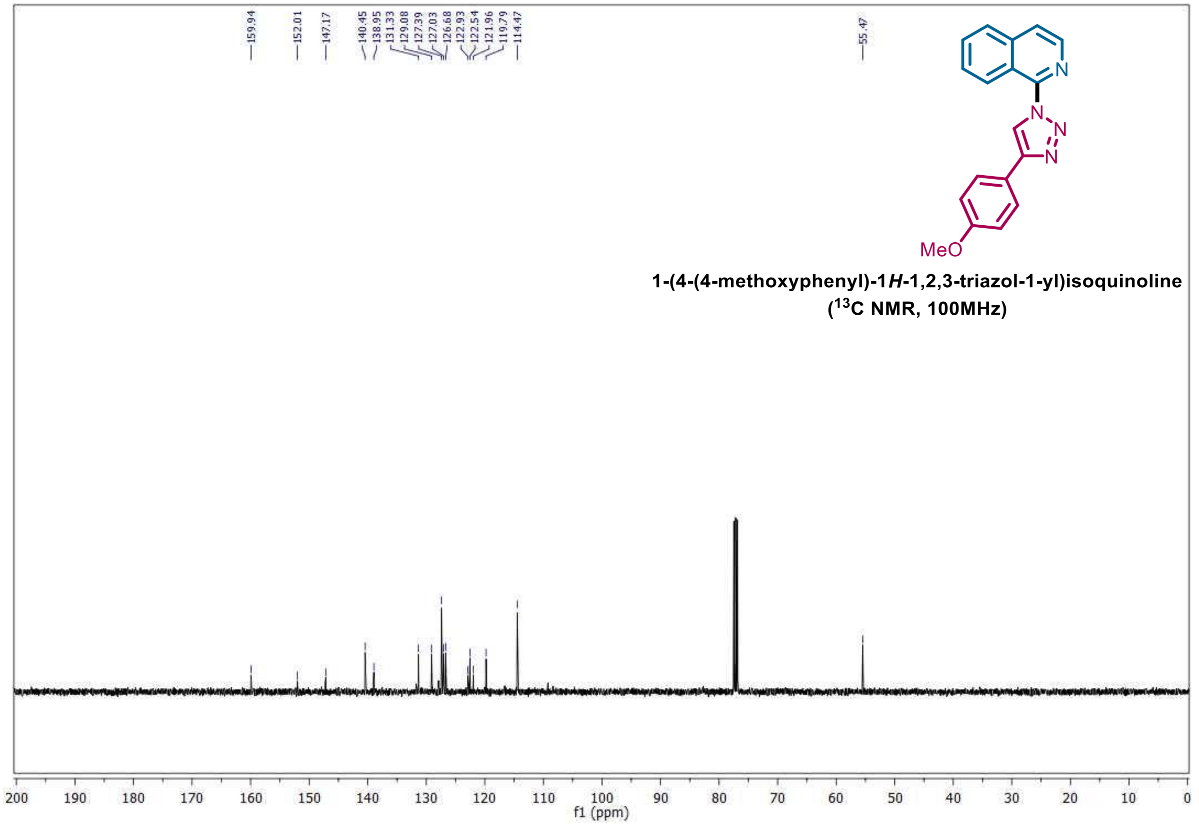

^{1}H and ^{13}C NMR Spectra of 6d

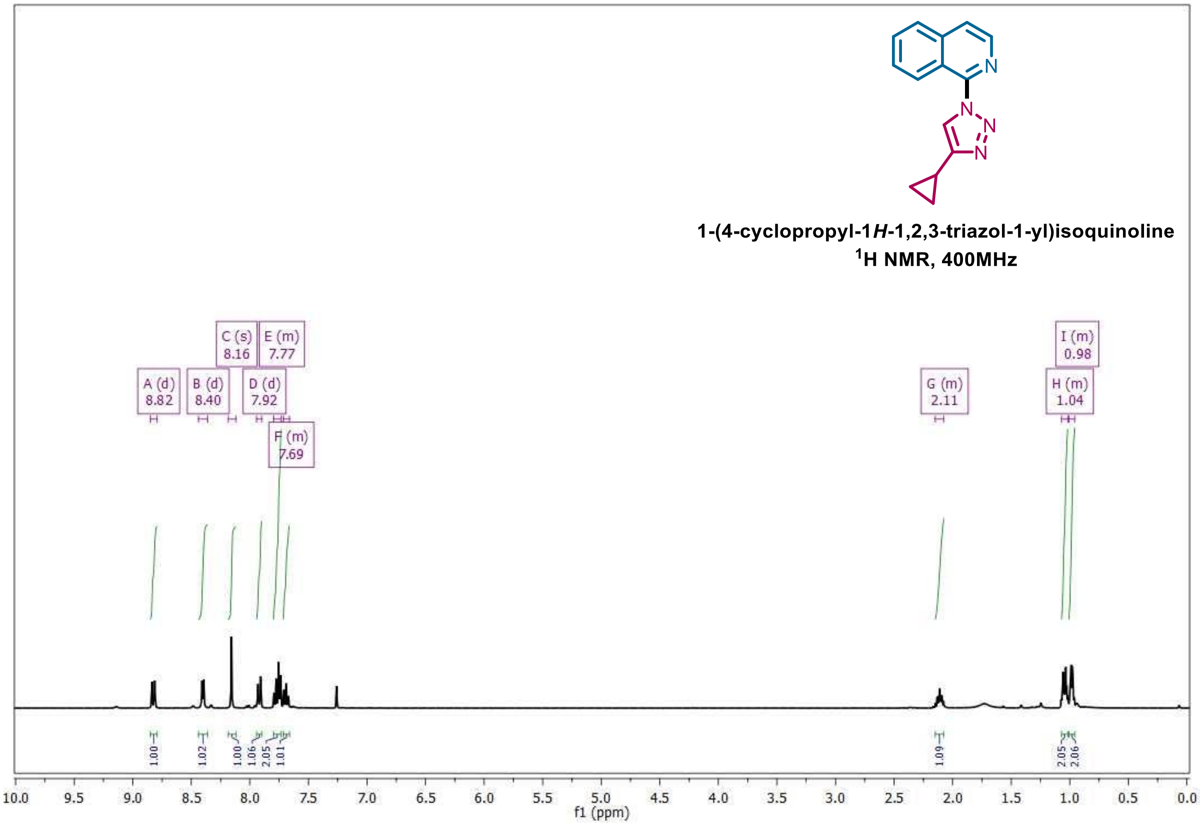

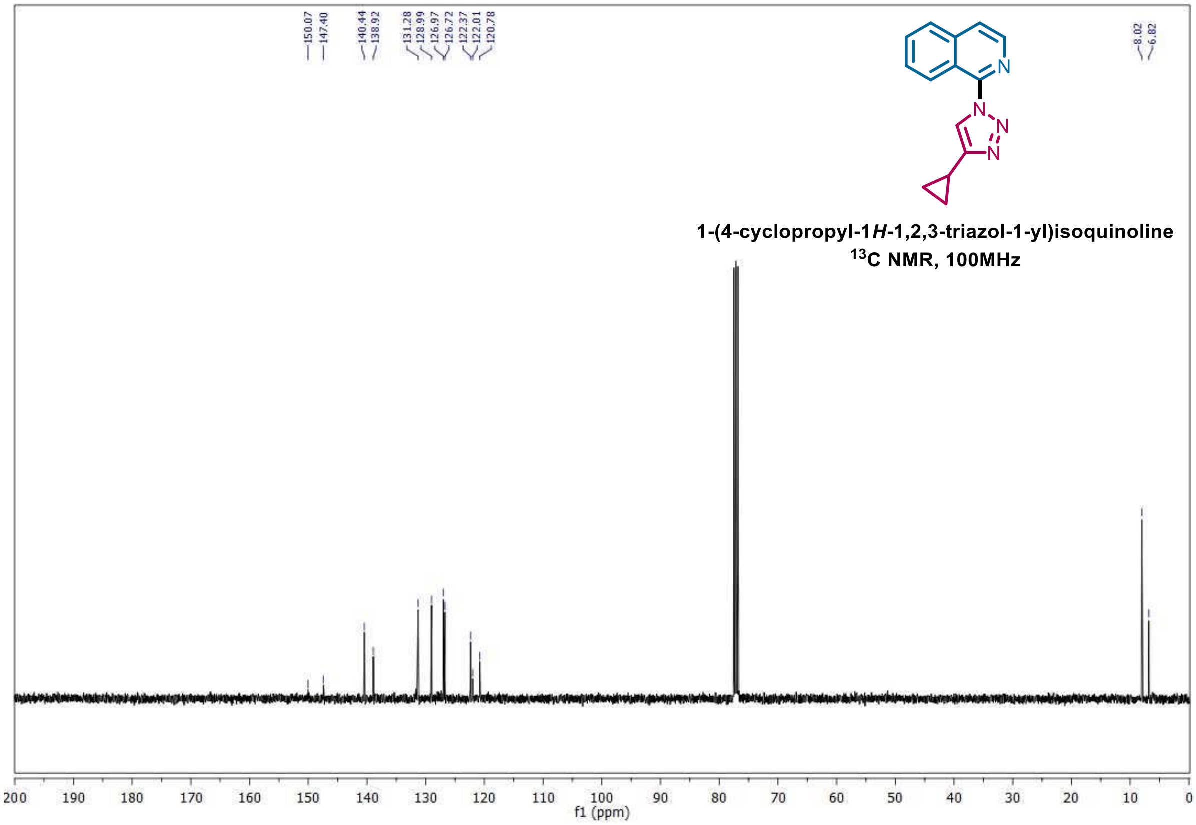

Supplement: File 1 — Experimental details. [file Beilstein_J_Org_Chem-17-485-s001.pdf]
